# Sex-Specific Differences in Clinical Outcomes After Transcatheter Aortic Valve Replacement: A Meta-Analysis of Reconstructed Individual Patient Survival Data

**DOI:** 10.1016/j.shj.2026.101100

**Published:** 2026-07-23

**Authors:** Ali M. Abdelaziz, Mohamedhen Vall Nounou, Ahmad Al Othman, Mirvat Alasnag, Alaide Chieffo, J. Dawn Abbott, Abubaker Al-Allawee, Sofian Zreigh, Mohammed Benhammou, Muhiddin Dervis, Walid Guerguer, Ashraf Haidarah, Rafeek Elmezayen, Mohammad Alzu’bi, Serag Almzainy, Saber Khalid, Qasim Shawesh, Maram Abuajamieh, Muhammed Elhadi, Rodrigo Bagur, Mamas A. Mamas

**Affiliations:** a Faculty of Medicine, Alexandria University, Alexandria, Egypt; b Faculty of Medicine, University of Nouakchott Al Aasrya, Nouakchott, Mauritania; c Department of Medicine, Faculty of Medicine, Near East University, Nicosia, Cyprus; d Cardiac Center, King Fahd Armed Forces Center, Jeddah, Saudi Arabia; e Cardiology Department, Vita Salute San Raffaele University, Milan, Italy; f San Raffaele Interventional Cardiology Unit, IRCCS San Raffaele Scientific Institute, Milan, Italy; g Department of Medicine, Division of Cardiology, Warren Alpert Medical School of Brown University, Providence, Rhode Island, USA; h College of Medicine, University of Fallujah, Fallujah, Iraq; i Faculty of Medicine, Ankara Yildirim Beyazit University, Ankara, Turkey; j Faculty of Medicine, University of Oran 1, Oran, Algeria; k Faculty of Medicine, University of Health Sciences, Algiers, Algeria; l West China Hospital, Sichuan University, Chengdu, Sichuan, China; m Faculty of Medicine, Kafr El-Sheikh University, Kafr El-Sheikh, Egypt; n Faculty of Medicine, The Hashemite University, Zarqa, Jordan; o Faculty of Medicine, University of Tripoli, Tripoli, Libya; p Faculty of Medicine, University of Benghazi, Benghazi, Libya; q Department of Internal Medicine, HCA Mountain View Hospital, Las Vegas, Nevada, USA; r Faculty of Medicine, Cairo University, Cairo, Egypt; s College of Medicine, Korea University, Seoul, South Korea; t Department of Medicine, London Health Sciences Centre, Western University, London, Ontario, Canada; u Keele Cardiovascular Research Group, Centre for Prognosis Research, Keele University, Keele, UK; v National Institute for Health and Care Research (NIHR) Birmingham Biomedical Research Centre, Birmingham, UK

**Keywords:** Aortic stenosis, Cardiovascular disease in women, Sex differences, TAVI, TAVR

## Abstract

**Background:**

Females with severe aortic stenosis present with distinct anatomical and clinical characteristics compared to males, which may influence outcomes following transcatheter aortic valve replacement (TAVR). We sought to investigate the prognostic impact of sex-specific differences on short- and long-term outcomes following TAVR.

**Methods:**

PubMed, Embase, Scopus, and Web of Science were searched through August 2025 for randomized and observational studies reporting sex-specific outcomes after TAVR. Reconstructed individual patient data from published Kaplan–Meier (KM) curves were used to estimate long-term survival outcomes.

**Results:**

Overall, 71 studies involving 481,353 patients were included. Compared with males, females demonstrated a higher short-term risk of adverse outcomes, including 30-day all-cause mortality (hazard ratio [HR]: 1.064, 95% CI: 1.005–1.127; *p* = 0.033) and stroke/transient ischemic attack (HR: 1.511, 95% CI: 1.317–1.734; *p* < 0.001). Females were also more likely to experience life-threatening bleeding (risk ratio [RR]: 1.29, 95% CI: 1.03–1.62; *p* = 0.028), major bleeding (RR: 1.26, 95% CI: 1.13–1.41; *p* < 0.001), and major vascular complications (RR: 1.66, 95% CI: 1.53–1.81; *p* < 0.001). Despite these higher periprocedural risks, females demonstrated superior long-term outcomes, with lower all-cause mortality (HR: 0.812, 95% CI: 0.794–0.831; *p* < 0.001) and cardiovascular mortality (HR: 0.837, 95% CI: 0.784–0.893; *p* < 0.001) during follow-up extending to 10 years, whereas risks of stroke/transient ischemic attack and myocardial infarction were comparable between sexes.

**Conclusions:**

Females undergoing TAVR experience higher early risks of mortality, stroke, bleeding, and vascular complications, but exhibit a sustained long-term survival advantage compared with males, highlighting the need for sex-centered periprocedural management strategies.

## Highlights

• Females had higher 30-day mortality and stroke risk after transcatheter aortic valve replacement (TAVR).
• Females had more bleeding and vascular complications post-TAVR.
• Females showed lower long-term all-cause mortality after TAVR.
• Female long-term survival advantage persisted across valve generations.
• Reconstructed individual patient data meta-analysis included 481,353 TAVR patients.

## Graphical Abstract

Across 71 studies including 481,353 patients, females undergoing TAVR experienced higher early procedural complications and 30-day mortality but showed a sustained long-term survival advantage compared with males, highlighting important sex-specific differences in TAVR outcomes. Abbreviations: HR, hazard ratio; IPD, individual patient data; MI, myocardial infarction; RR, risk ratio; TIA, transient ischemic attack.

**Figure:**
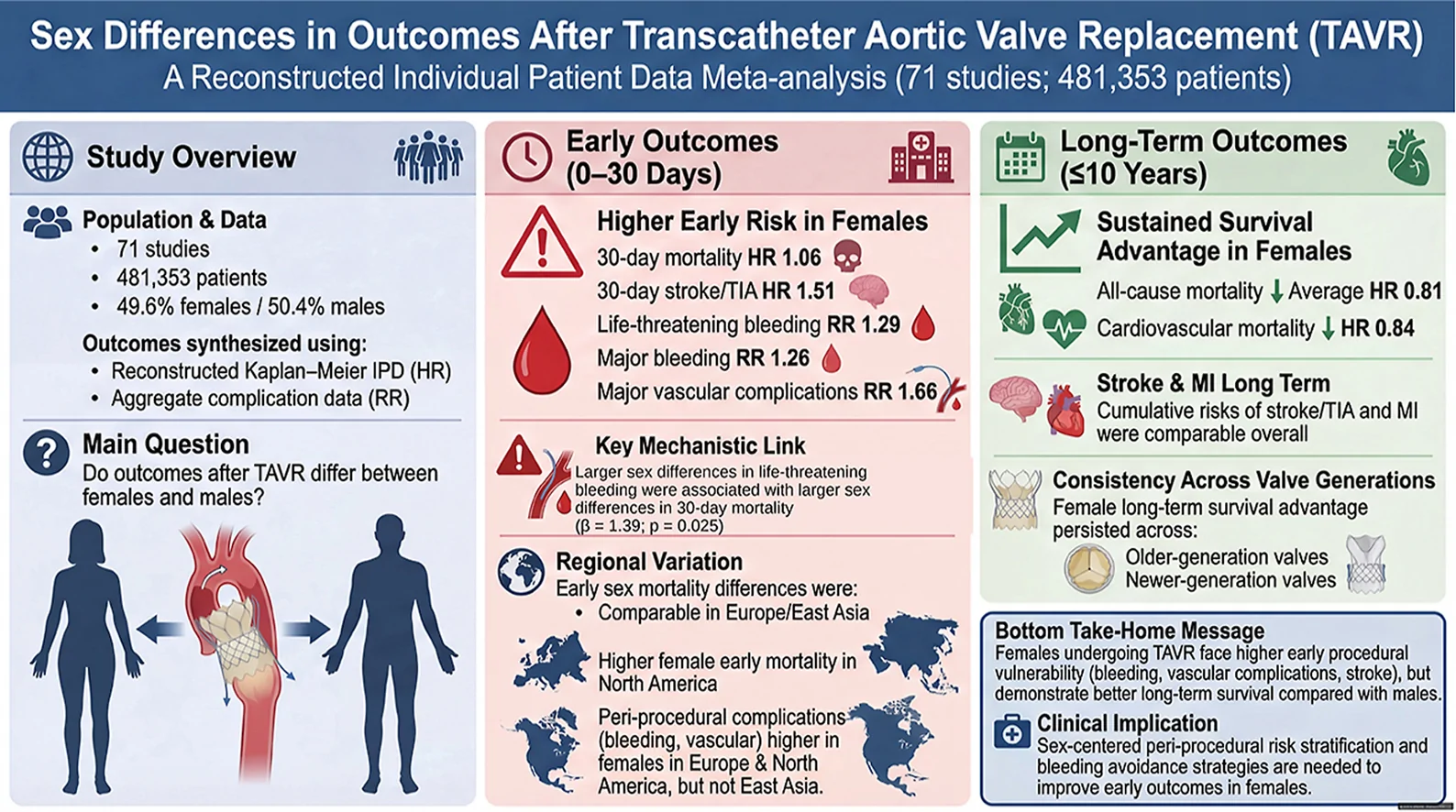

## Introduction

Aortic stenosis (AS) is the most frequent valvular heart disease in developed countries, with a prevalence increasing with age, affecting 2%–3% of individuals over 65 and up to 7% of individuals above 80 years of age.^1^^,^^2^ Left untreated, symptomatic severe AS carries a high mortality rate, with up to 25% of patients dying each year and survival limited to 2–3 years.^3^ Aortic valve replacement, whether surgical or transcatheter, is the only established therapy to improve outcomes. Transcatheter aortic valve replacement (TAVR) has increasingly become a widely adopted treatment option for most patients with severe symptomatic AS, regardless of the surgical risk.^4^^,^^5^

As TAVR became widely adopted, there was growing awareness of potential differences in outcomes, particularly related to sex. Females undergoing TAVR often present with distinct clinical and anatomical characteristics compared to males, including older age, smaller annular dimensions, and narrower peripheral vasculature, all of which may increase procedural complexity and influence outcomes.^6^^,^^7^ In addition, distinct pathological characteristics are observed, with males exhibiting more severe calcification of the aortic valve. At the same time, females have more extensive fibrosis and less severe calcification for the same degree of stenosis. These differences extend to left ventricular remodeling, with females exhibiting concentric hypertrophy, whereas males show eccentric hypertrophy.^8^ Together, these anatomical and pathophysiological distinctions may underlie the divergent procedural risks and long-term outcomes observed between sexes following TAVR. Although current clinical guidelines do not differentiate management strategies based on sex, existing evidence regarding sex-specific outcomes after TAVR remains conflicting.

Recognizing and understanding these differences is increasingly important as TAVR expands into younger, lower-risk populations, where patient selection and individualized procedural strategies may further improve outcomes. Although previous aggregate-data meta-analyses reported sex differences following TAVR, reconstructed individual patient data (IPD) enable more granular time-to-event analyses and landmark assessment of temporal outcome patterns. Therefore, this meta-analysis of reconstructed IPD from published Kaplan-Meier (KM) plots aims to pool all available evidence to assess the impact of sex differences on short- and long-term clinical outcomes following TAVR.

## Methods

This study was conducted following the Preferred Reporting Items for Systematic reviews and Meta-Analyses (PRISMA) (Supplementary Table 1).^9^ Ethical approval was not required, as all data were obtained from published studies. The review protocol is registered in the International Registry of Systematic Reviews (PROSPERO): CRD420251087604.

### Literature Search and Eligibility Criteria

We conducted a comprehensive literature search across PubMed, Embase, Scopus, and Web of Science using relevant search terms from inception until August 2025. We also attempted a manual citation-based search to identify any additional studies that may have been missed. The detailed search strategy is presented in Supplementary Table 2. We included observational (prospective and retrospective) studies, as well as post hoc analyses of clinical trials, providing sex-disaggregated time-to-event mortality and safety outcomes data, along with aggregate summary data on short-term periprocedural complications. We excluded studies that did not report sex-stratified data, studies with overlapping or duplicate data, review articles, case series, expert opinions, conference abstracts, and editorials. Supplementary Table 3 shows some potentially relevant studies that were excluded, along with reasons for exclusion.

### Screening and Selection of Studies

All records were imported into covidence for screening. Titles/abstracts and full texts were reviewed independently in duplicate, with disagreements resolved by senior adjudication. Reasons for exclusion were documented, and the study selection process is summarized in Supplementary Figure 1.

### Outcomes of Interest and Data Extraction

Primary outcomes were all-cause and cardiovascular mortality. Secondary outcomes included stroke/transient ischemic attack (TIA), myocardial infarction (MI), rehospitalization, heart failure hospitalization, permanent pacemaker implantation, and periprocedural complications (Valve Academic Research Consortium [VARC]-defined bleeding and vascular events, acute kidney injury [AKI], and tamponade). Data were extracted independently in duplicate using a standardized form, with discrepancies resolved by consensus and senior adjudication.

### Risk of Bias and Quality Assessment

The risk of bias for each included study was assessed independently and in duplicate using the Newcastle–Ottawa Scale,^10^ with any discrepancies reconciled by consensus.

### Statistical Analysis

All analyses were performed using R software (version 4.5.1; R Foundation for Statistical Computing, Vienna, Austria). For the meta-analysis of reconstructed IPD, we followed the curve approach, which reconstructs IPD from published KM curves.^11^^,^^12^ We applied the algorithm described by Liu et al.^13^ based on the R package IPDfromKM (version 0.1.10). First, the raw data coordinates, consisting of time and survival probabilities, were extracted from each KM curve, along with the corresponding risk tables, to reconstruct the IPD. In those instances where published KM plots lacked risk tables, we used an alternative algorithm described by Rogula et al.^14^ based on the R package (KMtoIPD, version 1.0.0), which relies on the censoring times instead of the number of subjects at risk at corresponding time points alongside the extracted coordinates to yield reconstructed IPD. Ultimately, the reconstructed IPD from all studies was pooled together to form the study dataset. The accuracy of IPD reconstruction was assessed by visual inspection of each reconstructed curve against the original one, as well as by the log-rank test and the calculation of the root mean squared error, mean absolute error, and maximum absolute error. Pooled reconstructed IPD from all studies was plotted for each outcome in a pooled KM plot using the R packages (survival, version 3.8.3) and (*survminer*, version 0.5.0). Hazard ratio (HR) with its 95% CI was computed using a Cox regression model. Pooled KM curves were truncated at 10 years to ensure stable estimates and adequate numbers of patients at risk. The proportional hazards (PH) assumption for each Cox model was assessed using Schoenfeld residuals and the Grambsch-Therneau test. When the PH assumption was violated, we estimated time-varying HRs with 95% CI at each follow-up time point using the R package rstpm2 (version 1.6.7) and flexible parametric survival models. We modeled the baseline HR using a spline with 4 degrees of freedom, comprising 3 intermediate knots and 2 boundary knots, placed at the quartiles of the event distribution. As reconstructed IPD recover event times and censoring information but do not recover individual-level baseline covariates, all HRs derived from reconstructed IPD analyses should be interpreted as unadjusted sex-specific estimates.

For outcomes for which no KM plots were available, event rates were pooled in a Mantel-Haenszel random-effects model to compute risk ratios (RRs) with 95% CIs using the R package meta (version 8.1-0). Statistical heterogeneity was assessed using Cochran’s Q test and quantified using the I^2^ statistic. We performed leave-one-out sensitivity analysis to test for a disproportional effect of any single study on the pooled effect estimate. All statistical tests were 2 sided, and *p* < 0.05 denoted statistical significance.

#### Landmark, Sensitivity, and Subgroup Analyses

Landmark analyses were estimated using separate Cox PHs models restricted to the prespecified time points (30 days, 1 year, 5 years, and 10 years) to reflect standard early, intermediate, midterm, and longer-term follow-up intervals used in TAVR outcome reporting. Sensitivity analyses were performed to assess the robustness of the primary findings. Survival analyses were repeated after restricting to secondary analyses of randomized controlled trials (RCTs) when feasible. In addition, sensitivity analyses were conducted, including only studies that provided numbers at risk beneath KM plots, to reduce uncertainty from reconstruction approaches that rely on censoring-time estimation. Subgroup analyses were undertaken to explore heterogeneity, including stratification by valve generation (older versus newer devices) when extractable, given ongoing evolution in valve design and implantation techniques. Meta-regression analyses were also performed to examine whether baseline comorbidities modified sex-specific outcome differences, using univariable models whenever there were at least 10 studies per covariate to avoid overfitting.

### Publication Bias

We assessed publication bias using funnel plots and Egger regression test whenever there were at least 10 studies for each outcome, as required for reliable results.

## Results

### Included Studies and Study Characteristics

Seventy-one studies (6 of which were post hoc analyses of RCTs) comprising 481,353 patients were included (Supplementary Tables 4 and 5), with 238,614 (49.6%) females and 242,739 (50.4%) males. At baseline, females were significantly older (mean difference = +1; 95% CI: 0.77 to 1.23; *p* < 0.001) and had higher Society of Thoracic Surgery (STS) predicted risk of mortality scores than males (mean difference = +0.65; 95% CI: 0.49 to 0.81; *p* < 0.001). In relation to comorbidities, as compared with males, females had higher prevalence of hypertension (odds ratio [OR] = 1.11, 95% CI: 1.06 to 1.17, *p* < 0.001), but lower prevalence of atrial fibrillation (OR = 0.9, 95% CI: 0.86 to 0.94, *p* < 0.001), diabetes (OR = 0.85, 95% CI: 0.81 to 0.89, *p* < 0.001), chronic obstructive pulmonary disease (COPD) (OR = 0.73, 95% CI: 0.68 to 0.79, *p* < 0.001), peripheral artery disease (OR = 0.622, 95% CI: 0.58 to 0.67, *p* < 0.001), prior MI (OR = 0.53, 95% CI: 0.46 to 0.6, *p* < 0.001), prior stroke (OR = 0.81, 95% CI: 0.76 to 0.87, *p* < 0.001), prior percutaneous coronary intervention (OR = 0.6, 95% CI: 0.55 to 0.66, *p* < 0.001) and prior permanent pacemaker (PPM) implantation (OR = 0.71, 95% CI: 0.65 to 0.79, *p* < 0.001). Echocardiographic assessment revealed that females had a significantly higher mean left ventricular ejection fraction at baseline (mean difference = +4.36, 95% CI: 3.59 to 5.14, *p* < 0.001) as well as higher transvalvular gradient (mean difference = +3.86 mmHg, 95% CI: 3.22 to 4.5, *p* < 0.001) but a lower mean aortic valve area (mean difference = −0.07 cm^2^, 95% CI: −0.08 to −0.06, *p* < 0.001). Supplementary Tables 4 and 5 provide an overview summary of the included studies, along with their respective risk of bias assessments. Baseline clinical characteristics of patients in each study are presented in Supplementary Table 6. Frailty was reported in a subset of studies (n = 11) and was consistently more prevalent or more severe among females. However, quantitative pooling was not feasible due to heterogeneous frailty indices (Supplementary Table 7). Supplementary Table 8 shows the valve generations across studies.

### Meta-Analysis of the Primary Outcomes

#### All-Cause Mortality

**Figure 1:**
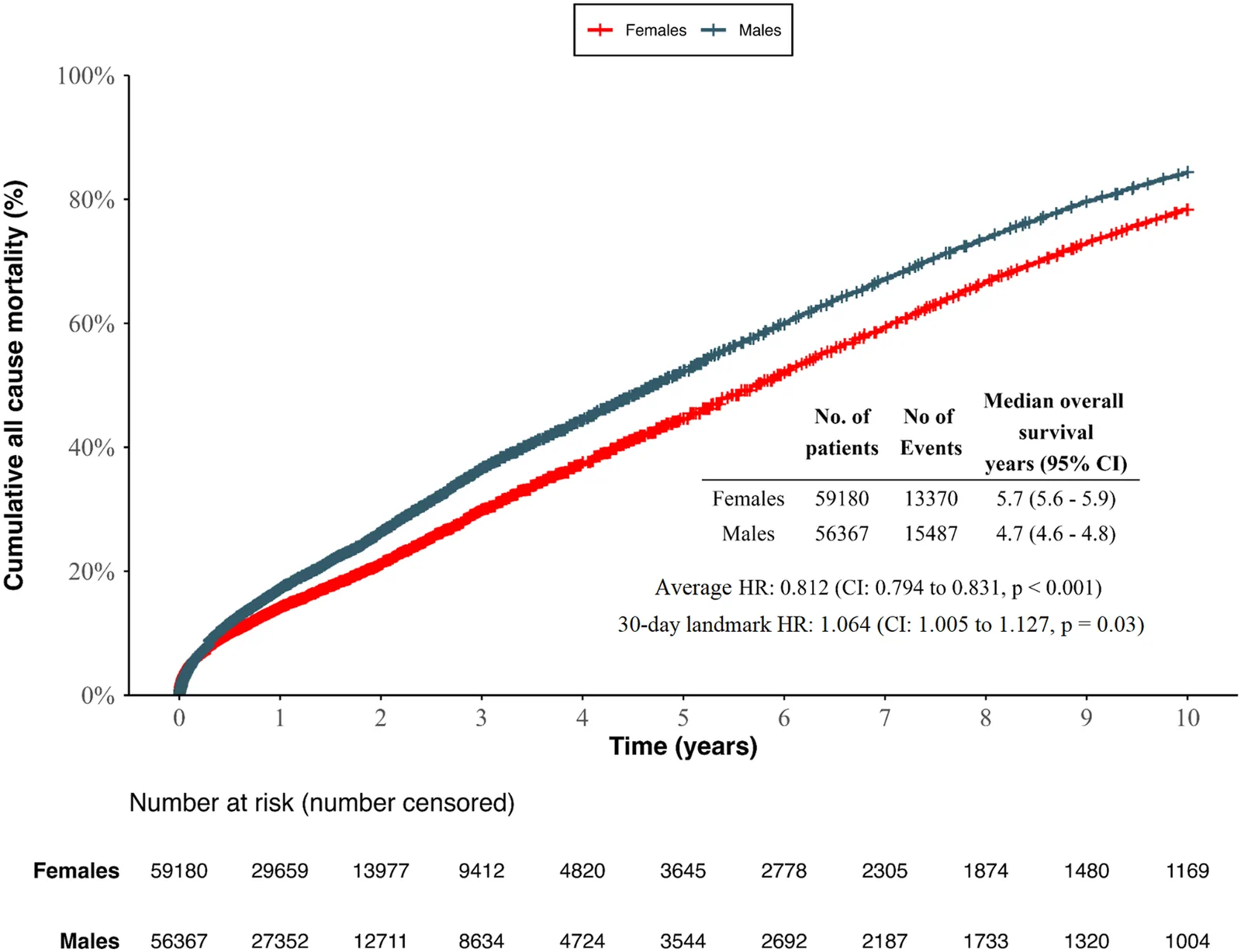
**All-cause mortality after the TAVR procedure.** Abbreviations: HR, hazard ratio; TAVR, transcatheter aortic valve replacement.

Reconstructed IPD from 45 studies (59,180 females and 56,367 males) were pooled to evaluate all-cause mortality after TAVR (Supplementary Table 9). In the long-term follow-up, truncated at 10 years, females showed significantly better survival than males (HR: 0.812, 95% CI: 0.794-0.831; *p* < 0.001; Figure 1). Landmark analyses suggested that this survival advantage emerged beyond the early postprocedural phase (Table 1). At 1 year, females had a 17.4% lower mortality risk compared with males (HR: 0.826, 95% CI: 0.801 to 0.852; *p* < 0.001). In contrast, within the first 30 days after TAVR, females experienced a small but statistically significant increase in mortality risk (HR: 1.064, 95% CI: 1.005 to 1.127; *p* = 0.033) (Supplementary Table 10). In the primary pooled Cox model, the PHs assumption was not violated (Grambsch–Therneau test *p* = 0.205). As a sensitivity analysis, a study-stratified Cox model was also performed to account for study-level differences in baseline hazards (Supplementary Table 11). Because the study-stratified model demonstrated evidence of non-PHs (Grambsch–Therneau test *p* = 0.015), flexible parametric survival models were additionally used to explore potential temporal variation in HRs. Sensitivity analyses restricted to studies reporting risk tables beneath KM curves (n = 36) yielded consistent results, with females maintaining a long-term survival advantage (HR: 0.781, 95% CI: 0.756 to 0.807; *p* < 0.001, Supplementary Figure 3). Importantly, the overall pattern remained consistent in sensitivity analyses restricted to randomized trial data sets with available KM curves (n = 4), which again showed improved long-term survival in females (HR: 0.8, 95% CI: 0.73 to 0.9; *p* < 0.001; Supplementary Figure 4). Similarly, subgroup analyses by valve generation (older-generation only: 9 studies; newer-generation only: 4 studies) showed no significant sex difference in 30-day mortality in either generation, although females continued to demonstrate better long-term survival across device generations (Supplementary Figure 5). Subgroup analysis by geographical location suggested distinct patterns in sex-specific mortality after TAVR, with females exhibiting better long-term survival across all regions. However, a landmark analysis showed that at 1-month post-TAVR, mortality was comparable between the sexes in East Asia (n = 6 studies) and Europe (n = 20 studies), although females appeared to exhibit a higher risk of mortality in North America (n = 8 studies) (Supplementary Figures 6-8).

**Table 1:** Landmark analysis of sex-specific mortality and stroke outcomes at clinically relevant time points

Outcome	30-d HR (95% CI, *p* value)	1-y HR (95% CI, *p* value)	5-y HR (95% CI, *p* value)	10-y HR (95% CI, *p* value)
All-cause mortality	1.064 (1.005 – 1.127, *p* = 0.03)	0.826 (0.801 – 0.852, *p* < 0.001)	0.808 (0.788 – 0.828, *p* < 0.001)	0.812 (0.794 – 0.831, *p* < 0.001)
CV mortality	0.967 (0.857 – 1.09, *p* = 0.58)	0.85 (0.785 – 0.921, *p* < 0.001)	0.837 (0.784 – 0.895, *p* < 0.001)	0.837 (0.784 – 0.893, *p* < 0.001)
Stroke/TIA	1.511 (1.317 – 1.734, *p* < 0.001)	1.155 (1.05 – 1.27, *p* = 0.003)	1.043 (0.967 – 1.125, *p* = 0.275)	1.037 (0.967 – 1.111, *p* = 0.3)

Abbreviations: CV, cardiovascular; HR, hazard ratio; TIA, transient ischemic attack.

Meta-regression analyses identified several study-level factors that modified the magnitude of the sex-specific mortality difference. Among all baseline clinical characteristics and comorbidities examined, only diabetes (*p* = 0.022) and chronic kidney disease (CKD) (*p* = 0.036) emerged as statistically significant effect modifiers. Specifically, the female-to-male mortality HR tended to increase in studies with a higher overall prevalence of diabetes, suggesting greater relative vulnerability among females in more diabetic populations. In contrast, the HR tended to decrease with increasing CKD burden, indicating attenuation of the female survival advantage in cohorts with more advanced renal disease. In addition, larger baseline sex imbalances in procedural risk and pulmonary disease were associated with poorer outcomes in females: greater differences in mean STS score and COPD prevalence between females and males were each linked to an increased mortality risk in females (STS: B = 1.106, 95% CI: 1.023 to 1.197, *p* = 0.012; COPD: B = 1.014, 95% CI: 1.000–1.027, *p* = 0.045) (Supplementary Figures 9 and 10).

#### Cardiovascular Mortality

**Figure 2:**
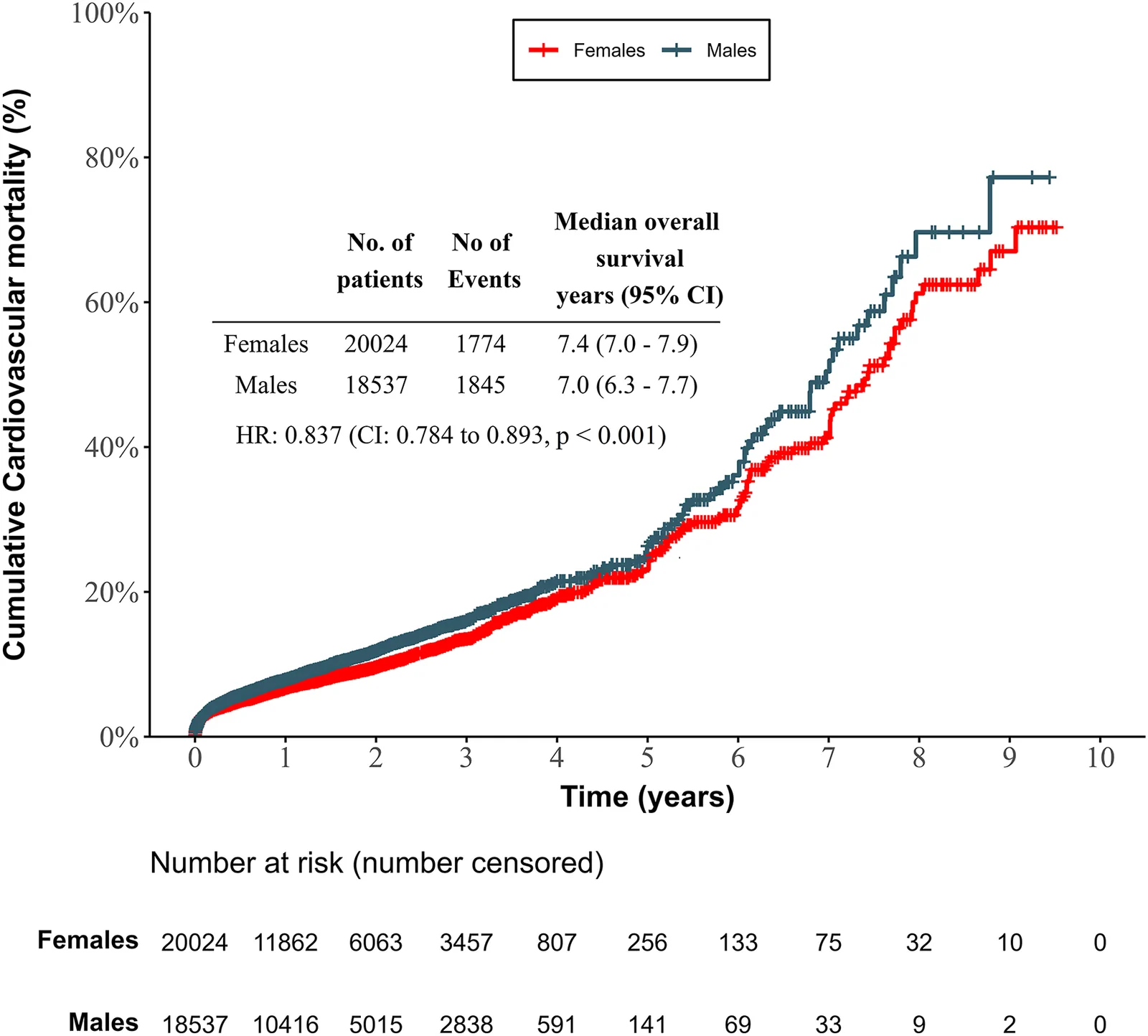
**Cardiovascular mortality after the TAVR procedure.** Abbreviations: HR, hazard ratio; TAVR, transcatheter aortic valve replacement.

Reconstructed IPD from 10 studies, all of which provided numbers at risk beneath the published KM curves, were pooled to assess cardiovascular mortality after TAVR (20,024 females and 18,537 males; Supplementary Table 12). Over the available 10-year follow-up, females experienced significantly better cardiovascular survival than males, with a 16.3% lower risk of cardiovascular death (HR: 0.837, 95% CI: 0.784 to 0.893; *p* < 0.001; Figure 2). Landmark analyses showed that this advantage was already evident by 1 year after TAVR (HR: 0.85, 95% CI: 0.785 to 0.921; *p* < 0.001), whereas no significant sex difference was observed in early cardiovascular mortality at 30 days (HR: 0.967, 95% CI: 0.857 to 1.09; *p* = 0.582; Supplementary Table 13). The PHs assumption was met (Grambsch–Therneau test *p* = 0.288; Supplementary Table 14). Sensitivity analyses restricted to randomized trial data sets or stratified by valve generation were limited by the few eligible studies (2 RCTs, 2 older-generation valve studies, and a single newer-generation study). Regional subgroup analyses showed that females had consistently lower long-term cardiovascular mortality than males in both East Asian (n = 3 studies) and European cohorts (n = 5 studies). However, no significant sex difference was observed at 30 days in either region, and no North American data were available for this endpoint (Supplementary Figure 11).

### Meta-Analysis of the Secondary Outcomes

#### Stroke/TIA

**Figure 3:**
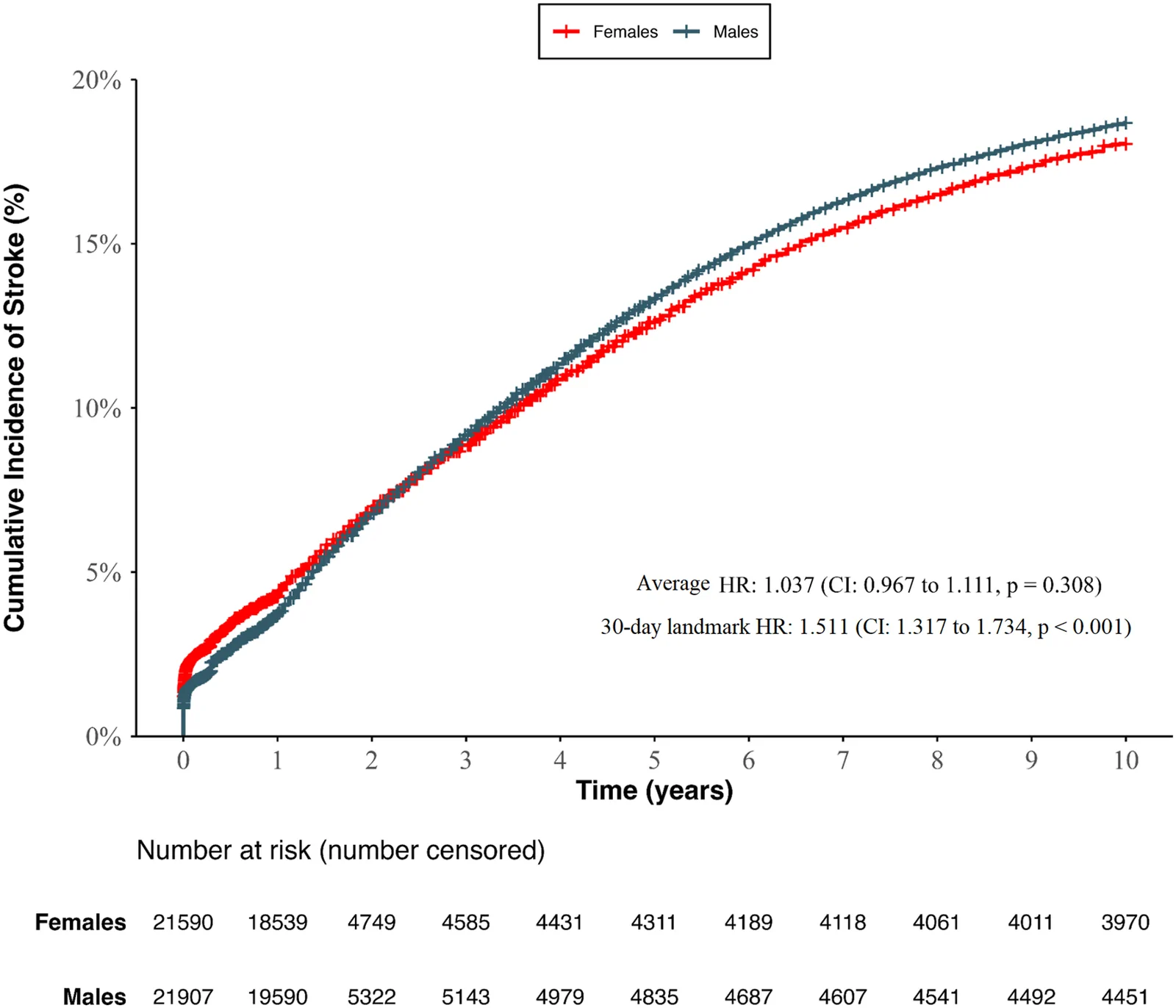
**Stroke/TIA after the TAVR procedure.** Abbreviations: HR, hazard ratio; TAVR, transcatheter aortic valve replacement; TIA, transient ischemic attack.

Reconstructed IPD from 8 studies (21,590 females and 21,907 males) were pooled to evaluate stroke and TIA after TAVR (Supplementary Table 15). Over follow-up truncated at 10 years, the cumulative long-term risk of stroke/TIA was comparable between females and males (HR: 1.037, 95% CI: 0.967 to 1.111; *p* = 0.30; Figure 3). However, landmark analyses demonstrated a higher early stroke/TIA risk among females, with a 51.1% increase at 30 days (HR: 1.511, 95% CI: 1.317 to 1.734; *p* < 0.001) and a 15.5% increase at 1 year (HR: 1.155, 95% CI: 1.05 to 1.27; *p* = 0.003) (Supplementary Table 16). At 2 years, females continued to exhibit a modest but statistically significant excess stroke/TIA risk (HR: 1.101, 95% CI: 1.008 to 1.2; *p* = 0.032); however, from the 3-year landmark onward, no significant sex-based difference was observed, suggesting that the disparity is largely confined to the early and intermediate postprocedural period. The PHs assumption was violated (Grambsch–Therneau test *p* = 0.004; Supplementary Table 17); therefore, time-varying HRs were additionally estimated using flexible parametric survival models (Supplementary Figure 12). Sensitivity analysis restricted to studies reporting published risk tables beneath KM curves (n = 7) yielded consistent findings, with no significant difference in cumulative stroke/TIA risk between sexes (HR: 1.093, 95% CI: 0.894 to 1.337; *p* = 0.40; Supplementary Figure 13). Additional sensitivity analyses restricted to randomized trial data sets or stratified by valve generation were limited by the few eligible studies (1 RCT, 1 older-generation valve study, and 2 newer-generation studies). Geographic patterns for stroke/TIA were less consistent. In North American cohorts (n = 3 studies), females demonstrated a higher cumulative stroke/TIA risk over the available follow-up, whereas in European studies (n = 3 studies), females had an excess early risk at 30 days with no significant sex difference during later follow-up (Supplementary Figure 14). These findings should be interpreted cautiously, given the limited numbers in regional studies.

#### Myocardial Infarction

Reconstructed IPD from 3 studies, including 17,507 females and 18,264 males, were pooled to assess the cumulative incidence of MI after TAVR (Supplementary Table 18). Over long-term follow-up, no significant sex-based difference in cumulative MI risk was observed (HR: 1.032, 95% CI: 0.935–1.138; *p* = 0.535), and landmark analyses similarly showed no differences at clinically relevant early time points (Supplementary Table 19). The PHs assumption was violated for this endpoint (Grambsch–Therneau test *p* = 0.006; Supplementary Table 20); therefore, time-varying HRs were additionally estimated using flexible parametric survival models (Supplementary Figure 15).

**Figure 4:**
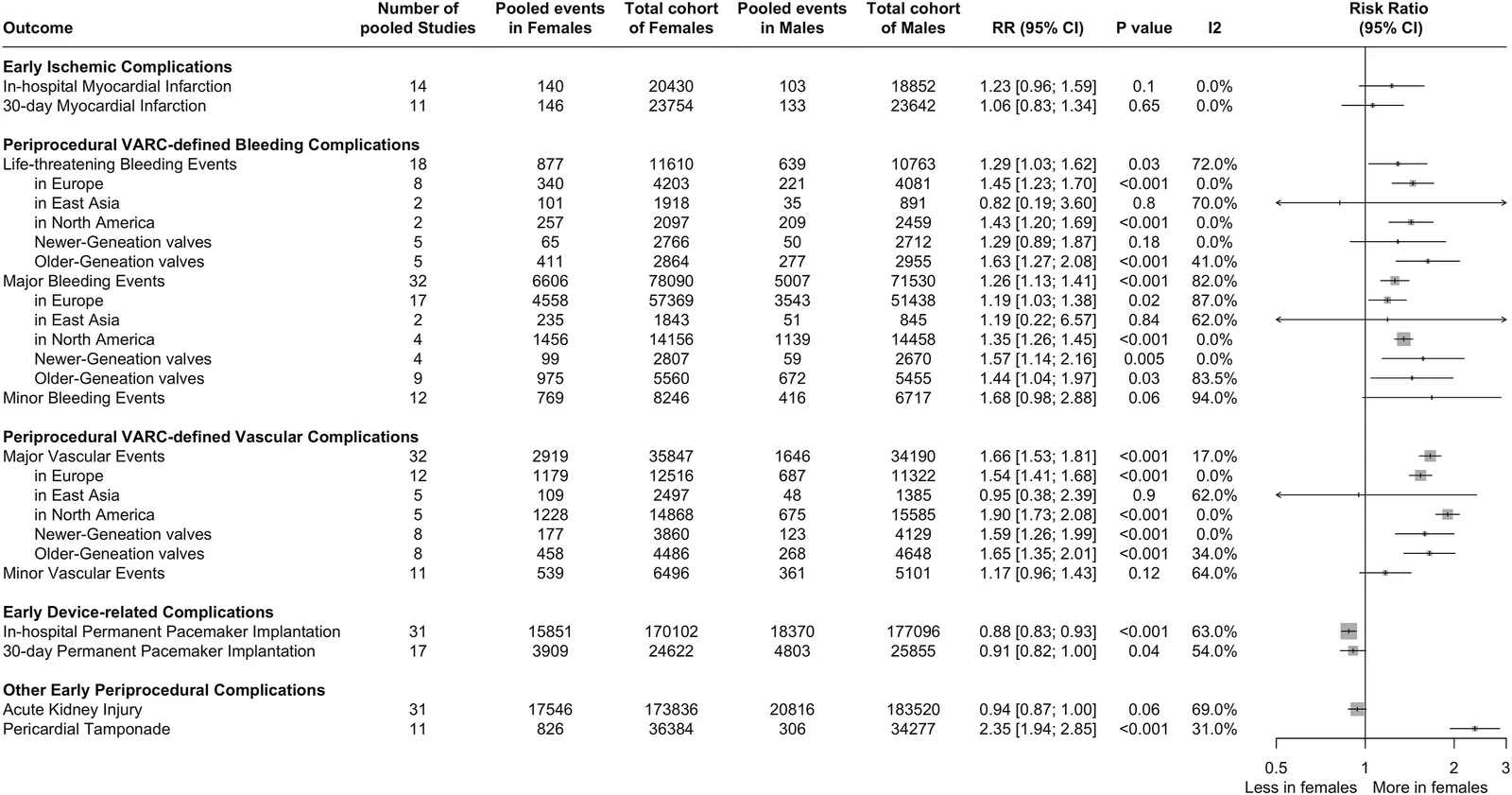
**Early procedural complications after TAVR.** Summary forest plot comparing early periprocedural and 30-day complication risks in females versus males, including bleeding events, vascular complications, myocardial infarction, acute kidney injury, cardiac tamponade, and permanent pacemaker implantation. Detailed outcome-specific forest plots are presented in Supplementary Figures 18-22. Abbreviations: RR, risk ratio; TAVR, transcatheter aortic valve replacement; VARC, Valve Academic Research Consortium.

Given the limited availability of KM curves for MI, we also examined early event rates using aggregate study-level data (Figure 4). These pooled analyses demonstrated comparable risks of MI between females and males both during the index hospitalization (RR: 1.23, 95% CI: 0.96–1.59) and at 30 days (RR: 1.06, 95% CI: 0.83–1.34), supporting the absence of meaningful sex differences in MI following TAVR.

#### Rehospitalizations

Reconstructed IPD from 4 studies (56,050 females and 56,393 males) were pooled to evaluate all-cause rehospitalizations after TAVR (Supplementary Table 21). In these exploratory analyses, females had a modestly higher risk of rehospitalization than males at the available 3 years of follow-up (HR: 1.04, 95% CI: 1.008 to 1.072; *p* = 0.012; Supplementary Table 22). The PHs assumption was violated for this endpoint (Grambsch–Therneau test *p* = 0.02; Supplementary Table 23), and time-varying HRs were therefore estimated using flexible parametric survival models (Supplementary Figure 16). Given the small number of contributing KM data sets and the heterogeneity in follow-up duration across studies, these findings should be interpreted as hypothesis-generating rather than definitive.

Similarly, heart failure–related hospitalizations were evaluated using reconstructed IPD from 3 studies (6373 females and 6363 males) (Supplementary Table 24). Females showed a slightly higher long-term risk of heart failure hospitalization compared with males (HR: 1.081, 95% CI: 1.005–1.162; *p* = 0.036; Supplementary Table 25). Non-PHs were again observed (Grambsch–Therneau test *p* = 0.01; Supplementary Table 26), and time-varying HRs were estimated accordingly (Supplementary Figure 17). As with all-cause rehospitalizations, inference for this outcome remains limited by the small number of available KM curves, variable follow-up horizons, and sparse aggregate reporting, and should therefore be considered exploratory.

#### Early Procedural Complications After TAVR

Random-effects meta-analysis revealed that, compared with males, females experienced lower rates of PPM implantation during the periprocedural in-hospital period post-TAVR (9.3 vs. 10.4%, RR: 0.88, 95% CI: 0.83 to 0.93, *p* < 0.001, I2 = 62.8%, Figure 4). In terms of periprocedural complications, the incidence of AKI post-TAVR was comparable between the cohorts, with a nonsignificant trend toward lower incidence in females (10.1 vs. 11.3%, RR: 0.94, 95% CI: 0.87 to 1, *p* = 0.062, I2 = 69.2%, Figure 4). Females experienced higher rates of pericardial tamponade (2.3 vs. 0.89%, RR: 2.35, 95% CI: 1.94 to 2.85, *p* < 0.001, I^2^ = 31.3%, Figure 4), VARC-defined life-threatening bleeding events (7.6 vs. 5.9%, RR: 1.29, 95% CI: 1.03 to 1.62, *p* = 0.028, I^2^ = 72.3%, Figure 4), VARC-defined major bleeding events (8.5 vs. 7%, RR: 1.26, 95% CI: 1.13 to 1.41, *p* < 0.001, I^2^ = 82.4%, Figure 4), and VARC-defined major vascular complications (8.1 vs. 4.8%, RR: 1.66, 95% CI: 1.53 to 1.81, *p* < 0.001, I^2^ = 16.8%, Figure 4). No significant difference was observed regarding VARC-defined minor bleeding events or minor vascular complications (9.3 vs. 6.2%, RR: 1.68, 95% CI: 0.98 to 2.88, *p* = 0.06, I^2^ = 93.5%, Figure 4 and 8.3 vs. 7%, RR: 1.17, 95% CI: 0.96 to 1.43, *p* = 0.12, I^2^ = 63.8%, Figure 4 respectively).

Leave-one-out sensitivity analyses confirmed the robustness of the results with no disproportionate effect of any single study on any pooled effect estimate, except for AKI, which became modestly lower in females. (Supplementary Figures 23-27).

Visual inspection of funnel plots, along with Egger regression test results, revealed no concern for publication bias for any of the outcomes. (Supplementary Figures 28-32).

Meta-regression revealed that the older the population, the greater the RR for life-threatening bleeding events (*p* = 0.001) and major bleeding events (*p* = 0.022), and the higher the overall prevalence of hypertension (*p* = 0.041) and overall mean STS score (*p* = 0.01), the greater the RR for major vascular complications (Supplementary Figures 33-35). Subgroup analysis based on geographical region revealed a higher risk of life-threatening and major bleeding as well as major vascular events in females in both Europe and North America, with comparable risks in East Asia (Supplementary Figure 36). Subgroup analysis of older versus newer generation valves revealed a consistently higher risk of major bleeding and vascular complications in females, but comparable rates of life-threatening bleeding events with newer generation valves (Supplementary Figures 31-39). To explore whether sex differences in periprocedural complications contributed to the observed early mortality patterns, we performed meta-regression analyses regressing the RR for 30-day mortality (female vs. male) on the corresponding RRs for major bleeding, life-threatening bleeding, major vascular complications, and stroke, each in a univariate model. Among these, only life-threatening bleeding was significantly associated with differences in 30-day mortality (β = 1.394, 95% CI = 1.043 to 1.863, *p* = 0.025; Supplementary Figure 40).

## Discussion

This meta-analysis provides a comprehensive evaluation of sex differences across 11 outcomes after TAVR, pooling data from 71 studies involving 481,353 patients. The data span multiple countries and continents and include studies from the inception of TAVR through 2025. Overall, females had higher 30-day mortality than males; however, by 1 year and beyond, females had better long-term survival. Furthermore, females had higher risks of in-hospital procedural bleeding, vascular complications, and stroke, while showing lower rates of PPM implantation and comparable rates of AKI.

Regarding mortality, previous studies have yielded conflicting results. This inconsistency is further exacerbated by the emergence of new studies and the discrepancies observed in prior systematic reviews, even those focused exclusively on newer-generation valves. Although Bozso et al.^15^ observed higher early mortality rates among females, consistent with our findings, they found no significant difference in mortality at 1 year. Conversely, Lang et al.^16^ reported a higher mortality risk in males at 1 year post-TAVR, which aligns with our findings.

The observed differences between our study and prior systematic reviews may stem from several key factors. Our study's larger sample size reduces selection bias and enhances the generalizability of our findings. In addition, by incorporating more recent studies in this rapidly evolving field, where complication rates have significantly decreased over time, our analysis reflects advancements in procedural expertise, postprocedural care, and patient selection criteria.^17^ These improvements are likely to have mitigated the sex-based differences reported in earlier studies.

It is evident from our landmark analysis findings that, although females face higher procedural risks and greater early mortality after the procedure, their risk becomes comparable to that of males after the first month. Over time, they exhibit better survival outcomes. Although this is most likely a reflection of the global trend of more prolonged survival in females in the general population compared with males,^18^ it can also be attributed to distinct characteristics of the female sex, which are believed to increase the risk of early mortality and certain perioperative complications. For instance, females have smaller vessels and a larger sheath-to-vessel diameter ratio, which increases the difficulty of sheath insertion.^19^^,^^20^ In addition, females have smaller left ventricular cavities, greater ventricular wall thickness, and narrow outflow tracts.^21^

Furthermore, there is a higher prevalence of pulmonary hypertension in females,^22^ which is itself a significant independent predictor of 30-day mortality post-TAVR.^23^ Interpretation of the observed female survival advantage should also account for baseline differences between the sexes. As shown in Supplementary Table 6, females were generally older. They had higher STS predicted risk of mortality scores but demonstrated lower prevalence of several adverse comorbidities, including diabetes mellitus, COPD, peripheral artery disease, prior MI, and prior percutaneous coronary intervention.

Diabetes and CKD emerged as effect modifiers of sex-based mortality after TAVR. A higher diabetes burden was associated with larger female-versus-male mortality differences at the study level, whereas a higher CKD burden appeared to attenuate the female survival advantage. These exploratory findings should be considered hypothesis-generating and warrant confirmation in patient-level studies before informing risk stratification or clinical decision-making. In addition, Nakase et al.^24^ demonstrated that the pattern of cardiac damage secondary to AS differed by sex, with females having a lower 5-year mortality risk in the early stages of cardiac damage, whereas in more advanced stages, the mortality risk becomes comparable.

The observed geographic variation in early sex-specific outcomes after TAVR likely reflects differences in patient selection, procedural practice, and periprocedural management across health care systems. Although females demonstrated a consistent long-term survival advantage across regions, early mortality disparities were less uniform, with comparable 30-day outcomes in Europe and East Asia but modestly higher early mortality among females in North American cohorts. Regional differences were also evident for periprocedural complications: life-threatening bleeding, major bleeding, and major vascular complications were consistently more frequent in females in both European and North American studies, whereas no significant sex differences were observed in East Asian cohorts. These findings suggest that system-level factors, including vascular access strategies, bleeding avoidance protocols, and device-era adoption, may modulate early female vulnerability. Importantly, these subgroup analyses are exploratory and subject to ecological confounding, and should therefore be interpreted as hypothesis-generating rather than causal.

These anatomical and physiological challenges are further exacerbated by systemic disparities that females face in the health care system. Females undergoing TAVR are often older at the time of the procedure, which may result from delays in referral, underdiagnosis, undertreatment of cardiovascular conditions, and delays in the clinical presentation of AS in females.^25^ Females are also significantly underrepresented in major TAVR clinical trials.^26^ This underrepresentation may stem from exclusion based on factors such as age, health conditions, or anatomical differences.^27^ Furthermore, the reliance on a single set of treatment guidelines for both sexes can lead to suboptimal care, as these guidelines may not account for sex-specific anatomical, physiological, and psychosocial differences.^15^

Despite the challenges females face in the short term, several factors are hypothesized to affect their long-term survival differently than those for men. Females generally have fewer baseline comorbidities, such as coronary artery disease and diabetes, which are known to worsen long-term outcomes, and they also have a longer baseline life expectancy.^20^ Their smaller body size and aortic annuli reduce the risk of patient-prosthesis mismatch, improving valve function and hemodynamics postprocedure.^28^ Combined with more favorable left ventricular remodeling and recovery patterns, these advantages likely contribute to the improved long-term survival rates observed in females post-TAVR. Geographical variations in early mortality after TAVR may reflect differences in practice, perioperative care, and patient characteristics. Higher 30-day mortality among North American females, unlike in Europe or East Asia, highlights the need to contextualize sex-based disparities within health care systems.

### Implications for Future Research

Future research should prioritize strategies to reduce early periprocedural vulnerability in females undergoing TAVR, with particular emphasis on mitigating bleeding and vascular complications. Updated valvular heart disease guidelines and risk models should incorporate sex-specific anatomical and clinical factors to support individualized procedural planning. Moreover, improving the representation of females in prospective trials is essential to generate robust evidence for tailored interventions and optimize outcomes across sexes.

### Limitations

This study has several limitations. First, most included studies were observational, and many were retrospective, introducing potential residual confounding, selection bias, and differences in clinician-driven referral patterns between sexes. Although reconstructed IPD improves time-to-event synthesis, it does not provide the complete individual-level covariate structure required for propensity-based adjustment, and the observed associations should not be interpreted as causal. Frailty is an important potential confounder: although females were consistently frailer in studies reporting frailty measures, heterogeneous assessment tools precluded quantitative pooling. Second, we made every effort to minimize overlap between included cohorts, but exclusions were performed at the study level, and residual duplication across linked registries cannot be fully excluded. Third, outcome definitions evolved with changes in VARC criteria and valve-generation technology, potentially introducing variability in complication reporting. Fourth, cardiovascular mortality analyses were based on cause-specific KM data and did not account for competing noncardiovascular deaths, potentially overestimating the cumulative incidence of cardiovascular mortality. Fifth, multiple secondary, subgroup, landmark, and meta-regression analyses were performed without adjustment for multiple comparisons, increasing the possibility of type I error, particularly for borderline statistically significant findings. Finally, although pooled KM curves were truncated at 10 years to improve stability, long-term estimates remain influenced by fewer contributing studies. They should therefore be interpreted cautiously despite the presentation of risk tables and supplementary curve validation.

## Conclusion

Compared with males undergoing TAVR, females experience higher early risks of mortality, stroke, bleeding, and vascular complications. However, this excess early hazard diminishes beyond the first month, and females demonstrate a sustained long-term survival advantage during follow-up.

## Funding

The authors have no funding to report.

## Declaration of Generative AI and AI-Assisted Technologies in the Writing Process

AI tools (OpenAI ChatGPT-5.2 version) were used solely to enhance the clarity, readability, and language of the manuscript. These tools did not contribute to the conception or design of the study, the data analysis, the interpretation of results, or the generation of any scientific content. Their use was limited exclusively to improving language and presentation. All content was thoroughly reviewed and verified by the authors to ensure accuracy and uphold scientific integrity.

## Ethics Statement

This study was conducted following the Preferred Reporting Items for Systematic reviews and Meta-Analyses (PRISMA). Ethical approval was not required, as all data were obtained from published studies. The review protocol is registered in the International Registry of Systematic Reviews (PROSPERO): CRD420251087604.

## Disclosure Statement

The authors report no conflict of interest.
