# Supplementary material for: Sex-Specific Differences in Clinical Outcomes After Transcatheter Aortic Valve Replacement: A Meta-Analysis of Reconstructed Individual Patient Survival Data
Source: Struct Heart. 2026 Jul 23;10(9):101100. doi: 10.1016/j.shj.2026.101100 (PMC13545726; doi:10.1016/j.shj.2026.101100)
Supplement: Supplementary file 1 [file mmc1.docx]

**Sex-specific Differences in Clinical Outcomes After Transcatheter Aortic Valve Replacement: A Meta-analysis of Reconstructed Individual Patient Survival Data**

Ali M. Abdelaziz; Mohamedhen Vall Nounou; Ahmad Al Othman; Mirvat Alasnag; Alaide Chieffo; J. Dawn Abbott; Abubaker Al-Allawee; Sofian Zreigh; Mohammed Benhammou; Muhiddin Dervis; Walid Guerguer; Ashraf Haidarah; Rafeek Elmezayen; Mohammad Alzu’bi; Serag Almzainy; Saber Khalid; Qasim Shawesh; Maram Abuajamieh; Muhammed Elhadi; Rodrigo Bagur; Mamas A. Mamas

**Supplementary Material**

Table of Contents

[Supplementary Table S1: PRISMA Checklist	4](#_Toc232962503)

[Supplementary Table S2: Literature Search strategy in each database	8](#_Toc232962504)

[Supplementary Table S3. Some potentially relevant studies for the meta-analysis and reasons for exclusion	9](#_Toc232962505)

[Supplementary Table S4: Overview summary of Included studies:	13](#_Toc232962506)

[Supplementary Table S5. Newcastle-Ottawa quality assessment scale for the included studies in the meta-analysis.	16](#_Toc232962507)

[Supplementary Table S6: Baseline clinical characteristics & comorbidities in patients across included studies:	21](#_Toc232962508)

[Supplementary Table S6: Baseline clinical characteristics & comorbidities in patients across included studies (Following):	24](#_Toc232962509)

[Supplementary Table S7: Frailty across included studies	27](#_Toc232962510)

[Supplementary Table S8: Valve generation across included studies	27](#_Toc232962511)

[Supplementary Table S9. Comparisons of The Original Curves to The Reconstructed Curves for All-cause Mortality	31](#_Toc232962512)

[Supplementary Table S10: Landmark analysis results for All-cause mortality	56](#_Toc232962513)

[Supplementary Table S11: Cox Model Results: All-cause Mortality	57](#_Toc232962514)

[Supplementary Table S12. Comparisons of The Original Curves to The Reconstructed Curves for Cardiovascular Mortality	59](#_Toc232962515)

[Supplementary Table S13: Landmark analysis results for Cardiovascular mortality	66](#_Toc232962516)

[Supplementary Table S14: Cox Model Results: Cardiovascular Mortality	67](#_Toc232962517)

[Supplementary Table S15. Comparisons of The Original Curves to The Reconstructed Curves for Stroke/TIA	69](#_Toc232962518)

[Supplementary Table S16: Landmark analysis results for Stroke/TIA:	74](#_Toc232962519)

[Supplementary Table S17: Cox Model Results: 000-Stroke	75](#_Toc232962520)

[Supplementary Table S18. Comparisons of The Original Curves to The Reconstructed Curves for Myocardial Infarction	77](#_Toc232962521)

[Supplementary Table S19: Landmark analysis results for Myocardial Infarction:	80](#_Toc232962522)

[Supplementary Table S20: Cox Model Results: 000-MI	81](#_Toc232962523)

[Supplementary Table S21. Comparisons of The Original Curves to The Reconstructed Curves for All-cause Rehospitalizations	83](#_Toc232962524)

[Supplementary Table S22: Landmark analysis results for All-cause Rehospitalizations:	88](#_Toc232962525)

[Supplementary Table S23: Cox Model Results: 000-Rehospitalization	89](#_Toc232962526)

[Supplementary Table S24: Comparison of reconstructed curves and original curves for HF hospitalizations	91](#_Toc232962527)

[Supplementary Table S25: Landmark analysis of HF hospitalizations	93](#_Toc232962528)

[Supplementary Table S26: Cox Model Results: 00-HF hospitalizations	94](#_Toc232962529)

[Supplementary Figure S1: PRISMA flowchart illustrating screening and selection of studies	96](#_Toc232962530)

[Supplementary Figure S2: All-cause mortality time-varying hazard ratio curve	96](#_Toc232962531)

[Supplementary Figure S3: Sensitivity Analysis of all-cause mortality restricted to studies with risk tables beneath KM plots	97](#_Toc232962532)

[Supplementary Figure S4: Sensitivity Analysis of all-cause mortality (pooling data from secondary analyses of RCTs only)	98](#_Toc232962533)

[Supplementary Figure S5: Subgroup Analysis of all-cause mortality by valve generation	99](#_Toc232962534)

[Supplementary Figure S9: Meta-regression of All-cause mortality using the average prevalence of comorbidities as covariates	101](#_Toc232962535)

[Supplementary Figure S10: Meta-regression of All-cause mortality using differences in comorbidities as covariates	103](#_Toc232962536)

[Supplementary Figure S6: All-cause mortality in East Asia	105](#_Toc232962537)

[Supplementary Figure S7: All-cause mortality in Europe	106](#_Toc232962538)

[Supplementary Figure S8: All-cause mortality in North America	107](#_Toc232962539)

[Supplementary Figure S11: Geographical region-based subgroup analysis of sex differences in CV mortality	108](#_Toc232962540)

[Supplementary Figure S12: time-varying HR for stroke / TIA :	109](#_Toc232962541)

[Supplementary Figure S13: Sensitivity analysis of stroke using studies with risk tables beneath KM plots	109](#_Toc232962542)

[Supplementary Figure S14: Geographically based sex differences in stroke	110](#_Toc232962543)

[Supplementary figure S15: Myocardial infarction	110](#_Toc232962544)

[Supplementary Figure S16: All-cause rehospitalizations	111](#_Toc232962545)

[Supplementary Figure S17: Heart failure rehospitalizations	111](#_Toc232962546)

[Supplementary Figure S18: Forest Plot of Permanent Pacemaker Implantation:	112](#_Toc232962547)

[Supplementary Figure S19: Forest Plot of Acute Kidney Injury:	113](#_Toc232962548)

[Supplementary Figure S20: Forest Plot of Cardiac Tamponade:	114](#_Toc232962549)

[Supplementary Figure S21: Forest Plot of VARC-defined Bleeding:	115](#_Toc232962550)

[Supplementary Figure S22: Forest Plot of VARC-defined Vascular Complications:	116](#_Toc232962551)

[Supplementary Figure S23: Sensitivity Analysis of Permanent Pacemaker Implantation:	117](#_Toc232962552)

[Supplementary Figure S24: Sensitivity Analysis of Acute Kidney Injury:	118](#_Toc232962553)

[Supplementary Figure S25: Sensitivity Analysis of Cardiac Tamponade:	119](#_Toc232962554)

[Supplementary Figure S26: Sensitivity Analysis of VARC-defined Bleeding:	120](#_Toc232962555)

[Supplementary Figure S27: Sensitivity Analysis of VARC-defined Vascular Complications:	121](#_Toc232962556)

[Supplementary Figure S28: Funnel plot of Permanent Pacemaker Implantation:	122](#_Toc232962557)

[Supplementary Figure S29: Funnel plot of Acute Kidney Injury:	123](#_Toc232962558)

[Supplementary Figure S30: Funnel plot of Cardiac Tamponade:	124](#_Toc232962559)

[Supplementary Figure S31: Funnel plot of VARC-defined Bleeding:	125](#_Toc232962560)

[Supplementary Figure S32: Funnel plot of VARC-defined Vascular Complications:	126](#_Toc232962561)

[Supplementary Figure S33: Meta-regression analysis of VARC-defined life-threatening bleeding:	127](#_Toc232962562)

[Supplementary Figure S34: Meta-regression analysis of VARC-defined major bleeding:	128](#_Toc232962563)

[Supplementary Figure S35: Meta-regression analysis of VARC-defined major vascular complications:	129](#_Toc232962564)

[Supplementary Figure S36: geographical-region-based subgroup analysis of bleeding and vascular complications	130](#_Toc232962565)

[Supplementary Figure S37: Subgroup analysis of newer versus older generation valves for Life-threatening Bleeding	133](#_Toc232962566)

[Supplementary Figure S38: Subgroup analysis of newer versus older generation valves for Major Bleeding	134](#_Toc232962567)

[Supplementary Figure S39: Subgroup analysis of newer versus older generation valves for Major Vascular Complications	135](#_Toc232962568)

[Supplementary Figure S40: meta-regression of 30-day all-cause mortality (ACM) and early procedural complications:	136](#_Toc232962569)

[References	137](#_Toc232962570)

# Supplementary Table S1: PRISMA Checklist

| **Section/Topic** | **Item #** | **Prisma 2020 Checklist item for manuscript** | **Location** |
| --- | --- | --- | --- |
| **TITLE** | | |  |
| Title | 1 | Identify the report as a systematic review. | Page 1 |
| **ABSTRACT** | | |  |
| Abstract | 2 | See the PRISMA 2020 for Abstracts checklist. | checked |
| **INTRODUCTION** | | |  |
| Rationale | 3 | Describe the rationale for the review in the context of existing knowledge. | Page 5 |
| Objectives | 4 | Provide an explicit statement of the objective(s) or question(s) the review addresses. | Page 5 |
| **METHODS** | | |  |
| Eligibility criteria | 5 | Specify the inclusion and exclusion criteria for the review and how studies were grouped for the syntheses. | Page 6 |
| Information sources | 6 | Specify all databases, registers, websites, organisations, reference lists and other sources searched or consulted to identify studies. Specify the date when each source was last searched or consulted. | Page 7 |
| Search strategy | 7 | Present the full search strategies for all databases, registers and websites, including any filters and limits used. | Table S2 |
| Selection process | 8 | Specify the methods used to decide whether a study met the inclusion criteria of the review, including how many reviewers screened each record and each report retrieved, whether they worked independently, and if applicable, details of automation tools used in the process. | Page 6 |
| Data collection process | 9 | Specify the methods used to collect data from reports, including how many reviewers collected data from each report, whether they worked independently, any processes for obtaining or confirming data from study investigators, and if applicable, details of automation tools used in the process. | Page 6 |
| Data items | 10a | List and define all outcomes for which data were sought. Specify whether all results that were compatible with each outcome domain in each study were sought (e.g. for all measures, time points, analyses), and if not, the methods used to decide which results to collect. | Page 6 |
|  | 10b | List and define all other variables for which data were sought (e.g. participant and intervention characteristics, funding sources). Describe any assumptions made about any missing or unclear information. | Page 7 |
| Study risk of bias assessment | 11 | Specify the methods used to assess risk of bias in the included studies, including details of the tool(s) used, how many reviewers assessed each study and whether they worked independently, and if applicable, details of automation tools used in the process. | Page 7 |
| Effect measures | 12 | Specify for each outcome the effect measure(s) (e.g. risk ratio, mean difference) used in the synthesis or presentation of results. | Page 7 |
| Synthesis methods | 13a | Describe the processes used to decide which studies were eligible for each synthesis (e.g. tabulating the study intervention characteristics and comparing against the planned groups for each synthesis (item #5)). | Page 7 |
|  | 13b | Describe any methods required to prepare the data for presentation or synthesis, such as handling of missing summary statistics, or data conversions. | Page 7 |
|  | 13c | Describe any methods used to tabulate or visually display results of individual studies and syntheses. | Page 7 |
|  | 13d | Describe any methods used to synthesize results and provide a rationale for the choice(s). If meta-analysis was performed, describe the model(s), method(s) to identify the presence and extent of statistical heterogeneity, and software package(s) used. | Page 7 |
|  | 13e | Describe any methods used to explore possible causes of heterogeneity among study results (e.g. subgroup analysis, meta-regression). | Page 7 |
|  | 13f | Describe any sensitivity analyses conducted to assess robustness of the synthesized results. | Page 7 |
| Reporting bias assessment | 14 | Describe any methods used to assess risk of bias due to missing results in a synthesis (arising from reporting biases). | Page 7 |
| Assessment | 15 | Describe any Certainty methods used to assess certainty (or confidence) in the body of evidence for an outcome. |  |
| **RESULTS** | | |  |
| Study selection | 16a | Describe the results of the search and selection process, from the number of records identified in the search to the number of studies included in the review, ideally using a flow diagram. | Page 8 |
|  | 16b | Cite studies that might appear to meet the inclusion criteria, but which were excluded, and explain why they were excluded. | Page 8 |
| Study characteristics | 17 | Cite each included study and present its characteristics. | Table S4 |
| Risk of bias in studies | 18 | Present assessments of risk of bias for each included study. | Table S5 |
| Results of individual studies | 19 | For all outcomes, present, for each study: (a) summary statistics for each group (where appropriate) and (b) an effect estimate and its precision (e.g. confidence/credible interval), ideally using structured tables or plots. | Page 9 /10 |
| Results of syntheses | 20a | For each synthesis, briefly summarise the characteristics and risk of bias among contributing studies. |  |
|  | 20b | Present results of all statistical syntheses conducted. If meta-analysis was done, present for each the summary estimate and its precision (e.g. confidence/credible interval) and measures of statistical heterogeneity. If comparing groups, describe the direction of the effect. | Page 9/10 |
|  | 20c | Present results of all investigations of possible causes of heterogeneity among study results. | Page 9/10 |
|  | 20d | Present results of all sensitivity analyses conducted to assess the robustness of the synthesized results. | Figures S12-S16 |
| Reporting biases | 21 | Present assessments of risk of bias due to missing results (arising from reporting biases) for each synthesis assessed. | Figures S17 – S21 |
| Certainty of evidence | 22 | Present assessments of certainty (or confidence) in the body of evidence for each outcome assessed. |  |
| **DISCUSSION** | | |  |
| Discussion | 23a | Provide a general interpretation of the results in the context of other evidence. | Page 13 |
|  | 23b | Discuss any limitations of the evidence included in the review. | Page 15/16 |
|  | 23c | Discuss any limitations of the review processes used. | Page 15/16 |
|  | 23d | Discuss implications of the results for practice, policy, and future research. | Page 15/16 |
| **OTHER INFORMATION** | | |  |
| Registration and protocol | 24a | Provide registration information for the review, including register name and registration number, or state that the review was not registered. | Page 6 |
|  | 24b | Indicate where the review protocol can be accessed, or state that a protocol was not prepared. | Page 6 |
|  | 24c | Describe and explain any amendments to information provided at registration or in the protocol. |  |
| Support | 25 | Describe sources of financial or non-financial support for the review, and the role of the funders or sponsors in the review. | Page 17 |
| Competing interests | 26 | Declare any competing interests of review authors. | Page 17 |
| Availability of data, code, and other materials | 27 | Report which of the following are publicly available and where they can be found: template data collection forms; data extracted from included studies; data used for all analyses; analytic code; any other materials used in the review. | Page 16 |

# Supplementary Table S2: Literature Search strategy in each database

| Database | Search strategy | Results |
| --- | --- | --- |
| **PubMed** | ((transcatheter aortic valve replacement) OR (TAVR) OR (transcatheter aortic valve implantation) OR (TAVI) OR (percutaneous aortic valve) OR (minimally invasive aortic valve replacement) OR (self-expand* aortic valve) OR (balloon-expand* valve) OR (aortic valv* intervention) OR (aortic valv* replacement)) AND ((sex) OR (gender dispari*) OR (sex differenc*) OR (gender differences) OR (sex disparities) OR (sex-related difference) OR (Sex variation) OR (gender variation)) AND ((aortic valve stenosis"[Mesh]) OR (aortic stenos*)) | 1568 |
| **Embase**  #1 | 'transcatheter aortic valve replacement':ti,ab,kw OR 'transcatheter aortic valve implantation':ti,ab,kw OR tavi:ti,ab,kw OR 'trans-catheter aortic valve replacement':ti,ab,kw OR 'trans-catheter aortic valve implantation':ti,ab,kw OR 'percutaneous aortic valve replacement':ti,ab,kw OR 'percutaneous aortic valve implantation':ti,ab,kw OR 'transcatheter valve replacement':ti,ab,kw OR 'transcatheter valve implantation':ti,ab,kw OR 'catheter-based aortic valve replacement':ti,ab,kw OR 'catheter-based aortic valve implantation':ti,ab,kw OR 'trans-femoral aortic valve implantation':ti,ab,kw OR 'transfemoral aortic valve implantation':ti,ab,kw OR 'transfemoral aortic valve replacement':ti,ab,kw OR 'trans-femoral aortic valve replacement':ti,ab,kw OR 'transapical aortic valve replacement':ti,ab,kw OR 'trans-apical aortic valve replacement':ti,ab,kw OR 'transapical aortic valve implantation':ti,ab,kw OR 'transsubclavian aortic valve replacement':ti,ab,kw OR 'trans-subclavian aortic valve replacement':ti,ab,kw OR 'transsubclavian aortic valve implantation':ti,ab,kw OR 'trans‐subclavian aortic valve implantation':ti,ab,kw |  |
| #2 | 'transcatheter aortic valve replacement'/exp OR 'transcatheter aortic valve implantation'/exp OR 'percutaneous aortic valve replacement'/exp OR 'aortic valve implantation' |  |
| #3 | #1 OR #2 |  |
| #4 | 'Sex difference'/exp OR 'gender difference'/exp OR 'sex disparity'/exp OR 'sex factors'/exp OR 'sex characteristic'/exp OR 'gender'/exp |  |
| #5 | #3 AND #4 | 1455 |
| **Scopus**  #1 | TITLE-ABS-KEY ("transcatheter aortic valve replacement" OR TAVR OR "transcatheter aortic valve implantation" OR TAVI OR "percutaneous aortic valve" OR "minimally invasive aortic valve replacement" OR "self-expanding aortic valve" OR "balloon-expandable valve" OR "aortic valve intervention" OR"aortic valve replacement") |  |
| #2 | TITLE-ABS-KEY(sex OR "gender disparities" OR "sex differences" OR "gender differences" OR "sex disparities" OR "sex-related difference" OR "sex variation" OR "gender variation") AND TITLE-ABS-KEY ("aortic valve stenosis" OR "aortic stenosis") |  |
| #3 | TITLE-ABS-KEY ("aortic valve stenosis" OR "aortic stenosis" ) |  |
| #4 | #1 AND #2 AND #3 | 1350 |
| **Web of Science**  All fields | ((transcatheter aortic valve replacement) OR (TAVR) OR (transcatheter aortic valve implantation) OR (TAVI) OR (percutaneous aortic valve) OR (minimally invasive aortic valve replacement) OR (self-expand* aortic valve) OR (balloon-expand* valve) OR (aortic valv* intervention) OR (aortic valv* replacement)) AND ((sex) OR (gender dispari*) OR (sex differenc*) OR (gender differences) OR (sex disparities) OR (sex-related difference) OR (Sex variation) OR (gender variation)) | 1983 |

# Supplementary Table S3. Some potentially relevant studies for the meta-analysis and reasons for exclusion

| **First author, year of publication** | **Citation** | **Reasons for exclusion** |
| --- | --- | --- |
| Kumar 2025 | Kumar M, Hu JR, Ali S, et al. Sex disparities in outcomes of transcatheter aortic valve implantation- a multi-year propensity-matched nationwide study. *Int J Cardiol*. 2025;418:132619. doi:10.1016/j.ijcard.2024.132619 | The study population has already been included in another study in our meta-analysis.  (duplicate data) |
| Thevathsasan T 2025 (TAVI COMIC trial) | Thevathasan T, Reitz F, Hornig C, et al. Sex Differences in Clinical and Patient-Reported Outcomes in Transcatheter Aortic Valve Implantation: The TAVI-COMIC Trial. *JACC Adv*. 2025;4(8):102012. doi:10.1016/j.jacadv.2025.102012 | Included patient-reported outcomes but lacked our pre-specified outcomes of interest and KM plots |
| Diaz-Arocutipa 2025 | Diaz-Arocutipa, C., Salguero, R., Puerto, E., Martín-Asenjo, R., & Vicent, L. (2025). Sex and race/ethnicity disparities on in-hospital outcomes in patients with severe aortic stenosis undergoing TAVR. *Expert Review of Cardiovascular Therapy*, 1–10. https://doi.org/10.1080/14779072.2025.2528916 | Some of the study population has already been included in another study in our meta-analysis.  (duplicate data) |
| Rivera F B 2024 | Rivera FB, Cha SW, Redula SC, et al. Sex differences in transcatheter aortic valve replacement outcomes among patients with bicuspid aortic stenosis. *Heart Lung*. 2024;67:144-151. doi:10.1016/j.hrtlng.2024.05.003 | Some of the study population have already been included in another study in our meta-analysis  (duplicate data) |
| Joshi V A 2025 | Joshi VA, Bucholz EM, Roll M, et al. Sex Differences in Stroke Frequency and Severity Following Transcatheter Aortic Valve Replacement. *J Am Heart Assoc*. 2025;14(15):e041943. doi:10.1161/JAHA.125.041943 | Some of the study population have already been included in another study in our meta-analysis.  (duplicate data) |
| Sevilla 2023 | Sevilla T, Ramos N, Carnero M, et al. Sex Differences in Clinical Outcomes after Aortic Valve Intervention for Isolated Severe Aortic Stenosis. *J Clin Med*. 2023;12(22):7025. Published 2023 Nov 10. doi:10.3390/jcm12227025 | Intervention is a mix of SAVR and TAVR. |
| See 2024 | See C, Wang Y, Yang Y, et al. Impact of sex on Transcatheter aortic valve replacement outcomes: Results of a single-center study. *Int J Cardiol*. 2024;398:131643. doi:10.1016/j.ijcard.2023.131643 | Some of the study population have already been included in another study in our meta-analysis.  (duplicate data) |
| Ravani 2024 | Ravani LV, Ribeiro HB, Calomeni P, et al. Clinical impact of sex differences and procedural setting in transcatheter aortic valve implantation. *Cardiovasc Revasc Med*. Published online September 24, 2024. doi:10.1016/j.carrev.2024.09.014 | The study population has already been included in another study in our meta-analysis.  (duplicate data) |
| Johnston 2024 | Johnston N, James SK, Lindhagen L, Ståhle E, Christersson C. Sex-specific aspects on prognosis after aortic valve replacement for aortic stenosis: a SWEDEHEART registry study. *Open Heart*. 2024;11(2):e002725. Published 2024 Jul 23. doi:10.1136/openhrt-2024-002725 | The study population has already been included in another study in our meta-analysis.  (duplicate data) |
| Novotny 2023 | Novotny S, Kort S, Tannous H, et al. Predictors of 1-Year Mortality in Men Versus Women Undergoing Transfemoral Transcatheter Aortic Valve Implantation. *Am J Cardiol*. 2023;186:1-4. doi:10.1016/j.amjcard.2022.10.018 | The study population has already been included in another study in our meta-analysis.  (duplicate data) |
| Liu 2023 | Liu Q, Wang Y, Li Y, et al. Gender differences in mortality following tanscatheter aortic valve replacement (TAVR): a single-centre retrospective analysis from China. *Chin Med J (Engl)*. 2023;136(20):2511-2513. doi:10.1097/CM9.0000000000002779 | Letter to the Editor |
| Erinne 2022 | Erinne I, Hiltner E, Bhasin V, et al. Sex Disparities in the Treatment of Aortic Stenosis and Related Outcomes: An Analysis of the National Inpatient Sample. *Cardiology*. 2022;147(5-6):557-565. doi:10.1159/000526255 | The study population has already been included in another study in our meta-analysis.  (duplicate data) |
| Sannino 2018 | Sannino A, Szerlip M, Harrington K, Schiattarella GG, Grayburn PA. Comparison of Baseline Characteristics and Outcomes in Men Versus Women With Aortic Stenosis Undergoing Transcatheter Aortic Valve Implantation. Am J Cardiol. 2018;121(7):844-849. doi:10.1016/j.amjcard.2017.12.020 | The study population has already been included in another study in our meta-analysis.  (duplicate data) |
| Holmes 2015 | Holmes DR Jr, Brennan JM, Rumsfeld JS, et al. Clinical outcomes at 1 year following transcatheter aortic valve replacement. JAMA. 2015;313(10):1019-1028. doi:10.1001/jama.2015.1474 | The study population has already been included in another study in our meta-analysis.  (duplicate data) |
| Amgai 2021 | Amgai B, Chakraborty S, Bandyopadhyay D, et al. Sex Differences in In-Hospital Outcomes of Transcatheter Aortic Valve Replacement. *Curr Probl Cardiol*. 2021;46(3):100694. doi:10.1016/j.cpcardiol.2020.100694 | The study population has already been included in another study in our meta-analysis.  (duplicate data) |
| Doshi 2017 | Doshi R, Shlofmitz E, Meraj P. Comparison of Outcomes and Complications of Transcatheter Aortic Valve Implantation in Women Versus Men (from the National Inpatient Sample). *Am J Cardiol*. 2018;121(1):73-77. doi:10.1016/j.amjcard.2017.09.015 | The study population has already been included in another study in our meta-analysis.  (duplicate data) |
| Zahid 2022 | Zahid S, Khan MZ, Ullah W, et al. Gender Differences in Age-Stratified Inhospital Outcomes After Transcatheter Aortic Valve Implantation (from the National Inpatient Sample 2012 to 2018). *Am J Cardiol*. 2022;167:83-92. doi:10.1016/j.amjcard.2021.11.038 | The study population has already been included in another study in our meta-analysis.  (duplicate data) |
| Stehli 2023 | Stehli J, Johnston R, Duffy SJ, et al. Waiting times of women vs. men undergoing transcatheter aortic valve implantation. *Eur Heart J Qual Care Clin Outcomes*. 2023;9(7):691-698. doi:10.1093/ehjqcco/qcac081 | Lacks our outcomes of interest |
| Vlastra 2019 | Vlastra W, Chandrasekhar J, García Del Blanco B, et al. Sex Differences in Transfemoral Transcatheter Aortic Valve Replacement. *J Am Coll Cardiol*. 2019;74(22):2758-2767. doi:10.1016/j.jacc.2019.09.015 | The study population has already been included in another study in our meta-analysis.  (duplicate data) |
| Williams 2014 | Williams M, Kodali SK, Hahn RT, et al. Sex-related differences in outcomes after transcatheter or surgical aortic valve replacement in patients with severe aortic stenosis: Insights from the PARTNER Trial (Placement of Aortic Transcatheter Valve). *J Am Coll Cardiol*. 2014;63(15):1522-1528. doi:10.1016/j.jacc.2014.01.036 | The study population has already been included in another study in our meta-analysis.  (duplicate data) |
| Okuno 2023 | Okuno T, Tomii D, Lanz J, et al. 5-Year Outcomes With Self-Expanding vs Balloon-Expandable Transcatheter Aortic Valve Replacement in Patients With Small Annuli. *JACC Cardiovasc Interv*. 2023;16(4):429-440. doi:10.1016/j.jcin.2022.11.032 | Data are not disaggregated by sex. |
| Prosperi-Porta 2023 | Prosperi-Porta G, Nguyen V, Willner N, et al. Association of Age and Sex With Use of Transcatheter Aortic Valve Replacement in France. *J Am Coll Cardiol*. 2023;82(20):1889-1902. doi:10.1016/j.jacc.2023.08.044 | No time-to-event outcomes data |
| Stamou 2012 | Stamou SC, Robich M, Wolf RE, Lovett A, Normand SL, Sellke FW. Effects of gender and ethnicity on outcomes after aortic valve replacement. *J Thorac Cardiovasc Surg*. 2012;144(2):486-492. doi:10.1016/j.jtcvs.2011.11.023 | Patients underwent SAVR, not TAVR |

# Supplementary Table S4: Overview summary of Included studies:

| **Study ID** | **Country** | **Study design** | **Study population by Sex** | | **Age** | | | | **STS-PROM score (%)** | | | | **Aortic valve Area (AVA) (cm2)** | | **Aortic valve gradient (mmHg)** | |
| --- | --- | --- | --- | --- | --- | --- | --- | --- | --- | --- | --- | --- | --- | --- | --- | --- |
|  |  |  | **Females** | **Males** | **Females** | | **Males** | | **Females** | | **Males** | | **Females** | **Males** | **Females** | **Males** |
|  |  |  |  |  | **Mean** | **SD** | **Mean** | **SD** | **Mean** | **SD** | **Mean** | **SD** |  |  |  |  |
| Sticchi A. et al 2025(1) | Multinational | Retrospective | 2162 | 1191 | 81.5 | 5.8 | 81.6 | 6.1 | 4.8 | 3 | 4.6 | 3.7 | 0.7 ±0.3 | 0.9 ±4.9 | NA | NA |
| Al-Azizi K. et al 2025(2) | USA | Retrospective | 1338 | 1678 | 80.03 | 9.35 | 79.37 | 8.9 | 5.77 | 3.86 | 4.47 | 3.26 | 0.67±0.22 | 0.72±0.19 | 44.83±9.28 | 43±9.65 |
| Matetic A. et al 2025(3) | Croatia | Retrospective | 140 | 180 | 81.73 | 4.24 | 80.77 | 6.2 | NA | NA | NA | NA | NA | NA | NA | NA |
| Adamo M. et al 2024 / EVOLUT | Multinational | Prospective | 338 | 249 | 82.64 | 5.28 | 81.41 | 6.13 | 3.56 | 1.88 | 3.1 | 1.68 | 0.65±0.22 | 0.73±0.21 | 44±13.40 | 45±14.17 |
| Adamo M. et al 2024 / SAPIEN(4) |  |  | 324 | 263 | 82.18 | 6.28 | 81.64 | 7.05 | 3.44 | 2.05 | 3.01 | 1.88 | 0.67±0.22 | 0.72±0.19 | 44.33±12.66 | 43.33±11.93 |
| Ahmad D. et al 2024(5) | USA | Retrospective | 152 | 152 | 80.83 | 8.61 | 81.33 | 7.48 | 6.97 | 5.31 | 5.77 | 4.27 | 0.67±0.22 | 0.7±0.15 | 45.83±10.10 | 43.33±8.98 |
| Chiu C. et al 2024(6) | Taiwan | Retrospective | 54 | 46 | 84.5 | 5.8 | 86 | 6.4 | 16.6 | 12.9 | 18.5 | 14.6 | 0.7 ± 0.2 | 0.8 ± 0.3 | 45.7 ± 19.2 | 47.5 ± 16.0 |
| Dismorr M. et al 2024(7) | Sweden | Retrospective | 4886 | 5589 | 81.4 | 6.9 | 79.9 | 6.9 | NA | NA | NA | NA | NA | NA | NA | NA |
| Gabani R. et al 2024(8) | Sapin | Retrospective | 6600 | 5653 | 82.1 | 5.7 | 80.2 | 6.9 | 7 | 7 | 6.2 | 6.7 | 0.64 ±0.2 | 0.71 ±0.2 | 49.4 ±15.2 | 45.0 ±14.0 |
| Iwata J. et al 2024(9) | Japan | Retrospective | 343 | 168 | 84.8 | 5.3 | 83.8 | 5.3 | 6.5 | 3.4 | 5.8 | 3.6 | 0.59 ±0.16 | 0.71 ±0.19 | 50.9 ±18.0 | 47.5 ±15.4 |
| Kim M. et al 2024(10) | Multinational | Prospective | 657 | 755 | 81 | 8 | 80 | 8 | 5.4 | 3.5 | 4.7 | 3.4 | 0.6±0.2 | 0.7±0.2 | 52.8±20.0 | 47.5±20.0 |
| Lodo V. et al 2024(11) | Italy | Retrospective | 204 | 204 | 82.1 | 4.8 | 82.5 | 5 | 3.89 | 3.5 | 3.78 | 3.2 | 0.67 ±0.38 | 0.69 ±0.36 | 50.6 ±13.6 | 52.3 ±10.5 |
| Tateishi K. et al 2024(12) | USA | Retrospective | 488 | 483 | 81.2 | 8.1 | 80.1 | 8.8 | 5.4 | 3.57 | 4.33 | 2.97 | NA | NA | NA | NA |
| Hoydahl M. et al 2024(13) | Norway | Retrospective | 422 | 445 | NA | NA | NA | NA | NA | NA | NA | NA | 0.6±0.15 | | NA | NA |
| Zhou C. et al 2023(14) | USA | Retrospective | 54802 | 54802 | 79.8 | 8.4 | 79.77 | 8.42 | NA | NA | NA | NA | NA | NA | NA | NA |
| Van Bergeijk K. et al 2023(15) | Netherlands | Post hoc of RCT | 466 | 512 | 81.24 | 5.46 | 79.39 | 6.7 | 3.6 | 2.62 | 3.03 | 2.29 | NA | NA | NA | NA |
| Leone P. et al 2023(16) | Multinational | Retrospective | 99 | 99 | 80.6 | 7.1 | 81 | 7.3 | 5.6 | 4.8 | 5.4 | 4 | 0.68 ± 0.40 | 0.67 ± 0.16 | 49.5 ± 15.3 | 46.3 ± 14.1 |
| Malvindi P. et al 2023(17) | Italy | Retrospective | 276 | 204 | 83 | 4.47 | 82 | 4.48 | NA | NA | NA | NA | NA | NA | NA | NA |
| Elbaz-Greener G. et al 2022(18) | USA | Retrospective | 70170 | 80477 | NA | NA | NA | NA | NA | NA | NA | NA | NA | NA | NA | NA |
| Alasnag M. et al 2022(19) | Gulf Countries | Retrospective | 347 | 448 | 74.11 | 9.1 | 74.91 | 8.75 | 5.3 | 4.35 | 4.59 | 4.1 | 0.7±0.2 | 0.7±0.2 | 47.1±14.4 | 45.1±14.1 |
| Medranda G. et al 2022(20) | USA | Retrospective | 389 | 480 | 78.6 | 9.4 | 78.1 | 11.5 | 5.7 | 3.8 | 4.2 | 4.6 | NA | NA | NA | NA |
| Patel P. et al 2022(21) | USA | Retrospective | 1041 | 1502 | 81.63 | 7.87 | 81.23 | 7.64 | 6.07 | 4.01 | 5.17 | 3.71 | 0.8±0.15 | 0.9±0.15 | 45±11.14 | 42.33±9.65 |
| Wohrle J. et al 2022(22) | Germany | post hoc of RCT | 813 | 831 | 81.12 | 6.16 | 80.04 | 6.93 | 4.68 | 3.63 | 3.64 | 2.96 | NA | NA | NA | NA |
| He J. et al 2022(23) | China | Retrospective | 225 | 285 | 72 | 5.8 | 72.1 | 8 | 5.6 | 3.8 | 4.9 | 4.2 | NA | NA | 67.8±21 | 57.6±18 |
| Kuneman J. et al 2022(24) | Netherlands | Retrospective | 219 | 240 | 81 | 7 | 80 | 7 | NA | NA | NA | NA | NA | NA | 42±17 | 41±15 |
| Chiam P. et al 2021(25) | East Asia | Retrospective | 398 | 475 | 80.8 | 6.8 | 78.9 | 7.5 | NA | NA | NA | NA | 0.6 | 0.7 | 50.8 | 51 |
| Deharo P. et al 2021(26) | France | Retrospective | 12336 | 12336 | 83 | 6.6 | 82.9 | 6.6 | NA | NA | NA | NA | NA | NA | NA | NA |
| Denegri A. et al 2021(27) | Italy | Prospective | 689 | 689 | 83 | 6 | 81 | 6 | 7.8 | 7.1 | 7.2 | 7.5 | NA | NA | 52.4±15.3 | 47.3±12.8 |
| Gonçalves M. et al 2021(28) | USA | Prospective | 316 | 276 | 84.3 | 5.3 | 81 | 7.5 | NA | NA | NA | NA | NA | NA | NA | NA |
| Rodriguez-Gabella T. et al 2021(29) | Spain | Retrospective | 1565 | 1565 | 81.75 | 6.04 | 81.4 | 6.94 | 5.18 | 3.25 | 5.1 | 4.18 | 0.63 ± 0.16 | 0.69 ± 0.17 | 47.41 ± 15.06 | 46.49 ± 14.56 |
| Shishido K. et al 2021(30) | Japan | Prospective | 1,793 | 795 | 84.7 | 5 | 83.6 | 5.5 | 8 | 6 | 9 | 9 | 0.6±0.2 | 0.7±0.2 | 52±18.8 | 47.4±16.4 |
| Simard T. et al 2021(31) | USA | Retrospective | 16900 | 16900 | 79.3 | 9.3 | 79.3 | 9.1 | NA | NA | NA | NA | NA | NA | NA | NA |
| Useini D. et al 2021(32) | Germany | Retrospective | 58 | 45 | 83.2 | 4.5 | 82 | 4.7 | 3.6 | 1.8 | 3.7 | 1.6 | NA | NA | NA | NA |
| Chang H. et al 2020(33) | Taiwan | Retrospective | 125 | 96 | 81.2 | 8 | 81.7 | 8.9 | NA | NA | NA | NA | 0.2 | 0.2 | 19.6 | 16.9 |
| Chen S. et al 2020(34) | Taiwan | Retrospective | 50 | 50 | 79.46 | 9.95 | 80.96 | 8.79 | NA | NA | NA | NA | 0.64 ± 0.21 | 0.76 ± 0.23 | 49.1 ± 22.5 | 43.0 ± 17.6 |
| Stehli J. et al 2020(35) | Australia | Prospective | 328 | 355 | 84.2 | 5.2 | 83.2 | 6.9 | 5.2 | 3.1 | 4.6 | 3.5 | 0.7±0.1 | 0.8±0.2 | 50.9±15.2 | 46.2±13.4 |
| Tarantini G. et al 2020(36) | Multinational | Prospective | 834 | 860 | 82.7 | 6.29 | 80.8 | 6.91 | NA | NA | NA | NA | NA | NA | NA | NA |
| Wilczek K. et al 2020(37) | Poland | Prospective | 132 | 143 | 79.33 | 7.5 | 77 | 7.49 | 6.27 | 4.32 | 4.77 | 2.87 | 0.63±0.21 | 0.68±0.19 | 48.33±14.24 | 44.67±15.73 |
| Van Mieghem N. et al 2020(38) | Netherlands | post hoc of RCT | 366 | 498 | 79.9 | 6 | 79.9 | 6.4 | 4.6 | 1.5 | 4.2 | 1.5 | NA | NA | NA | NA |
| Chikata Y. et al 2020(39) | Japan | Retrospective | 746 | 342 | 84.38 | 5.15 | 83.27 | 6.28 | 6.21 | 3.16 | 5.43 | 3.33 | 0.65 ±0.17 | 0.70 ±0.17 | 51.95 ±19.85 | 49.40 ±17.29 |
| Linke A. et al 2019 (40) | Germany | Retrospective | 1026 | 792 | 81.15 | 40.48 | 79.16 | 41.33 | 8.42 | 5.05 | 6.86 | 4.7 | 0.63±0.19 | 0.73±0.18 | 45.57±15.39 | 40.61±13.26 |
| Pighi M. et al 2019(41) | Multinational | Prospective | 340 | 419 | 84.2 | 5.3 | 82.8 | 5.8 | 6.5 | 3.8 | 5.8 | 4.4 | 0.7±0.2 | 0.8±0.2 | 50.1±18.2 | 44.3±13.5 |
| Wang T. et al 2019(42) | USA | Prospective | 144 | 154 | 81 | 8 | 79 | 9 | NA | NA | NA | NA | 0.7 | NA | NA | NA |
| Singh A. et al 2019(43) | United Kingdom | Prospective | 132 | 143 | NA | NA | NA | NA | NA | NA | NA | NA | NA | NA | NA | NA |
| Kilic A. et al 2019(44) | USA | Retrospective | 518 | 518 | 81.8 | 7 | 81.8 | 7.8 | 8.3 | 5 | 7.7 | 4.4 | 0.6±0.2 | 0.7±0.2 | 51.2±14.7 | 45.6±13.6 |
| Kaier K. et al 2018 (45) | Germany | Retrospective | 19344 | 16126 | NA | NA | NA | NA | NA | NA | NA | NA | NA | NA | NA | NA |
| Szerlip M. et al 2018(46) | USA | post hoc of RCT | 657 | 1004 | 82.5 | 7.2 | 82 | 7.1 | 6.8 | 3 | 6.3 | 2.8 | 0.6±0.2 | 0.7±0.2 | 47.8±13.8 | 44.6±12.8 |
| Yousif N. et al 2018(47) | Switzerland | Retrospective | 281 | 265 | 83.3 | 6.95 | 80.7 | 6.91 | 6.1 | NA | 7.1 | NA | NA | NA | NA | NA |
| Zadok O. et al 2018 2008-2012(48) | Israel | Retrospective | 523 | 372 | 83.1 | 5.7 | 82.1 | 6.7 | 4.93 | 2.6 | 4.37 | 2.6 | 0.62±0.19 | 0.7±0.15 | 48.33±14.12 | 43.67±14.88 |
| Zadok O. et al 2018 2013-2016 |  |  | 847 | 787 | 82 | 6.3 | 82 | 6.9 | 3.97 | 2.08 | 3.7 | 2.15 | 0.68±0.2 | 0.72±0.2 | 46.67±14.85 | 42.67±13.37 |
| Bagienski M. et al 2017(49) | Poland | Prospective | 61 | 40 | 81.33 | 4.56 | 78.33 | 7.69 | 13.67 | 15.19 | 14 | 12.3 | 0.63±0.23 | 0.73±0.23 | 55.17±20.12 | 48.67±10.77 |
| Bauer T. et al 2017(50) | Germany | Retrospective | 8456 | 7160 | 82.67 | 5.19 | 81 | 5.93 | 5.93 | 3.19 | 4.77 | 3.04 | 0.63±0.22 | 0.73±0.22 | 45.33±15.57 | 40±14.83 |
| Czarnecki A. et al 2017(51) | Canada | Retrospective | 453 | 546 | 84.33 | 5.95 | 82.33 | 7.43 | 7 | 5 | 8 | 7 | NA | NA | NA | NA |
| Katz M. et al 2017(52) | Brazil | Retrospective | 418 | 401 | 82.4 | 7 | 80.6 | 7.5 | 15.4 | 13 | 11.4 | 9.5 | 0.6±0.2 | 0.7±0.2 | 52.2±15.7 | 46.3±15.7 |
| Chandrasekhar J. et al 2016(53) | USA | Prospective | 11808 | 11844 | 82.28 | 8.52 | 81.67 | 8.63 | 9 | 6 | 8 | 6 | NA | NA | NA | NA |
| Forrest J. et al 2016 (54) | USA | Posthoc of RCT | 1708 | 1979 | 84 | 7.6 | 82.7 | 7.9 | 9.6 | 4.9 | 8.3 | 4.6 | NA | NA | NA | NA |
| Kodali S. et al 2016(55) | Multinational | Posthoc of RCT | 1220 | 1339 | 84.9 | 6.9 | 84.1 | 7.3 | 11.9 | 4.2 | 11.1 | 4 | 0.6±0.2 | 0.7±0.2 | 46.1±14.7 | 42±13.7 |
| Gaglia M. et al 2016(56) | USA | Retrospective | 383 | 372 | 83.5 | 7.7 | 82.4 | 7.7 | 9.8 | 4.6 | 8 | 4.4 | 0.63±0.1 | 0.7±0.1 | 50.1±13.3 | 44.9±11.5 |
| Gilard M. et al 2016(57) | Multinational | Prospective | 2274 | 2290 | NA | NA | NA | NA | NA | NA | NA | NA | 0.7±0.3 | | 49.1±16.3 | |
| Codner P. et al 2015(58) | Israel | Prospective | 203 | 157 | NA | NA | NA | NA | NA | NA | NA | NA | 0.6±0.2 | | 49.4±16.8 | |
| Al-Lamee R. et al 2014(59) | UK | Retrospective | 756 | 871 | 82.6 | 6.8 | 80.8 | 7.6 | NA | NA | NA | NA | 0.6±0.2 | 0.7±0.4 | NA | NA |
| Conrotto F. et al 2014(60) | Multinational | Retrospective | 464 | 372 | 82.5 | 6.1 | 80.3 | 6.9 | NA | NA | NA | NA | 0.7±0.2 | 0.8±0.2 | 54.3±20.1 | 47.5±16.1 |
| Madershahian N. et al 2014(61) | Germany | Retrospective | 29 | 26 | 84 | 5.6 | 80.6 | 7.1 | 16.8 | 9.7 | 18.6 | 8.6 | NA | NA | NA | NA |
| Onorati F. et al 2014(62) | Multinational | Prospective | 428 | 297 | 82.4 | 6.3 | 81.1 | 6.6 | NA | NA | NA | NA | 0.6±0.2 | 0.7±0.3 | 51.8±14.7 | 48.4±15.3 |
| Sherif M. et al 2014(63) | Germany | Prospective | 827 | 605 | 82.8 | 5.8 | 80.3 | 6.4 | NA | NA | NA | NA | 0.7±0.4 | 0.7±0.4 | 52.1±20.8 | 45.5±15.8 |
| Erez A. et al 2014(64) | Israel | Prospective | 127 | 97 | 82 | 6 | 81 | 8 | NA | NA | NA | NA | 0.7±0.2 | 0.7±0.2 | 51±17 | 44±15 |
| Buja P. et al 2013(65) | Italy | Prospective | 368 | 291 | 82 | 5 | 80 | 7 | NA | NA | NA | NA | NA | NA | 55±18 | 47±14 |
| D’Ascenzo F. et al 2013(66) | Italy | Prospective | 216 | 161 | 81.65 | 5.32 | 82.9 | 5.45 | 5.59 | 3.13 | 7.64 | 6.2 | 0.6±0.2 | 0.6±0.2 | 56.4±18.2 | 48.6±13.9 |
| Ferrante G. et al 2013(67) | Italy | Prospective | 348 | 308 | 83.47 | 4.91 | 82.63 | 5.81 | NA | NA | NA | NA | 0.72±0.33 | 0.66±0.25 | 50.67±13.4 | 50±14.15 |
| Hayashida K. et al 2012(68) | France | Prospective | 131 | 129 | 83.8 | 5.9 | 82.4 | 6.5 | NA | NA | NA | NA | 0.6± 0.15 | | 47.6±18.1 | |
| Humphries K. et al 2012(69) | Canada | Prospective | 329 | 312 | 82 | 8.19 | 81.33 | 7.45 | 7.57 | 3.87 | 7.73 | 4.54 | 0.6±0.15 | 0.7±0.15 | 42±15.64 | 40.33±15.64 |
| Stangl V. et al 2012(70) | Germany | Prospective | 58 | 42 | 80 | 8 | 77 | 9 | NA | NA | NA | NA | 0.7±0.3 | 0.8±0.2 | 45.4±20 | 43±15.9 |
| Buchanan G. et al 2011(71) | Italy | Prospective | 146 | 159 | 80.1 | 6.8 | 78.8 | 7.8 | 9.1 | 7.9 | 8.2 | 7.8 | NA | NA | NA | NA |

# Supplementary Table S5. Newcastle-Ottawa quality assessment scale for the included studies in the meta-analysis.

|  | | **Selection** | | | | | | | |  | | **Outcome** | | | | | |  |
| --- | --- | --- | --- | --- | --- | --- | --- | --- | --- | --- | --- | --- | --- | --- | --- | --- | --- | --- |
| First author, Year | | Represent-ativeness | | Selection of comparison cohort | | Ascertainment | | Endpoint not present at start | | **Comparability of both groups** | | Assessment of outcome | | Follow up duration | | Adequacy follow up | | **Total score** |
| Matetic A. et al 2025 | * | | * | | * | | * | | * | | * | | * | | * | | 8 | |
| Sticchi A. et al 2025 | * | | * | | * | | * | | ** | | * | | * | | * | | 9 | |
| Al-Azizi K. et al 2025 | * | | * | | * | | * | | ** | | * | | * | | * | | 9 | |
| Adamo M. et al 2024 | * | | * | | * | | * | | ** | | * | | * | | * | | 9 | |
| Ahmad D. et al 2024 | * | | * | | * | | * | | ** | | * | | * | | * | | 9 | |
| Chiu C. et al 2024 | * | | * | | * | | * | | ** | | * | | * | | * | | 9 | |
| Dismorr M. et al 2024 | * | | * | | * | | * | | * | | * | | * | | * | | 8 | |
| Gabani R. et al 2024 | * | | * | | * | | * | | ** | | * | | * | | * | | 9 | |
| Iwata J. et al 2024 | * | | * | | * | | * | | ** | | * | | * | | * | | 9 | |
| Kim M. et al 2024 | * | | * | | * | | * | | ** | | * | | * | | * | | 9 | |
| Lodo V. et al 2024 | * | | * | | * | | * | | ** | | * | | * | | * | | 9 | |
| Tateishi K. et al 2024 | * | | * | | * | | * | | ** | | * | | * | | * | | 9 | |
| Hoydahl M. et al 2024 | * | | * | | * | | * | |  | | * | | * | | * | | 7 | |
| Zhou C. et al 2023 | * | | * | | * | | * | | ** | | * | | * | | * | | 9 | |
| Van Bergeijk K. et al 2023 | * | | * | | * | | * | | ** | | * | | * | | * | | 9 | |
| Leone P. et al 2023 | * | | * | | * | | * | | ** | | * | | * | | * | | 9 | |
| Malvindi P. et al 2023 | * | | * | | * | | * | | * | | * | | * | | * | | 8 | |
| Elbaz-Greener G. et al 2022 | * | | * | | * | | * | | * | | * | |  | | * | | 7 | |
| Alasnag M. et al 2022 | * | | * | | * | | * | | * | | * | | * | | * | | 8 | |
| Medranda G. et al 2022 | * | | * | | * | | * | | ** | | * | | * | | * | | 9 | |
| Patel P. et al 2022 | * | | * | |  | | * | | ** | | * | |  | | * | | 7 | |
| Wohrle J. et al 2022 | * | | * | | * | | * | | * | | * | | * | | * | | 8 | |
| He J. et al 2022 | * | | * | | * | | * | | ** | | * | | * | | * | | 9 | |
| Kuneman J. et al 2022 | * | | * | | * | | * | | ** | | * | | * | | * | | 9 | |
| Chiam P. et al 2021 | * | | * | | * | | * | | * | | * | |  | | * | | 7 | |
| Deharo P. et al 2021 | * | | * | | * | | * | | ** | | * | | * | | * | | 9 | |
| Denegri A. et al 2021 | * | | * | | * | | * | | ** | | * | | * | | * | | 9 | |
| Gonçalves M. et al 2021 | * | | * | | * | | * | | ** | | * | | * | | * | | 9 | |
| Rodriguez-Gabella T. et al 2021 | * | | * | | * | | * | | * | | * | | * | | * | | 8 | |
| Shishido K. et al 2021 | * | | * | | * | | * | | ** | | * | | * | | * | | 9 | |
| Simard T. et al 2021 | * | | * | | * | | * | | ** | | * | |  | | * | | 8 | |
| Useini D. et al 2021 | * | | * | | * | | * | | ** | | * | | * | | * | | 9 | |
| Chang H. et al 2020 | * | | * | | * | | * | | ** | | * | | * | | * | | 9 | |
| Chen S. et al 2020 | * | | * | | * | | * | | * | | * | | * | | * | | 8 | |
| Stehli J. et al 2020 | * | | * | | * | | * | | ** | | * | | * | | * | | 9 | |
| Tarantini G. et al 2020 | * | | * | | * | | * | | * | | * | | * | | * | | 8 | |
| Wilczek K. et al 2020 | * | | * | | * | | * | | * | | * | | * | | * | | 8 | |
| Chikata Y. et al 2020 | * | | * | | * | | * | | * | | * | | * | | * | | 8 | |
| Linke A. et al 2019 | * | | * | | * | | * | | ** | | * | | * | | * | | 9 | |
| Pighi M. et al 2019 | * | | * | | * | | * | | * | | * | | * | | * | | 8 | |
| Wang T. et al 2019 | * | | * | | * | | * | | * | | * | | * | | * | | 8 | |
| Singh A. et al 2019 | * | | * | | * | | * | | * | | * | |  | | * | | 7 | |
| Kilic A. et al 2019 | * | | * | | * | | * | | ** | | * | | * | | * | | 9 | |
| Kaier K. et al 2018 | * | | * | | * | | * | | * | | * | |  | | * | | 7 | |
| Szerlip M. et al 2018 | * | | * | | * | | * | | ** | | * | | * | | * | | 9 | |
| Yousif N. et al 2018 | * | | * | | * | | * | | * | | * | | * | | * | | 7 | |
| Zadok O. et al 2018 | * | | * | | * | | * | | * | | * | | * | |  | | 7 | |
| Bagienski M. et al 2017 | * | | * | | * | | * | | * | | * | | * | | * | | 8 | |
| Bauer T. et al 2017 | * | | * | | * | | * | | ** | | * | | * | | * | | 9 | |
| Czarnecki A. et al 2017 | * | | * | | * | | * | | ** | | * | | * | | * | | 9 | |
| Katz M. et al 2017 | * | | * | | * | | * | | * | | * | | * | | * | | 8 | |
| Chandrasekhar J. et al 2016 | * | | * | | * | | * | | * | | * | | * | | * | | 8 | |
| Forrest J. et al 2016 | * | | * | | * | | * | | * | | * | | * | | * | | 8 | |
| Kodali S. et al 2016 | * | | * | | * | | * | | ** | | * | | * | | * | | 9 | |
| Gaglia M. et al 2016 | * | | * | | * | | * | | * | | * | | * | | * | | 8 | |
| Gilard M. et al 2016 | * | | * | | * | | * | | ** | | * | | * | | * | | 9 | |
| Codner P. et al 2015 | * | | * | | * | | * | | * | | * | | * | |  | | 7 | |
| Al-Lamee R. et al 2014 | * | | * | | * | | * | | * | | * | | * | |  | | 7 | |
| Conrotto F. et al 2014 | * | | * | | * | | * | | * | | * | | * | |  | | 7 | |
| Madershahian N. et al 2014 | * | | * | | * | | * | |  | | * | | * | |  | | 6 | |
| Onorati F. et al 2014 | * | | * | | * | | * | | * | | * | | * | |  | | 7 | |
| Sherif M. et al 2014 | * | | * | | * | | * | | * | | * | | * | | * | | 8 | |
| Erez A. et al 2014 | * | | * | | * | | * | | ** | | * | | * | | * | | 9 | |
| Buja P. et al 2013 | * | | * | | * | | * | | * | | * | | * | | * | | 8 | |
| D’Ascenzo F. et al 2013 | * | | * | | * | | * | | ** | | * | | * | | * | | 9 | |
| Ferrante G. et al 2013 | * | | * | | * | | * | | ** | | * | | * | | * | | 8 | |
| Hayashida K. et al 2012 | * | | * | | * | | * | | * | | * | | * | | * | | 8 | |
| Humphries K. et al 2012 | * | | * | | * | | * | | ** | | * | | * | | * | | 9 | |
| Stangl V. et al 2012 | * | | * | | * | | * | | * | | * | | * | | * | | 8 | |
| Buchanan G. et al 2011 | * | | * | | * | | * | | * | | * | | * | | * | | 8 | |
| Notes. The Newcastle-Ottawa scale uses a star system (0 to 9) to evaluate included studies on 3 domains, selection, comparability, and outcomes. Star (*) item presents Maximum 1 star (*) for selection and outcome components and 2 stars (**) for comparability components. Higher scores represent higher study quality | | | | | | | | | | | | | | | | | | |

# Supplementary Table S6: Baseline clinical characteristics & comorbidities in patients across included studies:

| **Study ID** | **LVEF (%)** | | | | **HTN*** | | | | **DM** | | | | **Prior stroke/TIA** | | | |
| --- | --- | --- | --- | --- | --- | --- | --- | --- | --- | --- | --- | --- | --- | --- | --- | --- |
|  | **Female** | | **Male** | | **Female** | | **Male** | | **Female** | | **Male** | | **Female** | | **Male** | |
|  | **Mean** | **Sd** | **Mean** | **Sd** | **Event n** | **Total** | **Event n** | **Total** | **Event n** | **Total** | **Event n** | **Total** | **Event n** | **Total** | **Event n** | **Total** |
| Sticchi A. et al 2025 | 56.2 | 11 | 56.5 | 11.2 | 718 | 844 | 726 | 844 | 254 | 844 | 258 | 844 | 98 | 844 | 101 | 844 |
| Al-Azizi K. et al 2025 | 63.17 | 6.31 | 58.33 | 11.13 | 1197 | 1338 | 1496 | 1678 | 544 | 1338 | 682 | 1678 | - | - | - | - |
| Matetic A. et al 2025 | - | - | - | - | 96 | 140 | 132 | 180 | 38 | 140 | 60 | 180 | - | - | - | - |
| Adamo M. et al 2024 / EVOLUT | 59.67 | 6.71 | 60 | 7.45 | 290 | 338 | 213 | 249 | 90 | 338 | 78 | 249 | 28 | 338 | 29 | 249 |
| Adamo M. et al 2024 / SAPIEN | 60 | 7.41 | 59.58 | 8.33 | 277 | 324 | 224 | 263 | 79 | 324 | 85 | 263 | 24 | 324 | 40 | 263 |
| Ahmad D. et al 2024 | 58.17 | 7.48 | 53 | 11.98 | 145 | 844 | 145 | 844 | 75 | 152 | 77 | 152 | 18 | 152 | 28 | 152 |
| Chiu C. et al 2024 | 59.6 | 14 | 54.7 | 17.2 | 49 | 54 | 33 | 46 | 24 | 54 | 20 | 46 | 5 | 54 | 6 | 46 |
| Dismorr M. et al 2024 | - | - | - | - | 4,371 | 4886 | 5002 | 5589 | 1238 | 4886 | 1898 | 5589 | 1009 | 4886 | 1380 | 5589 |
| Gabani R. et al 2024 | 59.6 | 11.9 | 54.3 | 13.7 | 5380 | 6600 | 4487 | 5653 | 2103 | 6600 | 2129 | 5653 | 600 | 6600 | 638 | 5653 |
| Iwata J. et al 2024 | 62.6 | 11.3 | 62.5 | 11.7 | 270 | 343 | 137 | 168 | 82 | 343 | 52 | 168 | 17 | 343 | 16 | 168 |
| Kim M. et al 2024 | 54.6 | 13.6 | 59.9 | 11.3 | 574 | 657 | 642 | 755 | 253 | 657 | 337 | 755 | 71 | 657 | 89 | 755 |
| Lodo V. et al 2024 | 59.9 | 8.6 | 59.7 | 8.8 | 186 | 204 | 183 | 204 | 59 | 204 | 55 | 204 | 7 | 204 | 3 | 204 |
| Tateishi K. et al 2024 | 58.1 | 12.1 | 52.1 | 13.3 | 450 | 488 | 438 | 483 | 170 | 488 | 166 | 483 | 22 | 488 | 29 | 483 |
| Hoydahl M. et al 2024 | -- | -- | -- | -- | -- | -- | -- | -- | -- | -- | -- | -- | -- | -- | -- | -- |
| Zhou C. et al 2023 |  |  |  |  |  |  |  |  | 12578 | 54802 | 12589 | 54802 | 6219 | 54802 | 6342 | 54802 |
| Van Bergeijk K. et al 2023 | - | - | - | - | 355 | 466 | 363 | 512 | 102 | 466 | 150 | 512 | 52 | 466 | 76 | 512 |
| Leone P. et al 2023 | -- | -- | -- | -- | 80 | 99 | 78 | 99 | 33 | 99 | 30 | 99 | -- | -- | -- | -- |
| Malvindi P. et al 2023 | 60 | 7.45 | 54 | 11.95 | 226 | 276 | 166 | 204 | 66 | 276 | 61 | 204 | 46 | 276 | 30 | 204 |
| Elbaz-Geener G. et al 2022 | - | - | - | - | 38243 | 70170 | 40480 | 80477 | 23016 | 70170 | 29857 | 80477 | 3649 | 70170 | 4024 | 80477 |
| Alasnag M. et al 2022 | 56.2 | 10.52 | 50.04 | 13.54 | 299 | 347 | 365 | 448 | 219 | 347 | 271 | 448 | 21 | 347 | 41 | 448 |
| Medranda G. et al 2022 | - | - | - | - | 353 | 389 | 419 | 480 | 131 | 389 | 186 | 480 | - | - | - | - |
| Patel P. et al 2022 | 63 | 8.17 | 58.33 | 11.13 | - | - | - | - | 270 | 1041 | 435 | 1502 | 108 | 1041 | 190 | 1502 |
| Wohrle J. et al 2022 | 59.63 | 10 | 55.92 | 11.68 | 721 | 813 | 696 | 831 | 201 | 813 | 270 | 831 | 34 | 813 | 52 | 831 |
| He J. et al 2022 | 57.9 | 15.2 | 52.7 | 15.7 | 82 | 225 | 99 | 285 | 38 | 225 | 47 | 285 | 34 | 225 | 56 | 285 |
| Kuneman J. et al 2022 | 52 | 11 | 50 | 10 | 157 | 219 | 175 | 240 | 58 | 219 | 80 | 240 | 43 | 219 | 44 | 240 |
| Chiam P. et al 2021 | 59 | 13 | 55 | 14 | 358 | 475 | 305 | 398 | 149 | 475 | 125 | 398 | - | - | - | - |
| Deharo P. et al 2021 | - | - | - | - | 9838 | 12336 | 9839 | 12336 | 3549 | 12336 | 3522 | 12336 | 666 | 12336 | 646 | 12336 |
| Denegri A. et al 2021 | 54.3 | 11.2 | 49.7 | 12.7 | 1807 | 2138 | 1351 | 1662 | 588 | 2123 | 546 | 1656 | 76 | 1513 | 95 | 1227 |
| Gonçalves M. et al 2021 | 56 | 12 | 51 | 15 | 277 | 316 | 231 | 276 | 94 | 316 | 96 | 276 | 33 | 216 | 38 | 276 |
| Rodriguez-Gabella T. et al 2021 | 55.04 | 10.98 | 54.34 | 11.71 | NA | NA | NA | NA | 441 | 1565 | 464 | 1565 | 207 | 1565 | 182 | 1565 |
| Shishido K. et al 2021 | 60 | 12 | 57 | 13 | 1,367 | 1,793 | 623 | 795 | 334 | 1,793 | 221 | 795 | 119 | 1,793 | 182 | 795 |
| Simard T. et al 2021 | NA | NA | NA | NA | NA | NA | NA | NA | 6798 | 16900 | 6753 | 16900 | 2213 | 16900 | 2167 | 16900 |
| Useini D. et al 2021 | 57.3 | 8 | 54.5 | 9.5 | 50 | 58 | 36 | 45 | 18 | 58 | 11 | 45 | 7 | 58 | 7 | 45 |
| Chang H. et al 2020 | 56.5 | 9.4 | 53.4 | 10.9 | 89 | 125 | 72 | 96 | 43 | 125 | 40 | 96 | NA | NA | NA | NA |
| Chen S. et al 2020 | 55.2 | 10.9 | 54.7 | 11.5 | 34 | 50 | 38 | 50 | 17 | 50 | 19 | 50 | 7 | 50 | 13 | 50 |
| Stehli J. et al 2020 | 60.9 | 9.6 | 55.7 | 12.2 | 240 | 328 | 236 | 355 | 83 | 328 | 90 | 355 | NA | NA | NA | NA |
| Tarantini G. et al 2020 | 57.9 | 12.3 | 51.9 | 14.67 | 686 | 834 | 695 | 860 | 227 | 834 | 260 | 860 | NA | NA | NA | NA |
| Wilczek K. et al 2020 | 50.67 | 7.5 | 44 | 16.49 | 111 | 131 | 109 | 141 | 65 | 131 | 62 | 142 | 9 | 125 | 17 | 134 |
| Van Mieghem N. et al 2020 | NA | NA | NA | NA | NA | NA | NA | NA | 113 | 366 | 182 | 498 | 15 | 366 | 42 | 498 |
| Chikata Y. et al 2020 | 61.9 | 9.97 | 57.96 | 12.03 | 585 | 748 | 256 | 342 | 158 | 748 | 104 | 342 | 75 | 748 | 49 | 342 |
| Linke A. et al 2019 | 58.39 | 12.23 | 50.88 | 16.1 | 958 | 1016 | 729 | 787 | NA | NA | NA | NA | 87 | 1016 | 93 | 787 |
| Pighi M. et al 2019 | 58.8 | 10.9 | 52.9 | 13.3 | NA | NA | NA | NA | 66 | 340 | 134 | 419 | 62 | 340 | 79 | 419 |
| Wang T. et al 2019 | NA | NA | NA | NA | NA | NA | NA | NA | 49 | 144 | 67 | 154 | 24 | 144 | 22 | 154 |
| Singh A. et al 2019 |  |  |  |  |  |  |  |  |  |  |  |  |  |  |  |  |
| Kilic A. et al 2019 | 56.9 | 11.7 | 49.6 | 14.4 | 458 | 518 | 468 | 518 | 196 | 518 | 235 | 518 | 166 | 518 | 159 | 518 |
| Kaier K. et al 2018 | NA | NA | NA | NA | 12164 | 19344 | 10007 | 16126 | 6242 | 19344 | 4675 | 16126 | NA | NA | NA | NA |
| Szerlip M. et al 2018 | 62 | 12 | 55 | 14 | 609 | 657 | 935 | 1004 | 205 | 657 | 353 | 1004 | 108 | 657 | 185 | 1004 |
| Yousif N. et al 2018 | 58.4 | 11.7 | 51.5 | 13.9 | 216 | 281 | 187 | 265 | 53 | 281 | 79 | 265 | 47 | 281 | 56 | 265 |
| Zadok O. et al 2018 2008-2012 | 60 | 0 | 53.33 | 11.16 | 465 | 523 | 316 | 372 | 162 | 523 | 134 | 372 | 63 | 523 | 60 | 372 |
| Zadok O. et al 2018 2013-2016 | 58.33 | 3.71 | 55 | 11.14 | 720 | 847 | 685 | 787 | 330 | 847 | 331 | 787 | 110 | 847 | 142 | 787 |
| Bagienski M. et al 2017 | 61.67 | 7.59 | 50 | 15.38 | 56 | 61 | 38 | 40 | 22 | 61 | 13 | 40 | 6 | 61 | 4 | 40 |
| Bauer T. et al 2017 | NA | NA | NA | NA | NA | NA | NA | NA | 1150 | 8456 | 1059 | 7160 | NA | NA | NA | NA |
| Czarnecki A. et al 2017 | NA | NA | NA | NA | 436 | 453 | 520 | 546 | 208 | 453 | 275 | 546 | 40 | 453 | 42 | 546 |
| Katz M. et al 2017 | 61.9 | 13.4 | 55.4 | 15.6 | 323 | 418 | 294 | 401 | 114 | 418 | 144 | 401 | 26 | 418 | 39 | 401 |
| Chandrasekhar J. et al 2016 | NA | NA | NA | NA | NA | NA | NA | NA | 4133 | 11808 | 4679 | 11844 | 1372 | 11808 | 1524 | 11844 |
| Forrest J. et al 2016 | 57.7 | 12.3 | 51 | 14.3 | 1582 | 1708 | 1836 | 1979 | 601 | 1708 | 784 | 1979 | 191 | 1708 | 295 | 1979 |
| Kodali S. et al 2016 | 55.5 | 11.9 | 49.5 | 13.3 | 1137 | 1220 | 1212 | 1339 | 400 | 1220 | 537 | 1339 | 300 | 1220 | 371 | 1339 |
| Gaglia M. et al 2016 | NA | NA | NA | NA | 342 | 383 | 335 | 372 | 114 | 383 | 124 | 372 | NA | NA | NA | NA |
| Gilard M. et al 2016 | NA | NA | NA | NA | NA | NA | NA | NA | NA | NA | NA | NA | NA | NA | NA | NA |
| Codner P. et al 2015 | NA | NA | NA | NA | NA | NA | NA | NA | 59 | 203 | 63 | 157 | NA | NA | NA | NA |
| Al-Lamee R. et al 2014 | NA | NA | NA | NA | NA | NA | NA | NA | 144 | 756 | 207 | 1627 | 63 | 756 | 79 | 871 |
| Conrotto F. et al 2014 | 54 | 12.4 | 49.9 | 12.7 | 384 | 464 | 304 | 372 | 142 | 464 | 110 | 372 | 50 | 464 | 63 | 372 |
| Madershahian N. et al 2014 | 59.5 | 15.4 | 52.6 | 12 | 25 | 29 | 23 | 26 | 10 | 29 | 10 | 26 | NA | NA | NA | NA |
| Onorati F. et al 2014 | 53.1 | 11.4 | 48.2 | 12.6 | NA | NA | NA | NA | 103 | 428 | 76 | 297 | NA | NA | NA | NA |
| Sherif M. et al 2014 | 56 | 14 | 49 | 15 | NA | NA | NA | NA | 292 | 827 | 201 | 605 | 67 | 827 | 47 | 605 |
| Erez A. et al 2014 | 58 | 10 | 49 | 14 | NA | NA | NA | NA | 32 | 127 | 36 | 97 | NA | NA | NA | NA |
| Buja P. et al 2013 | 53 | 13 | 49 | 13 | 302 | 368 | 192 | 291 | 96 | 368 | 77 | 291 | 25 | 368 | 23 | 291 |
| D’Ascenzo F. et al 2013 | 54.7 | 11.3 | 49.22 | 13.5 | NA | NA | NA | NA | 18 | 216 | 3 | 161 | 17 | 216 | 10 | 161 |
| Ferrante G. et al 2013 | 53 | 11.91 | 51.33 | 10.43 | 271 | 348 | 245 | 308 | 86 | 348 | 90 | 308 | 30 | 348 | 23 | 308 |
| Hayashida K. et al 2012 | 53.5 | 12.9 | 47.2 | 14.6 | 93 | 131 | 91 | 129 | 24 | 131 | 37 | 129 | 13 | 131 | 20 | 129 |
| Humphries K. et al 2012 | 60 | 7.45 | 51.67 | 14.9 | 264 | 329 | 242 | 312 | 99 | 329 | 98 | 312 | 52 | 329 | 66 | 312 |
| Stangl V. et al 2012 | 54.3 | 8.4 | 46.7 | 14.8 | 52 | 58 | 39 | 42 | 21 | 58 | 24 | 42 | 7 | 58 | 6 | 42 |
| Buchanan G. et al 2011 | 52.2 | 12.6 | 50.8 | 12.9 | NA | NA | NA | NA | 32 | 146 | 56 | 159 | 23 | 146 | 25 | 159 |

# Supplementary Table S6: Baseline clinical characteristics & comorbidities in patients across included studies (Following):

| **Study ID** | **Prior MI*** | | | | **Prior PCI** | | | | **Prior Pacemaker implantation** | | | | **Atrial Fibrillation** | | | | **CKD** | | | | **COPD** | | | |
| --- | --- | --- | --- | --- | --- | --- | --- | --- | --- | --- | --- | --- | --- | --- | --- | --- | --- | --- | --- | --- | --- | --- | --- | --- |
|  | **Female** | | **Male** | | **Female** | | **Male** | | **Female** | | **Male** | | **Female** | | **Male** | | **Female** | | **Male** | | **Female** | | **Male** | |
|  | **Event n** | **Total** | **Event n** | **Total** | **Event n** | **Total** | **Event n** | **Total** | **Event n** | **Total** | **Event n** | **Total** | **Event n** | **Total** | **Event n** | **Total** | **Event n** | **Total** | **Event n** | **Total** | **Event n** | **Total** | **Event n** | **Total** |
| Sticchi A. et al 2025 | 162 | 844 | 163 | 844 | 249 | 844 | 258 | 844 | 94 | 844 | 112 | 844 | 293 | 844 | 275 | 844 | 593 | 844 | 479 | 844 | 168 | 844 | 169 | 844 |
| Al-Azizi K. et al 2025 | 174 | 1338 | 296 | 1678 | 455 | 1338 | 787 | 1678 | 181 | 1338 | 258 | 1678 | 458 | 1338 | 624 | 1678 | - | - | - | - | - | - | - | - |
| Matetic A. et al 2025 | 10 | 140 | 35 | 180 | 14 | 140 | 37 | 180 | - | - | - | - | 41 | 140 | 65 | 180 | - | - | - | - | 14 | 140 | 13 | 180 |
| Adamo M. et al 2024 / EVOLUT | 24 | 338 | 37 | 249 | - | - | - | - | 19 | 338 | 28 | 249 | 81 | 338 | 66 | 249 | 37 | 338 | 25 | 249 | 34 | 338 | 33 | 249 |
| Adamo M. et al 2024 / SAPIEN | 27 | 324 | 34 | 263 | - | - | - | - | 22 | 324 | 23 | 263 | 81 | 324 | 66 | 263 | 35 | 324 | 17 | 263 | 38 | 324 | 29 | 263 |
| Ahmad D. et al 2024 | 49 | 152 | 67 | 152 | 73 | 152 | 110 | 152 | 5 | 152 | 9 | 152 | 5 | 152 | 3 | 152 | - | - | - | - | 55 | 152 | 54 | 152 |
| Chiu C. et al 2024 | 11 | 54 | 11 | 46 | 17 | 54 | 16 | 46 | 4 | 54 | 1 | 46 | 12 | 54 | 11 | 46 | 19 | 54 | 22 | 46 | - | - | - | - |
| Dismorr M. et al 2024 | 817 | 4886 | 1561 | 5589 | 1095 | 4886 | 2161 | 5589 | 426 | 4886 | 718 | 5589 | 1865 | 4886 | 2546 | 5589 | - | - | - | - | 1045 | 4886 | 1145 | 5589 |
| Gabani R. et al 2024 | 512 | 6600 | 933 | 5653 | 919 | 6600 | 1380 | 5653 | - | - | - | - | 1633 | 6600 | 1423 | 5653 | - | - | - | - | - | - | - | - |
| Iwata J. et al 2024 | 11 | 343 | 9 | 168 | 64 | 343 | 52 | 168 | 16 | 343 | 9 | 168 | 70 | 343 | 34 | 168 | 251 | 343 | 118 | 168 | 39 | 343 | 44 | 168 |
| Kim M. et al 2024 | 52 | 657 | 108 | 755 | 141 | 457 | 264 | 755 | - | - | - | - | 161 | 657 | 242 | 755 | - | - | - | - | 73 | 657 | 104 | 755 |
| Lodo V. et al 2024 | - | - | - | - | - | - | - | - | - | - | - | - | - | - | - | - | - | - | - | - | 42 | 204 | 34 | 204 |
| Tateishi K. et al 2024 | - | - | - | - | 91 | 488 | 178 | 483 | - | - | - | - | - | - | - | - | - | - | - | - | - | - | - | - |
| Hoydahl M. et al 2024 | -- | -- | -- | -- | -- | -- | -- | -- | -- | -- | -- | -- | - | - | - | - | -- | -- | -- | -- | -- | -- | -- | -- |
| Zhou C. et al 2023 | 7091 | 54802 | 7220 | 54802 | 10716 | 54802 | 10852 | 54802 | 4971 | 54802 | 5006 | 54802 | 15649 | 54802 | 15478 | 54802 | 18870 | 54802 | 18943 | 54802 | 17553 | 54802 | 17538 | 54802 |
| Van Bergeijk K. et al 2023 | 30 | 466 | 63 | 512 | - | - | - | - | 42 | 466 | 60 | 512 | 140 | 466 | 173 | 512 | - | - | - | - | 76 | 466 | 113 | 512 |
| Leone P. et al 2023 | 18 | 99 | 15 | 99 | 26 | 99 | 25 | 99 | 11 | 99 | 12 | 99 | 17 | 99 | 17 | 99 | - | - | - | - | 18 | 99 | 18 | 99 |
| Malvindi P. et al 2023 | 12 | 276 | 30 | 204 | 45 | 276 | 68 | 204 | 11 | 276 | 22 | 204 | 65 | 276 | 64 | 204 | 161 | 276 | 115 | 204 | 59 | 276 | 50 | 204 |
| Elbaz-Geener G. et al 2022 | - | - | - | - | 5684 | 70170 | 8530 | 80477 | 7368 | 70170 | 12232 | 80477 | - | - | - | - | 21683 | 70170 | 32432 | 80477 | 21542 | 70170 | 24948 | 80477 |
| Alasnag M. et al 2022 | - | - | - | - | 88 | 347 | 158 | 448 | - | - | - | - | 76 | 347 | 68 | 448 | - | - | - | - | - | - | - | - |
| Medranda G. et al 2022 | - | - | - | - | - | - | - | - | - | - | - | - | - | - | - | - | - | - | - | - | - | - | - | - |
| Patel P. et al 2022 | 53 | 1041 | 135 | 1502 | - | - | - | - | 126 | 1041 | 214 | 1502 | 194 | 1041 | 362 | 1502 | 217 | 1041 | 365 | 1502 | 189 | 1041 | 288 | 1502 |
| Wohrle J. et al 2022 | - | - | - | - | - | - | - | - | 57 | 813 | 103 | 831 | - | - | - | - | 96 | 813 | 63 | 831 | 87 | 813 | 111 | 831 |
| He J. et al 2022 | 2 | 225 | 7 | 285 | 13 | 225 | 43 | 285 | - | - | - | - | 28 | 225 | 32 | 285 | 11 | 225 | 26 | 285 | 55 | 225 | 120 | 285 |
| Kuneman J. et al 2022 | 29 | 219 | 58 | 240 | 63 | 219 | 80 | 240 | - | - | - | - | 46 | 219 | 68 | 240 | - | - | - | - | 38 | 219 | 50 | 240 |
| Chiam P. et al 2021 | - | - | - | - | 144 | 475 | 157 | 398 | - | - | - | - | - | - | - | - | 27 | 475 | 26 | 398 | 12 | 475 | 25 | 398 |
| Deharo P. et al 2021 | 1625 | 12336 | 1634 | 12336 | 3594 | 12336 | 3539 | 12336 | 2524 | 12336 | 2506 | 12336 | 5604 | 12336 | 5565 | 12336 | 2110 | 12336 | 2145 | 12336 | 1668 | 12336 | 1707 | 12336 |
| Denegri A. et al 2021 | 222 | 2058 | 370 | 1615 | 444 | 2094 | 543 | 1634 | 215 | 2043 | 259 | 1594 | - | - | - | - | - | - | - | - | 301 | 2149 | 395 | 1672 |
| Gonçalves M. et al 2021 | 40 | 316 | 59 | 276 | 99 | 316 | 98 | 276 | NA | NA | NA | NA | 102 | 316 | 98 | 276 | 73 | 316 | 89 | 276 | NA | NA | NA | NA |
| Rodriguez-Gabella T. et al 2021 | 223 | 1565 | 245 | 1565 | 394 | 1565 | 420 | 1565 | 167 | 1565 | 165 | 1565 | 486 | 1565 | 505 | 1565 | 1089 | 1565 | 1063 | 1565 | 350 | 1565 | 385 | 1565 |
| Shishido K. et al 2021 | NA | NA | NA | NA | NA | NA | NA | NA | 111 | 1,793 | 55 | 795 | 363 | 1,793 | 186 | 795 | 1,095 | 1,793 | 457 | 795 | 192 | 1,793 | 193 | 795 |
| Simard T. et al 2021 | NA | NA | NA | NA | NA | NA | NA | NA | 1521 | 16900 | 1534 | 16900 | 5867 | 16900 | 5841 | 16900 | 5835 | 16900 | 5888 | 16900 | NA | NA | NA | NA |
| Useini D. et al 2021 | 8 | 58 | 9 | 45 | 19 | 58 | 21 | 45 | 8 | 58 | 6 | 45 | 26 | 58 | 20 | 45 | 18 | 58 | 25 | 45 | 3 | 58 | 8 | 45 |
| Chang H. et al 2020 | 8 | 125 | 6 | 96 | 28 | 125 | 47 | 96 | 2 | 125 | 3 | 96 | 29 | 125 | 18 | 96 | 105 | 125 | 77 | 96 | NA | NA | NA | NA |
| Chen S. et al 2020 | 3 | 50 | 2 | 50 | 11 | 50 | 19 | 50 | NA | NA | NA | NA | 13 | 50 | 7 | 50 | NA | NA | NA | NA | NA | NA | NA | NA |
| Stehli J. et al 2020 | NA | NA | NA | 355 | 56 | 328 | 85 | 355 | 28 | 328 | 58 | 355 | 96 | 328 | 112 | 355 | NA | NA | NA | NA | 41 | 328 | 64 | 355 |
| Tarantini G. et al 2020 | 61 | 834 | 119 | 860 | NA | NA | NA | NA | 75 | 834 | 120 | 860 | 161 | 808 | 204 | 824 | NA | NA | NA | NA | 103 | 834 | 153 | 860 |
| Wilczek K. et al 2020 | 42 | 131 | 54 | 141 | 27 | 125 | 29 | 134 | 17 | 126 | 31 | 139 | 35 | 120 | 49 | 133 | NA | NA | NA | NA | NA | NA | NA | NA |
| Van Mieghem N. et al 2020 | NA | NA | NA | NA | 51 | 366 | 133 | 498 | 27 | 366 | 60 | 498 | - | - | - | - | NA | NA | NA | NA | 99 | 366 | 206 | 498 |
| Chikata Y. et al 2020 | 24 | 748 | 41 | 342 | NA | NA | NA | NA | NA | NA | NA | NA | 187 | 746 | 76 | 342 | 497 | 748 | 216 | 342 | 63 | 748 | 49 | 342 |
| Linke A. et al 2019 | 72 | 1015 | 146 | 786 | 177 | 1015 | 234 | 787 | NA | NA | NA | NA | 435 | 1026 | 366 | 792 | 355 | 1026 | 206 | 791 | NA | NA | NA | NA |
| Pighi M. et al 2019 | NA | NA | NA | NA | NA | NA | NA | NA | 31 | 340 | 62 | 419 | 126 | 340 | 157 | 419 | 5 | 340 | 6 | 419 | 57 | 340 | 93 | 419 |
| Wang T. et al 2019 | 24 | 144 | 47 | 154 | NA | NA | NA | NA | 18 | 144 | 20 | 154 | 39 | 144 | 69 | 154 | NA | NA | NA | NA | 22 | 144 | 40 | 154 |
| Singh A. et al 2019 |  |  |  |  |  |  |  |  |  |  |  |  | - | - | - | - |  |  |  |  |  |  |  |  |
| Kilic A. et al 2019 | 171 | 518 | 232 | 518 | 155 | 518 | 206 | 518 | NA | NA | NA | NA | - | - | - | - | 17 | 518 | 33 | 518 | 67 | 518 | 63 | 518 |
| Kaier K. et al 2018 | 117 | 19344 | 148 | 16126 | NA | NA | NA | NA | NA | NA | NA | NA | 8923 | 19344 | 7457 | 16126 | NA | NA | NA | NA | 2454 | 19344 | 2821 | 16126 |
| Szerlip M. et al 2018 | 69 | 657 | 220 | 1004 | 162 | 657 | 383 | 1004 | 73 | 657 | 165 | 1004 | - | - | - | - | NA | NA | NA | NA | 57 | 657 | 57 | 1004 |
| Yousif N. et al 2018 | NA | NA | NA | NA | NA | NA | NA | NA | NA | NA | NA | NA | - | - | - | - | NA | NA | NA | NA | 36 | 281 | 67 | 265 |
| Zadok O. et al 2018 2008-2012 | NA | NA | NA | NA | NA | NA | NA | NA | NA | NA | NA | NA | - | - | - | - | NA | NA | NA | NA | NA | NA | NA | NA |
| Zadok O. et al 2018 2013-2016 | NA | NA | NA | NA | NA | NA | NA | NA | NA | NA | NA | NA | - | - | - | - | NA | NA | NA | NA | NA | NA | NA | NA |
| Bagienski M. et al 2017 | 10 | 61 | 21 | 40 | 16 | 61 | 13 | 40 | 6 | 61 | 5 | 40 | 26 | 61 | 9 | 40 | NA | NA | NA | NA | 3 | 61 | 6 | 40 |
| Bauer T. et al 2017 | 972 | 8456 | 1553 | 7160 | 1953 | 8456 | 2620 | 7160 | 795 | 8456 | 981 | 7160 | 2410 | 8456 | 2212 | 7160 | 702 | 8456 | 1031 | 7160 | 1725 | 8456 | 1926 | 7160 |
| Czarnecki A. et al 2017 | 83 | 453 | 140 | 546 | 137 | 453 | 182 | 546 | 20 | 453 | 49 | 546 | 134 | 453 | 184 | 546 | NA | NA | NA | NA | 74 | 453 | 102 | 546 |
| Katz M. et al 2017 | 41 | 418 | 78 | 401 | NA | NA | NA | NA | NA | NA | NA | NA | - | - | - | - | 335 | 418 | 278 | 401 | 65 | 418 | 89 | 401 |
| Chandrasekhar J. et al 2016 | NA | NA | NA | NA | 3485 | 11808 | 4965 | 11844 | 1724 | 11808 | 2225 | 11844 | 4598 | 11808 | 5059 | 11844 | NA | NA | NA | NA | NA | NA | NA | NA |
| Forrest J. et al 2016 | 336 | 1708 | 680 | 1979 | 532 | 1708 | 874 | 1979 | 308 | 1708 | 525 | 1979 | 699 | 1708 | 927 | 1979 | 283 | 1708 | 161 | 1979 | NA | NA | NA | NA |
| Kodali S. et al 2016 | 218 | 1220 | 449 | 1339 | 426 | 1220 | 593 | 1339 | 193 | 1220 | 356 | 1339 | - | - | - | - | 149 | 1220 | 272 | 1339 | 525 | 1220 | 617 | 1339 |
| Gaglia M. et al 2016 | 50 | 383 | 81 | 372 | 91 | 383 | 127 | 372 | NA | NA | NA | NA | 135 | 383 | 161 | 372 | 151 | 383 | 185 | 372 | 128 | 383 | 120 | 372 |
| Gilard M. et al 2016 | NA | NA | NA | NA | NA | NA | NA | NA | NA | NA | NA | NA | - | - | - | - | NA | NA | NA | NA | NA | NA | NA | NA |
| Codner P. et al 2015 | NA | NA | NA | NA | NA | NA | NA | NA | NA | NA | NA | NA | - | - | - | - | NA | NA | NA | NA | NA | NA | NA | NA |
| Al-Lamee R. et al 2014 | 98 | 756 | 256 | 871 | 81 | 756 | 157 | 871 | NA | NA | NA | NA | - | - | - | - | 28 | 756 | 59 | 871 | 152 | 756 | 214 | 871 |
| Conrotto F. et al 2014 | 75 | 464 | 99 | 372 | 146 | 464 | 162 | 372 | NA | NA | NA | NA | - | - | - | - | 84 | 464 | 65 | 372 | 98 | 464 | 145 | 372 |
| Madershahian N. et al 2014 | NA | NA | NA | NA | NA | NA | NA | NA | NA | NA | NA | NA | - | - | - | - | NA | NA | NA | NA | NA | NA | NA | NA |
| Onorati F. et al 2014 | 13 | 428 | 7 | 297 | 87 | 428 | 95 | 297 | NA | NA | NA | NA | - | - | - | - | NA | NA | NA | NA | 102 | 428 | 107 | 297 |
| Sherif M. et al 2014 | 95 | 827 | 132 | 605 | 250 | 827 | 245 | 605 | 94 | 827 | 87 | 605 | 25.8 | 827 | 23.8 | 605 | 556 | 827 | 313 | 605 | 172 | 827 | 171 | 605 |
| Erez A. et al 2014 | NA | NA | NA | NA | NA | NA | NA | NA | NA | NA | NA | NA | - | - | - | - | 81 | 127 | 76 | 97 | 35 | 127 | 39 | 97 |
| Buja P. et al 2013 | 49 | 368 | 94 | 291 | 82 | 368 | 106 | 291 | NA | NA | NA | NA | 63 | 368 | 45 | 291 | 71 | 368 | 83 | 291 | 61 | 368 | 78 | 291 |
| D’Ascenzo F. et al 2013 | 29 | 216 | 46 | 161 | 63 | 216 | 71 | 161 | NA | NA | NA | NA | - | - | - | - | 155 | 216 | 128 | 161 | 43 | 216 | 62 | 161 |
| Ferrante G. et al 2013 | 47 | 348 | 57 | 308 | 99 | 348 | 115 | 308 | NA | NA | NA | NA | - | - | - | - | NA | NA | NA | NA | NA | NA | NA | NA |
| Hayashida K. et al 2012 | 10 | 131 | 28 | 129 | 33 | 131 | 46 | 129 | NA | NA | NA | NA | - | - | - | - | 90 | 131 | 84 | 129 | 50 | 131 | 47 | 129 |
| Humphries K. et al 2012 | 117 | 329 | 142 | 312 | 130 | 329 | 198 | 312 | NA | NA | NA | NA | 107 | 329 | 121 | 312 | 226 | 329 | 187 | 312 | 70 | 329 | 100 | 312 |
| Stangl V. et al 2012 | 4 | 58 | 11 | 42 | 15 | 58 | 26 | 42 | 10 | 58 | 7 | 42 | 13 | 58 | 6 | 42 | NA | NA | NA | NA | NA | NA | NA | NA |
| Buchanan G. et al 2011 | 21 | 146 | 45 | 159 | 22 | 146 | 35 | 159 | NA | NA | NA | NA | - | - | - | - | 34 | 146 | 66 | 159 | 44 | 146 | 72 | 159 |

# Supplementary Table S7: Frailty across included studies

# Supplementary Table S8: Valve generation across included studies

| **Study** | **Country** | **Region** | **Valve generation** | **Valve platform** |
| --- | --- | --- | --- | --- |
| Adamo M. et al 2024 | Multinational | Multinational | 4th generation | Mixed platforms |
| Ahmad D. et al 2024 | USA | North America | Not specified | Mixed platforms |
| Al-Azizi K. et al 2025 | USA | North America | Mixed/Multiple generations | Mixed platforms |
| Al-Lamee R. et al 2014 | UK | Europe/Other | 1st generation | Mixed platforms |
| Malvindi P. et al 2023 | Italy | Europe/Other | Mixed/Multiple generations | Mixed platforms |
| Matetic A. et al 2025 | Croatia | Europe/Other | Mixed/Multiple generations | Balloon-expandable |
| Medranda G. et al 2022 | USA | North America | Mixed/Multiple generations | Balloon-expandable |
| Onorati F. et al 2014 | Multinational | Multinational | Mixed/Multiple generations | Balloon-expandable |
| Pighi M. et al 2019 | Multinational | Multinational | Not specified | Not specified |
| Rodriguez-Gabella T. et al 2021 | Spain | Europe/Other | Mixed/Multiple generations | Mixed platforms |
| Sherif M. et al 2014 | Germany | Europe/Other | 1st generation | Balloon-expandable |
| Shishido K. et al 2021 | Japan | East Asia | Mixed/Multiple generations | Balloon-expandable |
| Singh A. et al 2019 | United Kingdom | Europe/Other | Not specified | Not specified |
| Stangl V. et al 2012 | Germany | Europe/Other | 1st generation | Balloon-expandable |
| Stehli J. et al 2020 | Australia | Europe/Other | Mixed/Multiple generations | Balloon-expandable |
| Sticchi A. et al 2025 | Multinational | Multinational | Mixed/Multiple generations | Self-expanding |
| Tarantini G. et al 2020 | Multinational | Multinational | 3rd generation | Balloon-expandable |
| Tateishi K. et al 2024 | USA | North America | Mixed/Multiple generations | Mixed platforms |
| Useini D. et al 2021 | Germany | Europe/Other | 3rd generation | Balloon-expandable |
| van Bergeijk K. et al 2023 | Netherlands | Europe/Other | Not specified | Not specified |
| Van Mieghem N. et al 2020 | Netherlands | Europe/Other | Mixed/Multiple generations | Self-expanding |
| Wang T. et al 2019 | USA | North America | 3rd generation | Balloon-expandable |
| Wilczek K. et al 2020 | Poland | Europe/Other | Mixed/Multiple generations | Balloon-expandable |
| Wöhrle J. et al 2022 |  | Unknown | Not specified | Balloon-expandable |
| Yousif N. et al 2018 | United Kingdom | Europe/Other | Mixed/Multiple generations | Self-expanding |
| Zadok O. et al 2018 | Israel | Europe/Other | Mixed/Multiple generations | Balloon-expandable |
| Zhou C. et al 2023 | USA | North America | Not specified | Not specified |
| Alasnag M. et al 2022 | Gulf Countries | Europe/Other | Not specified | Mixed platforms |
| Chiu C. et al 2024 | Taiwan | Europe/Other | 1st generation | Mixed platforms |
| Ferrante G. et al 2014 |  | Unknown | 1st generation | Balloon-expandable |
| Gonçalves M. et al 2021 | USA | North America | Mixed/Multiple generations | Balloon-expandable |
| Iwata J. et al 2024 | Japan | East Asia | Mixed/Multiple generations | Mixed platforms |
| Kodali S. et al 2016 | Multinational | Multinational | 1st generation | Balloon-expandable |
| Madershahian N. et al 2014 | Germany | Europe/Other | Mixed/Multiple generations | Balloon-expandable |
| Patel P. et al 2022 | USA | North America | Mixed/Multiple generations | Balloon-expandable |
| Simard T. et al 2021 | USA | North America | 3rd generation | Balloon-expandable |
| Szerlip M. et al 2018 | USA | North America | 3rd generation | Balloon-expandable |
| Bagienski M. et al 2017 | Poland | Europe/Other | Mixed/Multiple generations | Balloon-expandable |
| Bauer T. et al 2017 | Germany | Europe/Other | Not specified | Self-expanding |
| Buchanan G. et al 2011 | Italy | Europe/Other | Mixed/Multiple generations | Balloon-expandable |
| Buja P. et al 2013 | Italy | Europe/Other | 1st generation | Self-expanding |
| Chandrasekhar J. et al 2016 | USA | North America | Not specified | Not specified |
| Chang H. et al 2020 | Taiwan | Europe/Other | Mixed/Multiple generations | Balloon-expandable |
| Chen S. et al 2020 | Taiwan | Europe/Other | Mixed/Multiple generations | Balloon-expandable |
| Chiam P. et al 2021 | East Asia | Europe/Other | Mixed/Multiple generations | Balloon-expandable |
| Chikata Y. et al 2020 | Japan | East Asia | Not specified | Not specified |
| Codner P. et al 2015 | Israel | Europe/Other | Mixed/Multiple generations | Balloon-expandable |
| Conrotto F. et al 2014 | Multinational | Multinational | Mixed/Multiple generations | Balloon-expandable |
| Czarnecki A. et al 2017 | Canada | North America | Mixed/Multiple generations | Balloon-expandable |
| D'Ascenzo F. et al 2013 |  | Unknown | 1st generation | Balloon-expandable |
| Deharo P. et al 2021 | France | Europe/Other | Mixed/Multiple generations | Balloon-expandable |
| Denegri A. et al 2021 | Italy | Europe/Other | Mixed/Multiple generations | Self-expanding |
| Dismorr M. et al 2024 | Sweden | Europe/Other | Mixed/Multiple generations | Balloon-expandable |
| Elbaz-Greener G. et al 2022 |  | Unknown | Not specified | Not specified |
| Erez A. et al 2014 | Israel | Europe/Other | Mixed/Multiple generations | Balloon-expandable |
| Forrest J. et al 2016 | USA | North America | 1st generation | Self-expanding |
| Gabani R. et al 2024 | Sapin | Europe/Other | Mixed/Multiple generations | Self-expanding |
| Gaglia M. et al 2016 | USA | North America | Mixed/Multiple generations | Mixed platforms |
| Gilard M. et al 2016 | Multinational | Multinational | Mixed/Multiple generations | Mixed platforms |
| Hayashida K. et al 2012 | France | Europe/Other | Mixed/Multiple generations | Balloon-expandable |
| He J. et al 2022 | China | East Asia | 1st generation | Self-expanding |
| Hoydahl M. et al 2024 | Norway | Europe/Other | Mixed/Multiple generations | Balloon-expandable |
| Humphries K. et al 2012 | Canada | North America | Not specified | Not specified |
| Kaier K. et al 2018 | Germany | Europe/Other | Not specified | Not specified |
| Katz M. et al 2017 | Brazil | Europe/Other | Mixed/Multiple generations | Balloon-expandable |
| Kilic A. et al 2019 | USA | North America | Mixed/Multiple generations | Mixed platforms |
| Kim M. et al 2024 | Multinational | Multinational | Not specified | Mixed platforms |
| Kuneman J.H. et al 2022 |  | Unknown | Mixed/Multiple generations | Balloon-expandable |
| Leone P.P. et al 2023 |  | Unknown | Mixed/Multiple generations | Mixed platforms |
| Linke A. et al 2019 | Germany | Europe/Other | Not specified | Mixed platforms |
| Lodo V. et al 2024 | Italy | Europe/Other | Not specified | Not specified |

# Supplementary Table S9. Comparisons of The Original Curves to The Reconstructed Curves for All-cause Mortality

| **Study name** | **Original** | **Reconstructed** |
| --- | --- | --- |
| **Adamo M. et al 2024** | Adamo M, Branca L, Pezzola E, et al. Sex-Related Outcomes of Transcatheter Aortic Valve Implantation With Self-Expanding or Balloon-Expandable Valves: Insights from the OPERA-TAVI Registry. Am J Cardiol. 2024;219:60-70. doi:10.1016/j.amjcard.2024.01.028  Supplementary Figure 1 |  |
| **Adamo M. et al 2024** | Adamo M, Branca L, Pezzola E, et al. Sex-Related Outcomes of Transcatheter Aortic Valve Implantation With Self-Expanding or Balloon-Expandable Valves: Insights from the OPERA-TAVI Registry. Am J Cardiol. 2024;219:60-70. doi:10.1016/j.amjcard.2024.01.028  Supplementary Figure 1 |  |

| **Ahmad D. et al 2024** | Ahmad D, Sá MP, Makani A, et al. Transcatheter Aortic Valve Replacement in Patients With Small Aortic Annuli: A Propensity-Matched Analysis of Gender-Based Outcomes. Am J Cardiol. 2024;227:75-82. doi:10.1016/j.amjcard.2024.07.029  Figure 1B |  |
| --- | --- | --- |
| **Al-Azizi K. et al 2025** | Al-Azizi K, Pickering T, AboHajar MP et al . Incidence and Sex-Based Comparison of Prosthesis-Patient Mismatch in Patients Undergoing Transcatheter Aortic Valve Replacement, Journal of the Society for Cardiovascular Angiography & Interventions,2025,103621,https://doi.org/10.1016/j.jscai.2025.103621.   Figure 1D |  |
| **Al-Lamee R. et al 2014** | Al-Lamee R, Broyd C, Parker J, et al. Influence of gender on clinical outcomes following transcatheter aortic valve implantation from the UK transcatheter aortic valve implantation registry and the National Institute for Cardiovascular Outcomes Research. Am J Cardiol. 2014;113(3):522-528. doi:10.1016/j.amjcard.2013.10.024  Figure 6 |  |
| **Bagienski M. et al 2017** | Bagienski M, Tokarek T, Wiktorowicz A, et al. Sex-related differences in clinical outcomes and quality of life after transcatheter aortic valve implantation for severe aortic stenosis. Postepy Kardiol Interwencyjnej. 2017;13(3):233-239. doi:10.5114/aic.2017.70195  Figure 2 |  |

| **Buchanan G. et al 2011** | Buchanan GL, Chieffo A, Montorfano M, et al. The role of sex on VARC outcomes following transcatheter aortic valve implantation with both Edwards SAPIEN™ and Medtronic CoreValve ReValving System® devices: the Milan registry. EuroIntervention. 2011;7(5):556-563. doi:10.4244/EIJV7I5A91  Figure 2 |  |
| --- | --- | --- |
| **Buja P. et al 2013** | Buja P, Napodano M, Tamburino C, et al. Comparison of variables in men versus women undergoing transcatheter aortic valve implantation for severe aortic stenosis (from Italian Multicenter CoreValve registry). Am J Cardiol. 2013;111(1):88-93. doi:10.1016/j.amjcard.2012.08.051  Figure 1A |  |

| **Chandrasekhar J. et al 2016** | Chandrasekhar J, Dangas G, Yu J, et al. Sex-Based Differences in Outcomes With Transcatheter Aortic Valve Therapy: TVT Registry From 2011 to 2014. J Am Coll Cardiol. 2016;68(25):2733-2744. doi:10.1016/j.jacc.2016.10.041  Central illustration - Death |  |
| --- | --- | --- |
| **Chiam P. et al 2021** | Chiam PTL, Hayashida K, Watanabe Y, et al. Sex differences in patients undergoing transcatheter aortic valve replacement in Asia. Open Heart. 2021;8(1):e001541. doi:10.1136/openhrt-2020-001541  Figure 1 |  |

| **Chikata Y. et al 2020** | Chikata Y, Iwata H, Doi S, Funamizu T, Okazaki S, Dohi S, Higuchi R, Saji M, Takamisawa I, Tamura H, et al. Simultaneous Estimation of Gender Male and Atrial Fibrillation as Risk Factors for Adverse Outcomes Following Transcatheter Aortic Valve Implantation. Journal of Clinical Medicine. 2020; 9(12):3963. https://doi.org/10.3390/jcm9123963  Figure 2B |  |
| --- | --- | --- |
| **Chikata Y. et al 2020** | Chikata Y, Iwata H, Doi S, Funamizu T, Okazaki S, Dohi S, Higuchi R, Saji M, Takamisawa I, Tamura H, et al. Simultaneous Estimation of Gender Male and Atrial Fibrillation as Risk Factors for Adverse Outcomes Following Transcatheter Aortic Valve Implantation. Journal of Clinical Medicine. 2020; 9(12):3963. https://doi.org/10.3390/jcm9123963  Figure 2B |  |

| **Chiu C. et al 2024** | Chiu CA, Chen PR, Li YJ, et al. Female showed favorable left ventricle hypertrophy regression during post-TAVR follow-up. Kaohsiung J Med Sci. 2024;40(4):384-394. doi:10.1002/kjm2.12808  Figure 5A |  |
| --- | --- | --- |
| **Codner P. et al 2015** | Codner P, Orvin K, Assali A, et al. Long-Term Outcomes for Patients With Severe Symptomatic Aortic Stenosis Treated With Transcatheter Aortic Valve Implantation. Am J Cardiol. 2015;116(9):1391-1398. doi:10.1016/j.amjcard.2015.08.004  Figure 5 |  |

| **Deharo P. et al 2021** | Deharo P, Cuisset T, Bisson A, et al. Outcomes Following Aortic Stenosis Treatment (Transcatheter vs Surgical Replacement) in Women vs Men (From a Nationwide Analysis). Am J Cardiol. 2021;154:67-77. doi:10.1016/j.amjcard.2021.05.046  Figure 1 (Top panel) |  |
| --- | --- | --- |
| **Denegri A. et al 2021** | Denegri A, Romano M, Petronio AS, et al. Gender Differences after Transcatheter Aortic Valve Replacement (TAVR): Insights from the Italian Clinical Service Project. J Cardiovasc Dev Dis. 2021;8(9):114. Published 2021 Sep 15. doi:10.3390/jcdd8090114  Supplement figure 2 |  |

| **Dismorr M. et al 2024** | Dismorr M, Granbom-Koski M, Ellfors E, et al. Sex differences and long-term clinical outcomes after transcatheter aortic valve replacement: A SWEDEHEART study. Am Heart J. 2024;277:27-38. doi:10.1016/j.ahj.2024.07.018  Figure 1A |  |
| --- | --- | --- |
| **Erez A. et al 2014** | Erez A, Segev A, Medvedofsky D, et al. Factors affecting survival in men versus women following transcatheter aortic valve implantation. Am J Cardiol. 2014;113(4):701-705. doi:10.1016/j.amjcard.2013.10.047  Figure 1 |  |

| **Ferrante G. et al 2014** | Ferrante G, Pagnotta P, Petronio AS, et al. Sex differences in postprocedural aortic regurgitation and mid-term mortality after transcatheter aortic valve implantation. Catheter Cardiovasc Interv. 2014;84(2):264-271. doi:10.1002/ccd.25377  Figure 1 |  |
| --- | --- | --- |
| **Forrest J. et al 2016** | Forrest JK, Adams DH, Popma JJ, et al. Transcatheter Aortic Valve Replacement in Women Versus Men (from the US CoreValve Trials). Am J Cardiol. 2016;118(3):396-402. doi:10.1016/j.amjcard.2016.05.013  Figure 1A |  |

| **Gabani R. et al 2024** | Gabani R, Brugaletta S, Bujak K, et al. Impact of gender on in-hospital and long-term outcomes after transcatheter aortic valve implantation: an analysis of the Spanish TAVI registry. Rev Esp Cardiol (Engl Ed). 2025;78(4):338-346. doi:10.1016/j.rec.2024.08.002  Figure 2 |  |
| --- | --- | --- |
| **Gaglia M. et al 2016** | Gaglia MA Jr, Lipinski MJ, Torguson R, et al. Comparison in Men Versus Women of Co-morbidities, Complications, and Outcomes After Transcatheter Aortic Valve Implantation for Severe Aortic Stenosis. Am J Cardiol. 2016;118(11):1692-1697. doi:10.1016/j.amjcard.2016.08.049  Figure 1 |  |

| **Gilard M. et al 2016** | Gilard M, Schlüter M, Snow TM, et al. The 2011-2012 pilot European Society of Cardiology Sentinel Registry of Transcatheter Aortic Valve Implantation: 12-month clinical outcomes. EuroIntervention. 2016;12(1):79-87. doi:10.4244/EIJV12I1A15  Figure 3B |  |
| --- | --- | --- |
| **Hayashida K. et al 2012** | Hayashida K, Morice MC, Chevalier B, et al. Sex-related differences in clinical presentation and outcome of transcatheter aortic valve implantation for severe aortic stenosis. J Am Coll Cardiol. 2012;59(6):566-571. doi:10.1016/j.jacc.2011.10.877  Figure 2 |  |

| **He J. et al 2022** | He JJ, Xiong TY, Yao YJ, et al. Sex Difference in Outcomes Following Transcatheter Aortic Valve Replacement in Bicuspid Aortic Stenosis. JACC Cardiovasc Interv. 2022;15(16):1652-1660. doi:10.1016/j.jcin.2022.06.036  Figure 1 |  |
| --- | --- | --- |
| **Hoydahl M. et al 2024** | Høydahl MP, Busund R, Rösner A, Kjønås D. Transcatheter aortic valve implantation (from inception to standard treatment): a single-center observational study. Front Cardiovasc Med. 2024;11:1298346. Published 2024 Jan 15. doi:10.3389/fcvm.2024.1298346  Figure 3 |  |

| **Humphries K. et al 2012** | Humphries KH, Toggweiler S, Rodés-Cabau J, et al. Sex differences in mortality after transcatheter aortic valve replacement for severe aortic stenosis. J Am Coll Cardiol. 2012;60(10):882-886. doi:10.1016/j.jacc.2012.05.009  Figure 1 |  |
| --- | --- | --- |
| **Iwata J. et al 2024** | Iwata J, Hayashida K, Arita R, et al. Long-Term Impact of Gender Differences After Transcatheter Aortic Valve Implantation. CJC Open. 2024;6(12):1453-1461. Published 2024 Aug 30. doi:10.1016/j.cjco.2024.08.012  Figure 2A |  |

| **Katz M. et al 2017** | Katz M, Carlos Bacelar Nunes Filho A, Caixeta A, et al. Gender-related differences on short- and long-term outcomes of patients undergoing transcatheter aortic valve implantation. Catheter Cardiovasc Interv. 2017;89(3):429-436. doi:10.1002/ccd.26658  Figure 1 |  |
| --- | --- | --- |
| **Kilic A. et al 2019** | Kilic A, Bianco V, Gleason TG, et al. Longitudinal Outcomes of Women Undergoing Transcatheter Aortic Valve Replacement. Innovations (Phila). 2019;14(4):311-320. doi:10.1177/1556984519842943  Figure 1 |  |

| **Kim M. et al 2024** | Kim M, Kang DY, Ahn JM, et al. Sex-Specific Disparities in Clinical Outcomes After Transcatheter Aortic Valve Replacement Among Different Racial Populations. JACC Asia. 2024;4(4):292-302. Published 2024 Feb 13. doi:10.1016/j.jacasi.2023.11.016  Figure 2A |  |
| --- | --- | --- |
| **Kodali S. et al 2016** | Kodali S, Williams MR, Doshi D, et al. Sex-Specific Differences at Presentation and Outcomes Among Patients Undergoing Transcatheter Aortic Valve Replacement: A Cohort Study. Ann Intern Med. 2016;164(6):377-384. doi:10.7326/M15-0121  Appendix Figure A |  |

| **Kuneman J. et al 2022** | Kuneman JH, Butcher SC, Stassen J, et al. Interaction between sex and left ventricular reverse remodeling and its association with outcomes after transcatheter aortic valve implantation. Int J Cardiovasc Imaging. 2022;38(9):1973-1985. doi:10.1007/s10554-022-02596-x  Figure 4 |  |
| --- | --- | --- |
| **Leone P. et al 2023** | Leone PP, Gohar A, Pagnesi M, et al. Clinical outcomes in women and men with small aortic annuli undergoing transcatheter aortic valve implantation: A multicenter, retrospective, propensity score-matched comparison. Int J Cardiol. 2023;379:16-23. doi:10.1016/j.ijcard.2023.02.044   Figure 2 |  |

| **Linke A. et al 2019** | Linke A, Schlotter F, Haussig S, et al. Gender-dependent association of diabetes mellitus with mortality in patients undergoing transcatheter aortic valve replacement. Clin Res Cardiol. 2019;108(1):39-47. doi:10.1007/s00392-018-1309-0  Figure 1C |  |
| --- | --- | --- |
| **Lodo V. et al 2024** | Lodo V, Italiano EG, Weltert L, et al. The influence of gender on outcomes following transcatheter aortic valve implantation. Front Cardiovasc Med. 2024;11:1417430. Published 2024 Jul 17. doi:10.3389/fcvm.2024.1417430  Figure 2 |  |

| **Pighi M. et al 2019** | Pighi M, Piazza N, Martucci G, et al. Sex-Specific Determinants of Outcomes After Transcatheter Aortic Valve Replacement. Circ Cardiovasc Qual Outcomes. 2019;12(3):e005363. doi:10.1161/CIRCOUTCOMES.118.005363  Figure 2 |  |
| --- | --- | --- |
| **Sherif M. et al 2014** | Sherif MA, Zahn R, Gerckens U, et al. Effect of gender differences on 1-year mortality after transcatheter aortic valve implantation for severe aortic stenosis: results from a multicenter real-world registry. Clin Res Cardiol. 2014;103(8):613-620. doi:10.1007/s00392-014-0690-6  Figure 1 |  |

| **Shishido K. et al 2021** | Shishido K, Yamanaka F, Ochiai T, et al. Effect of Sex on Mortality and Left Ventricular Remodeling After Transcatheter Aortic Valve Implantation. Circ J. 2021;85(7):979-988. doi:10.1253/circj.CJ-20-1095  Figure 1A |  |
| --- | --- | --- |
| **Singh A. et al 2019** | Singh A, Musa TA, Treibel TA, et al. Sex differences in left ventricular remodelling, myocardial fibrosis and mortality after aortic valve replacement. Heart. 2019;105(23):1818-1824. doi:10.1136/heartjnl-2019-314987  Figure 3 (middle panel) |  |

| **Szerlip M. et al 2018** | Szerlip M, Gualano S, Holper E, et al. Sex-Specific Outcomes of Transcatheter Aortic Valve Replacement With the SAPIEN 3 Valve: Insights From the PARTNER II S3 High-Risk and Intermediate-Risk Cohorts. JACC Cardiovasc Interv. 2018;11(1):13-20. doi:10.1016/j.jcin.2017.09.035  Figure 1B |  |
| --- | --- | --- |
| **Tarantini G. et al 2020** | Tarantini G, Baumgartner H, Frank D, et al. Four-year mortality in women and men after transfemoral transcatheter aortic valve implantation using the SAPIEN 3. Catheter Cardiovasc Interv. 2021;97(5):876-884. doi:10.1002/ccd.29257  Figure 2A |  |

| **Useini D. et al 2021** | Useini D, Schlömicher M, Aweimer A, Haldenwang P, Strauch J, Patsalis P. Long-term Follow up after Transfemoral Transcatheter Aortic Valve Implantation in Lower Risk Patients Using the Balloon-Expandable Bioprosthesis: Gender-Dependent Outcomes. Heart Surg Forum. 2022;25(2):E175-E180. Published 2022 Mar 8. doi:10.1532/hsf.4451  Figure 1A |  |
| --- | --- | --- |
| **Wilczek K. et al 2020** | Wilczek K, Chodór P, Dyrbuś M, et al. Female gender and the clinical and periprocedural profile and clinical outcomes of transcatheter aortic valve implantation: experiences of a tertiary Polish centre. Postepy Kardiol Interwencyjnej. 2020;16(4):436-443. doi:10.5114/aic.2020.101769  Figure 1 |  |

| **Wohrle J. et al 2022** | Wöhrle J, Gilard M, Didier R, et al. Outcomes After Transcatheter Aortic Valve Implantation in Men Versus Women. Am J Cardiol. 2022;180:108-115. doi:10.1016/j.amjcard.2022.06.035  Figure 2B |  |
| --- | --- | --- |
| **Yousif N. et al 2018** | Yousif N, Obeid S, Binder R, et al. Impact of gender on outcomes after transcatheter aortic valve implantation. J Geriatr Cardiol. 2018;15(6):394-400. doi:10.11909/j.issn.1671-5411.2018.06.013  Figure 2 |  |

| **Zadok O. et al 2018** | Itzhaki Ben Zadok O, Kornowski R, Finkelstein A, et al. Temporal Trends in Gender-Related Differences and Outcomes in Patients Who Underwent Transcatheter Aortic Valve Implantation (from the Israeli Transcatheter Aortic Valve Implantation Multicenter Registry). Am J Cardiol. 2019;123(1):108-115. doi:10.1016/j.amjcard.2018.09.018  Figure 3 |  |
| --- | --- | --- |
| **Zadok O. et al 2018** | Itzhaki Ben Zadok O, Kornowski R, Finkelstein A, et al. Temporal Trends in Gender-Related Differences and Outcomes in Patients Who Underwent Transcatheter Aortic Valve Implantation (from the Israeli Transcatheter Aortic Valve Implantation Multicenter Registry). Am J Cardiol. 2019;123(1):108-115. doi:10.1016/j.amjcard.2018.09.018  Figure 3 |  |

# Supplementary Table S10: Landmark analysis results for All-cause mortality

# Supplementary Table S11: Cox Model Results: All-cause Mortality

**Cox Model Results: 00-ACM truncate follow-up at 10y**

**A. Usual Cox Regression**

**Cox Model Coefficients & 95% Confidence Intervals**

| exp(coef) | exp(-coef) | lower .95 | upper .95 |
| --- | --- | --- | --- |
| 0.8124661 | 1.230821 | 0.7938816 | 0.8314857 |

**Schoenfeld Residuals Test**

| Variable | chisq | df | p |
| --- | --- | --- | --- |
| relevel(Arm, ref = placebb) | 1.608508 | 1 | 0.2047016 |
| GLOBAL | 1.608508 | 1 | 0.2047016 |

**Schoenfeld Residuals Plot**

**B. Stratified Cox Regression**

**Cox Model Coefficients & 95% Confidence Intervals**

| exp(coef) | exp(-coef) | lower .95 | upper .95 |
| --- | --- | --- | --- |
| 0.8202037 | 1.219209 | 0.8013297 | 0.8395222 |

**Schoenfeld Residuals Test**

| Variable | chisq | df | p |
| --- | --- | --- | --- |
| relevel(Arm, ref = placebb) | 5.880787 | 1 | 0.01530698 |
| GLOBAL | 5.880787 | 1 | 0.01530698 |

**Schoenfeld Residuals Plot**

# Supplementary Table S12. Comparisons of The Original Curves to The Reconstructed Curves for Cardiovascular Mortality

| **Study name** | **Original** | **Reconstructed** |
| --- | --- | --- |
| **Buja P. et al 2013** | Buja P, Napodano M, Tamburino C, et al. Comparison of variables in men versus women undergoing transcatheter aortic valve implantation for severe aortic stenosis (from Italian Multicenter CoreValve registry). Am J Cardiol. 2013;111(1):88-93. doi:10.1016/j.amjcard.2012.08.051  Figure 1B |  |
| **Chikata Y. et al 2020** | Chikata Y, Iwata H, Doi S, Funamizu T, Okazaki S, Dohi S, Higuchi R, Saji M, Takamisawa I, Tamura H, et al. Simultaneous Estimation of Gender Male and Atrial Fibrillation as Risk Factors for Adverse Outcomes Following Transcatheter Aortic Valve Implantation. Journal of Clinical Medicine. 2020; 9(12):3963. https://doi.org/10.3390/jcm9123963  Figure 2B |  |

| **Chikata Y. et al 2020** | Chikata Y, Iwata H, Doi S, Funamizu T, Okazaki S, Dohi S, Higuchi R, Saji M, Takamisawa I, Tamura H, et al. Simultaneous Estimation of Gender Male and Atrial Fibrillation as Risk Factors for Adverse Outcomes Following Transcatheter Aortic Valve Implantation. Journal of Clinical Medicine. 2020; 9(12):3963. https://doi.org/10.3390/jcm9123963  Figure 2B |  |
| --- | --- | --- |
| **Deharo P. et al 2021** | Deharo P, Cuisset T, Bisson A, et al. Outcomes Following Aortic Stenosis Treatment (Transcatheter vs Surgical Replacement) in Women vs Men (From a Nationwide Analysis). Am J Cardiol. 2021;154:67-77. doi:10.1016/j.amjcard.2021.05.046  Figure 1 (lower panel) |  |

| **Denegri A. et al 2021** | Denegri A, Romano M, Petronio AS, et al. Gender Differences after Transcatheter Aortic Valve Replacement (TAVR): Insights from the Italian Clinical Service Project. J Cardiovasc Dev Dis. 2021;8(9):114. Published 2021 Sep 15. doi:10.3390/jcdd8090114  Figure 3 |  |
| --- | --- | --- |
| **Forrest J. et al 2016** | Forrest JK, Adams DH, Popma JJ, et al. Transcatheter Aortic Valve Replacement in Women Versus Men (from the US CoreValve Trials). Am J Cardiol. 2016;118(3):396-402. doi:10.1016/j.amjcard.2016.05.013  Figure 1B |  |

| **Iwata J. et al 2024** | Iwata J, Hayashida K, Arita R, et al. Long-Term Impact of Gender Differences After Transcatheter Aortic Valve Implantation. CJC Open. 2024;6(12):1453-1461. Published 2024 Aug 30. doi:10.1016/j.cjco.2024.08.012  Figure 2B |  |
| --- | --- | --- |
| **Shishido K. et al 2021** | Shishido K, Yamanaka F, Ochiai T, et al. Effect of Sex on Mortality and Left Ventricular Remodeling After Transcatheter Aortic Valve Implantation. Circ J. 2021;85(7):979-988. doi:10.1253/circj.CJ-20-1095  Figure 1B |  |

| **Singh A. et al 2019** | Singh A, Musa TA, Treibel TA, et al. Sex differences in left ventricular remodelling, myocardial fibrosis and mortality after aortic valve replacement. Heart. 2019;105(23):1818-1824. doi:10.1136/heartjnl-2019-314987  Figure 3 (middle panel) |  |
| --- | --- | --- |
| **Tarantini G. et al 2020** | Tarantini G, Baumgartner H, Frank D, et al. Four-year mortality in women and men after transfemoral transcatheter aortic valve implantation using the SAPIEN 3. Catheter Cardiovasc Interv. 2021;97(5):876-884. doi:10.1002/ccd.29257  Figure 2B |  |

| **Wohrle J. et al 2022** | Wöhrle J, Gilard M, Didier R, et al. Outcomes After Transcatheter Aortic Valve Implantation in Men Versus Women. Am J Cardiol. 2022;180:108-115. doi:10.1016/j.amjcard.2022.06.035  Figure 2C |  |
| --- | --- | --- |
|  |  |  |

# Supplementary Table S13: Landmark analysis results for Cardiovascular mortality

# Supplementary Table S14: Cox Model Results: Cardiovascular Mortality

A. Usual Cox Regression

Cox Model Coefficients & 95% Confidence Intervals

| exp(coef) | exp(-coef) | lower .95 | upper .95 |
| --- | --- | --- | --- |
| 0.8367592 | 1.195087 | 0.7838581 | 0.8932305 |

Schoenfeld Residuals Test

| Variable | chisq | df | p |
| --- | --- | --- | --- |
| relevel(Arm, ref = placebb) | 0.336931 | 1 | 0.5616061 |
| GLOBAL | 0.336931 | 1 | 0.5616061 |

Schoenfeld Residuals Plot

B. Stratified Cox Regression

Cox Model Coefficients & 95% Confidence Intervals

| exp(coef) | exp(-coef) | lower .95 | upper .95 |
| --- | --- | --- | --- |
| 0.8806015 | 1.135587 | 0.8244893 | 0.9405326 |

Schoenfeld Residuals Test

| Variable | chisq | df | p |
| --- | --- | --- | --- |
| relevel(Arm, ref = placebb) | 1.131108 | 1 | 0.2875391 |
| GLOBAL | 1.131108 | 1 | 0.2875391 |

Schoenfeld Residuals Plot

# Supplementary Table S15. Comparisons of The Original Curves to The Reconstructed Curves for Stroke/TIA

| **Study name** | **Original** | **Reconstructed** |
| --- | --- | --- |
| **Adamo M. et al 2024** | Adamo M, Branca L, Pezzola E, et al. Sex-Related Outcomes of Transcatheter Aortic Valve Implantation With Self-Expanding or Balloon-Expandable Valves: Insights from the OPERA-TAVI Registry. Am J Cardiol. 2024;219:60-70. doi:10.1016/j.amjcard.2024.01.028  Supplement Figure 5 |  |
| **Adamo M. et al 2024** | Adamo M, Branca L, Pezzola E, et al. Sex-Related Outcomes of Transcatheter Aortic Valve Implantation With Self-Expanding or Balloon-Expandable Valves: Insights from the OPERA-TAVI Registry. Am J Cardiol. 2024;219:60-70. doi:10.1016/j.amjcard.2024.01.028  Supplement figure 5 |  |
| **Ahmad D. et al 2024** | Ahmad D, Sá MP, Makani A, et al. Transcatheter Aortic Valve Replacement in Patients With Small Aortic Annuli: A Propensity-Matched Analysis of Gender-Based Outcomes. Am J Cardiol. 2024;227:75-82. doi:10.1016/j.amjcard.2024.07.029  Figure 2B |  |
| **Chandrasekhar J. et al 2016** | Chandrasekhar J, Dangas G, Yu J, et al. Sex-Based Differences in Outcomes With Transcatheter Aortic Valve Therapy: TVT Registry From 2011 to 2014. J Am Coll Cardiol. 2016;68(25):2733-2744. doi:10.1016/j.jacc.2016.10.041  Central illustration - Stroke |  |

| **Denegri A. et al 2021** | Denegri A, Romano M, Petronio AS, et al. Gender Differences after Transcatheter Aortic Valve Replacement (TAVR): Insights from the Italian Clinical Service Project. J Cardiovasc Dev Dis. 2021;8(9):114. Published 2021 Sep 15. doi:10.3390/jcdd8090114  Supplement Figure 1 |  |
| --- | --- | --- |
| **Denegri A. et al 2021** | Denegri A, Romano M, Petronio AS, et al. Gender Differences after Transcatheter Aortic Valve Replacement (TAVR): Insights from the Italian Clinical Service Project. J Cardiovasc Dev Dis. 2021;8(9):114. Published 2021 Sep 15. doi:10.3390/jcdd8090114  Supplement Figure 1 |  |

| **Dismorr M. et al 2024** | Dismorr M, Granbom-Koski M, Ellfors E, et al. Sex differences and long-term clinical outcomes after transcatheter aortic valve replacement: A SWEDEHEART study. Am Heart J. 2024;277:27-38. doi:10.1016/j.ahj.2024.07.018  Figure 6A |  |
| --- | --- | --- |
| **Kim M. et al 2024** | Kim M, Kang DY, Ahn JM, et al. Sex-Specific Disparities in Clinical Outcomes After Transcatheter Aortic Valve Replacement Among Different Racial Populations. JACC Asia. 2024;4(4):292-302. Published 2024 Feb 13. doi:10.1016/j.jacasi.2023.11.016  Figure 2B |  |

| **Kodali S. et al 2016** | Kodali S, Williams MR, Doshi D, et al. Sex-Specific Differences at Presentation and Outcomes Among Patients Undergoing Transcatheter Aortic Valve Replacement: A Cohort Study. Ann Intern Med. 2016;164(6):377-384. doi:10.7326/M15-0121  Appendix figure B |  |
| --- | --- | --- |
| **Useini D. et al 2021** | Useini D, Schlömicher M, Aweimer A, Haldenwang P, Strauch J, Patsalis P. Long-term Follow up after Transfemoral Transcatheter Aortic Valve Implantation in Lower Risk Patients Using the Balloon-Expandable Bioprosthesis: Gender-Dependent Outcomes. Heart Surg Forum. 2022;25(2):E175-E180. Published 2022 Mar 8. doi:10.1532/hsf.4451  Figure 1B |  |

# Supplementary Table S16: Landmark analysis results for Stroke/TIA:

# Supplementary Table S17: Cox Model Results: 000-Stroke

**Cox Model Results: 000-Stroke truncate follow-up at 10**

**A. Usual Cox Regression**

**Cox Model Coefficients & 95% Confidence Intervals**

| **exp(coef)** | **exp(-coef)** | **lower .95** | **upper .95** |
| --- | --- | --- | --- |
| **1.036556** | **0.9647329** | **0.9674261** | **1.110627** |

**Schoenfeld Residuals Test**

| **Variable** | **chisq** | **df** | **p** |
| --- | --- | --- | --- |
| **relevel(Arm, ref = placebb)** | **8.529672** | **1** | **0.003494026** |
| **GLOBAL** | **8.529672** | **1** | **0.003494026** |

**Schoenfeld Residuals Plot**

**B. Stratified Cox Regression**

**Cox Model Coefficients & 95% Confidence Intervals**

| **exp(coef)** | **exp(-coef)** | **lower .95** | **upper .95** |
| --- | --- | --- | --- |
| **1.038715** | **0.9627278** | **0.9693923** | **1.112995** |

**Schoenfeld Residuals Test**

| **Variable** | **chisq** | **df** | **p** |
| --- | --- | --- | --- |
| **relevel(Arm, ref = placebb)** | **8.271114** | **1** | **0.004028079** |
| **GLOBAL** | **8.271114** | **1** | **0.004028079** |

**Schoenfeld Residuals Plot**

# Supplementary Table S18. Comparisons of The Original Curves to The Reconstructed Curves for Myocardial Infarction

| **Study name** | **Original** | **Reconstructed** |
| --- | --- | --- |
| **Chandrasekhar J. et al 2016** | Chandrasekhar J, Dangas G, Yu J, et al. Sex-Based Differences in Outcomes With Transcatheter Aortic Valve Therapy: TVT Registry From 2011 to 2014. J Am Coll Cardiol. 2016;68(25):2733-2744. doi:10.1016/j.jacc.2016.10.041  Central illustration - Myocardial Infarction |  |
| **Dismorr M. et al 2024** | Dismorr M, Granbom-Koski M, Ellfors E, et al. Sex differences and long-term clinical outcomes after transcatheter aortic valve replacement: A SWEDEHEART study. Am Heart J. 2024;277:27-38. doi:10.1016/j.ahj.2024.07.018  Figure 4A |  |

| **Wohrle J. et al 2022** | Wöhrle J, Gilard M, Didier R, et al. Outcomes After Transcatheter Aortic Valve Implantation in Men Versus Women. Am J Cardiol. 2022;180:108-115. doi:10.1016/j.amjcard.2022.06.035  Figure 2E |  |
| --- | --- | --- |

# Supplementary Table S19: Landmark analysis results for Myocardial Infarction:

# Supplementary Table S20: Cox Model Results: 000-MI

A. Usual Cox Regression

Cox Model Coefficients & 95% Confidence Intervals

| exp(coef) | exp(-coef) | lower .95 | upper .95 |
| --- | --- | --- | --- |
| 1.031583 | 0.9693842 | 0.9349788 | 1.138168 |

Schoenfeld Residuals Test

| Variable | chisq | df | p |
| --- | --- | --- | --- |
| relevel(Arm, ref = placebb) | 6.951198 | 1 | 0.008376313 |
| GLOBAL | 6.951198 | 1 | 0.008376313 |

Schoenfeld Residuals Plot

B. Stratified Cox Regression

Cox Model Coefficients & 95% Confidence Intervals

| exp(coef) | exp(-coef) | lower .95 | upper .95 |
| --- | --- | --- | --- |
| 1.028061 | 0.9727053 | 0.9317639 | 1.134309 |

Schoenfeld Residuals Test

| Variable | chisq | df | p |
| --- | --- | --- | --- |
| relevel(Arm, ref = placebb) | 7.47138 | 1 | 0.006268748 |
| GLOBAL | 7.47138 | 1 | 0.006268748 |

Schoenfeld Residuals Plot

# Supplementary Table S21. Comparisons of The Original Curves to The Reconstructed Curves for All-cause Rehospitalizations

| **Study name** | **Original** | **Reconstructed** |
| --- | --- | --- |
| **Kim M. et al 2024** | Kim M, Kang DY, Ahn JM, et al. Sex-Specific Disparities in Clinical Outcomes After Transcatheter Aortic Valve Replacement Among Different Racial Populations. JACC Asia. 2024;4(4):292-302. Published 2024 Feb 13. doi:10.1016/j.jacasi.2023.11.016  Figure 2C |  |
| **Kilic A. et al 2019** | Kilic A, Bianco V, Gleason TG, et al. Longitudinal Outcomes of Women Undergoing Transcatheter Aortic Valve Replacement. Innovations (Phila). 2019;14(4):311-320. doi:10.1177/1556984519842943 |  |
| **Kodali S. et al 2016** | Kodali S, Williams MR, Doshi D, et al. Sex-Specific Differences at Presentation and Outcomes Among Patients Undergoing Transcatheter Aortic Valve Replacement: A Cohort Study. Ann Intern Med. 2016;164(6):377-384. doi:10.7326/M15-0121  Appendix figure C |  |

| **Zhou C. et al 2023** | Zhou C, Xia Z, Chen B, Song Y, Lian Z. Gender Differences in Age-Stratified Early Outcomes in Patients With Transcatheter Aortic Valve Implantation. Am J Cardiol. 2023;187:100-109. doi:10.1016/j.amjcard.2022.10.038  Figure 2 |  |
| --- | --- | --- |
| **Zhou C. et al 2023** | Zhou C, Xia Z, Chen B, Song Y, Lian Z. Gender Differences in Age-Stratified Early Outcomes in Patients With Transcatheter Aortic Valve Implantation. Am J Cardiol. 2023;187:100-109. doi:10.1016/j.amjcard.2022.10.038  Figure 2 |  |

| **Zhou C. et al 2023** | Zhou C, Xia Z, Chen B, Song Y, Lian Z. Gender Differences in Age-Stratified Early Outcomes in Patients With Transcatheter Aortic Valve Implantation. Am J Cardiol. 2023;187:100-109. doi:10.1016/j.amjcard.2022.10.038  Figure 2 |  |
| --- | --- | --- |
| **Zhou C. et al 2023** | Zhou C, Xia Z, Chen B, Song Y, Lian Z. Gender Differences in Age-Stratified Early Outcomes in Patients With Transcatheter Aortic Valve Implantation. Am J Cardiol. 2023;187:100-109. doi:10.1016/j.amjcard.2022.10.038  Figure 2 |  |

# Supplementary Table S22: Landmark analysis results for All-cause Rehospitalizations:

# Supplementary Table S23: Cox Model Results: 000-Rehospitalization

A. Usual Cox Regression

Cox Model Coefficients & 95% Confidence Intervals

| exp(coef) | exp(-coef) | lower .95 | upper .95 |
| --- | --- | --- | --- |
| 1.039646 | 0.9618654 | 1.008435 | 1.071824 |

Schoenfeld Residuals Test

| Variable | chisq | df | p |
| --- | --- | --- | --- |
| relevel(Arm, ref = placebb) | 5.682513 | 1 | 0.01713481 |
| GLOBAL | 5.682513 | 1 | 0.01713481 |

Schoenfeld Residuals Plot

B. Stratified Cox Regression

Cox Model Coefficients & 95% Confidence Intervals

| exp(coef) | exp(-coef) | lower .95 | upper .95 |
| --- | --- | --- | --- |
| 1.037822 | 0.9635562 | 1.006664 | 1.069945 |

Schoenfeld Residuals Test

| Variable | chisq | df | p |
| --- | --- | --- | --- |
| relevel(Arm, ref = placebb) | 5.422972 | 1 | 0.0198735 |
| GLOBAL | 5.422972 | 1 | 0.0198735 |

Schoenfeld Residuals Plot

# Supplementary Table S24: Comparison of reconstructed curves and original curves for HF hospitalizations

| **Study name** | **Original** | **Reconstructed** |
| --- | --- | --- |
| **Adamo M.et al 2024** | Adamo M, Branca L, Pezzola E, et al. Sex-Related Outcomes of Transcatheter Aortic Valve Implantation With Self-Expanding or Balloon-Expandable Valves: Insights from the OPERA-TAVI Registry. Am J Cardiol. 2024;219:60-70. doi:10.1016/j.amjcard.2024.01.028  Supplementary Figure 2 |  |
| **Adamo M. et al 2024** | Adamo M, Branca L, Pezzola E, et al. Sex-Related Outcomes of Transcatheter Aortic Valve Implantation With Self-Expanding or Balloon-Expandable Valves: Insights from the OPERA-TAVI Registry. Am J Cardiol. 2024;219:60-70. doi:10.1016/j.amjcard.2024.01.028  Supplementary Figure 2 |  |
| **Dismorr M. et al 2024** | Dismorr M, Granbom-Koski M, Ellfors E, et al. Sex differences and long-term clinical outcomes after transcatheter aortic valve replacement: A SWEDEHEART study. Am Heart J. 2024;277:27-38. doi:10.1016/j.ahj.2024.07.018  Figure 2 |  |
| **Chikata Y. et al 2020** | Chikata Y, Iwata H, Doi S, Funamizu T, Okazaki S, Dohi S, Higuchi R, Saji M, Takamisawa I, Tamura H, et al. Simultaneous Estimation of Gender Male and Atrial Fibrillation as Risk Factors for Adverse Outcomes Following Transcatheter Aortic Valve Implantation. Journal of Clinical Medicine. 2020; 9(12):3963. https://doi.org/10.3390/jcm9123963  Supplementary Figure 1A |  |
| **Chikata Y. et al 2020** | Chikata Y, Iwata H, Doi S, Funamizu T, Okazaki S, Dohi S, Higuchi R, Saji M, Takamisawa I, Tamura H, et al. Simultaneous Estimation of Gender Male and Atrial Fibrillation as Risk Factors for Adverse Outcomes Following Transcatheter Aortic Valve Implantation. Journal of Clinical Medicine. 2020; 9(12):3963. https://doi.org/10.3390/jcm9123963  Supplementary Figure 1A |  |

# Supplementary Table S25: Landmark analysis of HF hospitalizations

# Supplementary Table S26: Cox Model Results: 00-HF hospitalizations

**A. Usual Cox Regression**

**Cox Model Coefficients & 95% Confidence Intervals**

| exp(coef) | exp(-coef) | lower .95 | upper .95 |
| --- | --- | --- | --- |
| 1.080672 | 0.9253506 | 1.004973 | 1.162072 |

**Schoenfeld Residuals Test**

| Variable | chisq | df | p |
| --- | --- | --- | --- |
| relevel(Arm, ref = placebb) | 8.27117 | 1 | 0.004027955 |
| GLOBAL | 8.27117 | 1 | 0.004027955 |

**Schoenfeld Residuals Plot**

**B. Stratified Cox Regression**

**Cox Model Coefficients & 95% Confidence Intervals**

| exp(coef) | exp(-coef) | lower .95 | upper .95 |
| --- | --- | --- | --- |
| 1.089139 | 0.9181563 | 1.012522 | 1.171554 |

**Schoenfeld Residuals Test**

| Variable | chisq | df | p |
| --- | --- | --- | --- |
| relevel(Arm, ref = placebb) | 6.522608 | 1 | 0.01065117 |
| GLOBAL | 6.522608 | 1 | 0.01065117 |

**Schoenfeld Residuals Plot**

# Supplementary Figure S1: PRISMA flowchart illustrating screening and selection of studies

# Supplementary Figure S2: All-cause mortality time-varying hazard ratio curve

# Supplementary Figure S3: Sensitivity Analysis of all-cause mortality restricted to studies with risk tables beneath KM plots

# Supplementary Figure S4: Sensitivity Analysis of all-cause mortality (pooling data from secondary analyses of RCTs only)

# Supplementary Figure S5: Subgroup Analysis of all-cause mortality by valve generation

# Supplementary Figure S9: Meta-regression of All-cause mortality using the average prevalence of comorbidities as covariates

# Supplementary Figure S10: Meta-regression of All-cause mortality using differences in comorbidities as covariates

# Supplementary Figure S6: All-cause mortality in East Asia

**Landmark analysis at 30 days:**

**HR: 1.041 (95% CI: 0.687 to 1.576, p = 0.8)**

# Supplementary Figure S7: All-cause mortality in Europe

**Landmark analysis at 30 days:**

**HR: 1.017 (95% CI: 0.935 to 1.107, p = 0.69)**

# Supplementary Figure S8: All-cause mortality in North America

**Landmark analysis at 30 days:**

**HR: 1.175 (95% CI: 1.075 to 1.283, p < 0.001)**

# Supplementary Figure S11: Geographical region-based subgroup analysis of sex differences in CV mortality

# Supplementary Figure S12: time-varying HR for stroke / TIA :

# Supplementary Figure S13: Sensitivity analysis of stroke using studies with risk tables beneath KM plots

# Supplementary Figure S14: Geographically based sex differences in stroke

# Supplementary figure S15: Myocardial infarction

# Supplementary Figure S16: All-cause rehospitalizations

# Supplementary Figure S17: Heart failure rehospitalizations

# Supplementary Figure S18: Forest Plot of Permanent Pacemaker Implantation:

# Supplementary Figure S19: Forest Plot of Acute Kidney Injury:

# Supplementary Figure S20: Forest Plot of Cardiac Tamponade:

# Supplementary Figure S21: Forest Plot of VARC-defined Bleeding:

# Supplementary Figure S22: Forest Plot of VARC-defined Vascular Complications:

# Supplementary Figure S23: Sensitivity Analysis of Permanent Pacemaker Implantation:

# Supplementary Figure S24: Sensitivity Analysis of Acute Kidney Injury:

# Supplementary Figure S25: Sensitivity Analysis of Cardiac Tamponade:

# Supplementary Figure S26: Sensitivity Analysis of VARC-defined Bleeding:

# Supplementary Figure S27: Sensitivity Analysis of VARC-defined Vascular Complications:

# Supplementary Figure S28: Funnel plot of Permanent Pacemaker Implantation:

# Supplementary Figure S29: Funnel plot of Acute Kidney Injury:

# Supplementary Figure S30: Funnel plot of Cardiac Tamponade:

# Supplementary Figure S31: Funnel plot of VARC-defined Bleeding:

# Supplementary Figure S32: Funnel plot of VARC-defined Vascular Complications:

# Supplementary Figure S33: Meta-regression analysis of VARC-defined life-threatening bleeding:

# Supplementary Figure S34: Meta-regression analysis of VARC-defined major bleeding:

# Supplementary Figure S35: Meta-regression analysis of VARC-defined major vascular complications:

# Supplementary Figure S36: geographical-region-based subgroup analysis of bleeding and vascular complications

# Supplementary Figure S37: Subgroup analysis of newer versus older generation valves for Life-threatening Bleeding

# Supplementary Figure S38: Subgroup analysis of newer versus older generation valves for Major Bleeding

# Supplementary Figure S39: Subgroup analysis of newer versus older generation valves for Major Vascular Complications

# Supplementary Figure S40: meta-regression of 30-day all-cause mortality (ACM) and early procedural complications:

# References

1.	Sticchi A, Grassini D, Gallo F, Benenati S, Kim WK, Khokhar AA, et al. Gender-Specific Outcomes in TAVI with Self-Expandable Valves: Insights from a Large Real-World Registry. J Clin Med. 2025;14(9).

2.	Al-Azizi K, Pickering T, AboHajar MB, Moubarak G, Mohiuddin A, Potluri SP, et al. Incidence and Sex-Based Comparison of Prosthesis-Patient Mismatch in Patients Undergoing Transcatheter Aortic Valve Replacement. Journal of the Society for Cardiovascular Angiography & Interventions.

3.	Matetic A, Kristić I, Crnčević N, Zanchi J, Domjanović Škopinić T, Baković Kramarić D, et al. Sex-specific anatomic differences in patients undergoing transcatheter aortic valve implantation: insights from the ST-TAVI registry. Hellenic J Cardiol. 2025.

4.	Adamo M, Branca L, Pezzola E, Saia F, Pilgrim T, Abdel-Wahab M, et al. Sex-Related Outcomes of Transcatheter Aortic Valve Implantation With Self-Expanding or Balloon-Expandable Valves: Insights from the OPERA-TAVI Registry. Am J Cardiol. 2024;219:60-70.

5.	Ahmad D, Sá MP, Makani A, Kliner D, Toma C, Christensen E, et al. Transcatheter Aortic Valve Replacement in Patients With Small Aortic Annuli: A Propensity-Matched Analysis of Gender-Based Outcomes. Am J Cardiol. 2024;227:75-82.

6.	Chiu CA, Chen PR, Li YJ, Hsieh CC, Yu HC, Chiu CC, et al. Female showed favorable left ventricle hypertrophy regression during post-TAVR follow-up. Kaohsiung J Med Sci. 2024;40(4):384-94.

7.	Dismorr M, Granbom-Koski M, Ellfors E, Rück A, Settergren M, Sartipy U, et al. Sex differences and long-term clinical outcomes after transcatheter aortic valve replacement: A SWEDEHEART study. Am Heart J. 2024;277:27-38.

8.	Gabani R, Brugaletta S, Bujak K, Pèrez-Vizcayno MJ, Jiménez-Quevedo P, Arévalos V, et al. Impact of gender on in-hospital and long-term outcomes after transcatheter aortic valve implantation: an analysis of the Spanish TAVI registry. Rev Esp Cardiol (Engl Ed). 2025;78(4):338-46.

9.	Iwata J, Hayashida K, Arita R, Moriizumi T, Kajino A, Sakata S, et al. Long-Term Impact of Gender Differences After Transcatheter Aortic Valve Implantation. CJC Open. 2024;6(12):1453-61.

10.	Kim M, Kang DY, Ahn JM, Kim JB, Yeung AC, Nishi T, et al. Sex-Specific Disparities in Clinical Outcomes After Transcatheter Aortic Valve Replacement Among Different Racial Populations. JACC Asia. 2024;4(4):292-302.

11.	Lodo V, Italiano EG, Weltert L, Zingarelli E, Perrucci C, Pietropaolo C, et al. The influence of gender on outcomes following transcatheter aortic valve implantation. Front Cardiovasc Med. 2024;11:1417430.

12.	Tateishi K, Hmoud H, De Gregorio I, Hastings R, De Gregorio J. The impact of gender on outcomes of transcatheter aortic valve implantation between self-expanding valve and balloon-expandable valve. Cardiovasc Revasc Med. 2024;63:38-42.

13.	Høydahl MP, Busund R, Rösner A, Kjønås D. Transcatheter aortic valve implantation (from inception to standard treatment): a single-center observational study. Front Cardiovasc Med. 2024;11:1298346.

14.	Zhou C, Xia Z, Chen B, Song Y, Lian Z. Gender Differences in Age-Stratified Early Outcomes in Patients With Transcatheter Aortic Valve Implantation. Am J Cardiol. 2023;187:100-9.

15.	van Bergeijk KH, van Ginkel DJ, Brouwer J, Nijenhuis VJ, van der Werf HW, van den Heuvel AFM, et al. Sex Differences in Outcomes After Transcatheter Aortic Valve Replacement: A POPular TAVI Subanalysis. JACC Cardiovasc Interv. 2023;16(9):1095-102.

16.	Leone PP, Gohar A, Pagnesi M, Mangieri A, Stefanini G, Cacia M, et al. Clinical outcomes in women and men with small aortic annuli undergoing transcatheter aortic valve implantation: A multicenter, retrospective, propensity score-matched comparison. Int J Cardiol. 2023;379:16-23.

17.	Malvindi PG, Bifulco O, Berretta P, Alfonsi J, Cefarelli M, Zingaro C, et al. Improved Early Outcomes in Women Undergoing Aortic Valve Interventions. J Clin Med. 2023;12(17).

18.	Elbaz-Greener G, Rahamim E, Abu Ghosh Z, Carasso S, Yarkoni M, Radhakrishnan S, et al. Sex difference and outcome trends following transcatheter aortic valve replacement. Front Cardiovasc Med. 2022;9:1013739.

19.	Alasnag M, Alanazi N, Al-Shaikh S, AlMerri K, Almoghairi A, Alenezi A, et al. WIN Gulf TAVR Registry: Describing Sex Differences in Patient Characteristics, Prognosis, and Outcomes. J Soc Cardiovasc Angiogr Interv. 2022;1(6):100509.

20.	Medranda GA, Rogers T, Case BC, Zhang C, Shea C, Satler LF, et al. Sex Disparities in Hemodynamics and Outcomes in Patients Who Underwent Contemporary Transcatheter Aortic Valve Implantation. Am J Cardiol. 2022;174:101-6.

21.	Patel PP, El Sabbagh A, Johnson PW, Suliman R, Salwa N, Morales-Lara AC, et al. Sex Differences in the Impact of Aortic Valve Calcium Score on Mortality After Transcatheter Aortic Valve Replacement. Circ Cardiovasc Imaging. 2022;15(8):e014034.

22.	Wöhrle J, Gilard M, Didier R, Kini A, Tavenier AH, Tijssen JGP, et al. Outcomes After Transcatheter Aortic Valve Implantation in Men Versus Women. Am J Cardiol. 2022;180:108-15.

23.	He JJ, Xiong TY, Yao YJ, Peng Y, Wei JF, He S, et al. Sex Difference in Outcomes Following Transcatheter Aortic Valve Replacement in Bicuspid Aortic Stenosis. JACC Cardiovasc Interv. 2022;15(16):1652-60.

24.	Kuneman JH, Butcher SC, Stassen J, Singh GK, Pio SM, van der Kley F, et al. Interaction between sex and left ventricular reverse remodeling and its association with outcomes after transcatheter aortic valve implantation. Int J Cardiovasc Imaging. 2022;38(9):1973-85.

25.	Chiam PTL, Hayashida K, Watanabe Y, Yin WH, Kao HL, Lee MKY, et al. Sex differences in patients undergoing transcatheter aortic valve replacement in Asia. Open Heart. 2021;8(1).

26.	Deharo P, Cuisset T, Bisson A, Herbert J, Lacour T, Etienne CS, et al. Outcomes Following Aortic Stenosis Treatment (Transcatheter vs Surgical Replacement) in Women vs Men (From a Nationwide Analysis). Am J Cardiol. 2021;154:67-77.

27.	Denegri A, Romano M, Petronio AS, Angelillis M, Giannini C, Fiorina C, et al. Gender Differences after Transcatheter Aortic Valve Replacement (TAVR): Insights from the Italian Clinical Service Project. J Cardiovasc Dev Dis. 2021;8(9).

28.	Gonçalves M, Teles RC, de Araújo Gonçalves P, de Sousa Almeida M, Félix de Oliveira A, Brito J, et al. Gender Differences and Mortality Trends After Transcatheter Aortic Valve Implantation: A 10-Year Analysis From a Single Tertiary Center. J Invasive Cardiol. 2021;33(6):E431-e42.

29.	Rodriguez-Gabella T, Zimarino M, Barbanti M, Testa L, Capodanno D, Stefanini GG, et al. Sex based analysis of the impact of red blood cell transfusion and vascular or bleeding complications related to TAVI - The TRITAVI-Women Study. Int J Cardiol. 2021;333:69-76.

30.	Shishido K, Yamanaka F, Ochiai T, Moriyama N, Yokoyama H, Yokota S, et al. Effect of Sex on Mortality and Left Ventricular Remodeling After Transcatheter Aortic Valve Implantation. Circ J. 2021;85(7):979-88.

31.	Simard T, Alqahtani F, Hibbert B, Mamas MA, El-Hajj S, Harris AH, et al. Sex-specific in-hospital outcomes of transcatheter aortic valve replacement with third generation transcatheter heart valves. Catheter Cardiovasc Interv. 2021;98(1):176-83.

32.	Useini D, Schlömicher M, Aweimer A, Haldenwang P, Strauch J, Patsalis P. Long-term Follow up after Transfemoral Transcatheter Aortic Valve Implantation in Lower Risk Patients Using the Balloon-Expandable Bioprosthesis: Gender-Dependent Outcomes. Heart Surg Forum. 2022;25(2):E175-e80.

33.	Chang HH, Chen PL, Leu HB, Chen IM, Wu NY, Chen YH. Sex difference in the prognostic role of body composition parameters in Taiwanese patients undergoing transcatheter aortic valve implantation. BMC Cardiovasc Disord. 2020;20(1):283.

34.	Chen SC, Leu HB, Chang HH, Chen IM, Chen PL, Lin SM, et al. Women had favourable reverse left ventricle remodelling after TAVR. Eur J Clin Invest. 2020;50(1):e13183.

35.	Stehli J, Dagan M, Zaman S, Koh JQS, Quine E, Gouskova N, et al. Impact of Gender on Transcatheter Aortic Valve Implantation Outcomes. Am J Cardiol. 2020;133:98-104.

36.	Tarantini G, Baumgartner H, Frank D, Husser O, Bleiziffer S, Rudolph T, et al. Four-year mortality in women and men after transfemoral transcatheter aortic valve implantation using the SAPIEN 3. Catheter Cardiovasc Interv. 2021;97(5):876-84.

37.	Wilczek K, Chodór P, Dyrbuś M, Hawranek M, Włoch Ł, Pyka Ł, et al. Female gender and the clinical and periprocedural profile and clinical outcomes of transcatheter aortic valve implantation: experiences of a tertiary Polish centre. Postepy Kardiol Interwencyjnej. 2020;16(4):436-43.

38.	Van Mieghem NM, Reardon MJ, Yakubov SJ, Heiser J, Merhi W, Windecker S, et al. Clinical outcomes of TAVI or SAVR in men and women with aortic stenosis at intermediate operative risk: a post hoc analysis of the randomised SURTAVI trial. EuroIntervention. 2020;16(10):833-41.

39.	Chikata Y, Iwata H, Doi S, Funamizu T, Okazaki S, Dohi S, et al. Simultaneous Estimation of Gender Male and Atrial Fibrillation as Risk Factors for Adverse Outcomes Following Transcatheter Aortic Valve Implantation. J Clin Med. 2020;9(12).

40.	Linke A, Schlotter F, Haussig S, Woitek FJ, Stachel G, Adam J, et al. Gender-dependent association of diabetes mellitus with mortality in patients undergoing transcatheter aortic valve replacement. Clin Res Cardiol. 2019;108(1):39-47.

41.	Pighi M, Piazza N, Martucci G, Lachapelle K, Perrault LP, Asgar AW, et al. Sex-Specific Determinants of Outcomes After Transcatheter Aortic Valve Replacement. Circ Cardiovasc Qual Outcomes. 2019;12(3):e005363.

42.	Wang TY, Gracia E, Callahan S, Bilfinger T, Tannous H, Pyo R, et al. Gender Disparities in Management and Outcomes Following Transcatheter Aortic Valve Implantation With Newer Generation Transcatheter Valves. Am J Cardiol. 2019;123(9):1489-93.

43.	Singh A, Musa TA, Treibel TA, Vassiliou VS, Captur G, Chin C, et al. Sex differences in left ventricular remodelling, myocardial fibrosis and mortality after aortic valve replacement. Heart. 2019;105(23):1818-24.

44.	Kilic A, Bianco V, Gleason TG, Lee JS, Schindler J, Navid F, et al. Longitudinal Outcomes of Women Undergoing Transcatheter Aortic Valve Replacement. Innovations (Phila). 2019;14(4):311-20.

45.	Kaier K, von Zur Mühlen C, Zirlik A, Schmoor C, Roth K, Bothe W, et al. Sex-Specific Differences in Outcome of Transcatheter or Surgical Aortic Valve Replacement. Can J Cardiol. 2018;34(8):992-8.

46.	Szerlip M, Gualano S, Holper E, Squiers JJ, White JM, Doshi D, et al. Sex-Specific Outcomes of Transcatheter Aortic Valve Replacement With the SAPIEN 3 Valve: Insights From the PARTNER II S3 High-Risk and Intermediate-Risk Cohorts. JACC Cardiovasc Interv. 2018;11(1):13-20.

47.	Yousif N, Obeid S, Binder R, Denegri A, Shahin M, Templin C, et al. Impact of gender on outcomes after transcatheter aortic valve implantation. J Geriatr Cardiol. 2018;15(6):394-400.

48.	Itzhaki Ben Zadok O, Kornowski R, Finkelstein A, Barbash I, Danenberg H, Segev A, et al. Temporal Trends in Gender-Related Differences and Outcomes in Patients Who Underwent Transcatheter Aortic Valve Implantation (from the Israeli Transcatheter Aortic Valve Implantation Multicenter Registry). Am J Cardiol. 2019;123(1):108-15.

49.	Bagienski M, Tokarek T, Wiktorowicz A, Dziewierz A, Rzeszutko L, Sorysz D, et al. Sex-related differences in clinical outcomes and quality of life after transcatheter aortic valve implantation for severe aortic stenosis. Postepy Kardiol Interwencyjnej. 2017;13(3):233-9.

50.	Bauer T, Möllmann H, Beckmann A, Ensminger S, Frerker C, Holzhey D, et al. Left ventricular function determines the survival benefit for women over men after transcatheter aortic valve implantation (TAVI). EuroIntervention. 2017;13(4):467-74.

51.	Czarnecki A, Qiu F, Koh M, Prasad TJ, Cantor WJ, Cheema AN, et al. Clinical outcomes after trans-catheter aortic valve replacement in men and women in Ontario, Canada. Catheter Cardiovasc Interv. 2017;90(3):486-94.

52.	Katz M, Carlos Bacelar Nunes Filho A, Caixeta A, Antonio Carvalho L, Sarmento-Leite R, Alves Lemos Neto P, et al. Gender-related differences on short- and long-term outcomes of patients undergoing transcatheter aortic valve implantation. Catheter Cardiovasc Interv. 2017;89(3):429-36.

53.	Chandrasekhar J, Dangas G, Yu J, Vemulapalli S, Suchindran S, Vora AN, et al. Sex-Based Differences in Outcomes With Transcatheter Aortic Valve Therapy: TVT Registry From 2011 to 2014. J Am Coll Cardiol. 2016;68(25):2733-44.

54.	Forrest JK, Adams DH, Popma JJ, Reardon MJ, Deeb GM, Yakubov SJ, et al. Transcatheter Aortic Valve Replacement in Women Versus Men (from the US CoreValve Trials). Am J Cardiol. 2016;118(3):396-402.

55.	Kodali S, Williams MR, Doshi D, Hahn RT, Humphries KH, Nkomo VT, et al. Sex-Specific Differences at Presentation and Outcomes Among Patients Undergoing Transcatheter Aortic Valve Replacement: A Cohort Study. Ann Intern Med. 2016;164(6):377-84.

56.	Gaglia MA, Jr., Lipinski MJ, Torguson R, Gai J, Ben-Dor I, Bernardo NL, et al. Comparison in Men Versus Women of Co-morbidities, Complications, and Outcomes After Transcatheter Aortic Valve Implantation for Severe Aortic Stenosis. Am J Cardiol. 2016;118(11):1692-7.

57.	Gilard M, Schlüter M, Snow TM, Dall'Ara G, Eltchaninoff H, Moat N, et al. The 2011-2012 pilot European Society of Cardiology Sentinel Registry of Transcatheter Aortic Valve Implantation: 12-month clinical outcomes. EuroIntervention. 2016;12(1):79-87.

58.	Codner P, Orvin K, Assali A, Sharony R, Vaknin-Assa H, Shapira Y, et al. Long-Term Outcomes for Patients With Severe Symptomatic Aortic Stenosis Treated With Transcatheter Aortic Valve Implantation. Am J Cardiol. 2015;116(9):1391-8.

59.	Al-Lamee R, Broyd C, Parker J, Davies JE, Mayet J, Sutaria N, et al. Influence of gender on clinical outcomes following transcatheter aortic valve implantation from the UK transcatheter aortic valve implantation registry and the National Institute for Cardiovascular Outcomes Research. Am J Cardiol. 2014;113(3):522-8.

60.	Conrotto F, D'Ascenzo F, Salizzoni S, Presbitero P, Agostoni P, Tamburino C, et al. A gender based analysis of predictors of all cause death after transcatheter aortic valve implantation. Am J Cardiol. 2014;114(8):1269-74.

61.	Madershahian N, Scherner M, Rudolph T, Slottosch I, Grundmann F, Kuhn E, et al. Impact of sex on the risk to develop contrast-induced nephropathy after transcatheter aortic valve implantation. Innovations (Phila). 2014;9(2):87-92; discussion

62.	Onorati F, D'Errigo P, Barbanti M, Rosato S, Covello RD, Maraschini A, et al. Different impact of sex on baseline characteristics and major periprocedural outcomes of transcatheter and surgical aortic valve interventions: Results of the multicenter Italian OBSERVANT Registry. J Thorac Cardiovasc Surg. 2014;147(5):1529-39.

63.	Sherif MA, Zahn R, Gerckens U, Sievert H, Eggebrecht H, Hambrecht R, et al. Effect of gender differences on 1-year mortality after transcatheter aortic valve implantation for severe aortic stenosis: results from a multicenter real-world registry. Clin Res Cardiol. 2014;103(8):613-20.

64.	Erez A, Segev A, Medvedofsky D, Fefer P, Raanani E, Goldenberg I, et al. Factors affecting survival in men versus women following transcatheter aortic valve implantation. Am J Cardiol. 2014;113(4):701-5.

65.	Buja P, Napodano M, Tamburino C, Petronio AS, Ettori F, Santoro G, et al. Comparison of variables in men versus women undergoing transcatheter aortic valve implantation for severe aortic stenosis (from Italian Multicenter CoreValve registry). Am J Cardiol. 2013;111(1):88-93.

66.	D'Ascenzo F, Gonella A, Moretti C, Omedè P, Salizzoni S, La Torre M, et al. Gender differences in patients undergoing TAVI: a multicentre study. EuroIntervention. 2013;9(3):367-72.

67.	Ferrante G, Pagnotta P, Petronio AS, Bedogni F, Brambilla N, Fiorina C, et al. Sex differences in postprocedural aortic regurgitation and mid-term mortality after transcatheter aortic valve implantation. Catheter Cardiovasc Interv. 2014;84(2):264-71.

68.	Hayashida K, Morice MC, Chevalier B, Hovasse T, Romano M, Garot P, et al. Sex-related differences in clinical presentation and outcome of transcatheter aortic valve implantation for severe aortic stenosis. J Am Coll Cardiol. 2012;59(6):566-71.

69.	Humphries KH, Toggweiler S, Rodés-Cabau J, Nombela-Franco L, Dumont E, Wood DA, et al. Sex differences in mortality after transcatheter aortic valve replacement for severe aortic stenosis. J Am Coll Cardiol. 2012;60(10):882-6.

70.	Stangl V, Baldenhofer G, Knebel F, Zhang K, Sanad W, Spethmann S, et al. Impact of gender on three-month outcome and left ventricular remodeling after transfemoral transcatheter aortic valve implantation. Am J Cardiol. 2012;110(6):884-90.

71.	Buchanan GL, Chieffo A, Montorfano M, Maisano F, Latib A, Godino C, et al. The role of sex on VARC outcomes following transcatheter aortic valve implantation with both Edwards SAPIEN™ and Medtronic CoreValve ReValving System® devices: the Milan registry. EuroIntervention. 2011;7(5):556-63.
